# Disordered Gibbs measures and Gaussian conditioning

**DOI:** 10.1007/s00440-026-01526-5

**Published:** 2026-08-22

**Authors:** Amir Dembo, Eliran Subag

**Affiliations:** 1 Stanford University, Stanford, California, USA; 2 Weizmann Institute of Science, Rehovot, Israel

**Keywords:** 82D30, 82B44, 60G15, 60G60

## Abstract

We study the law of a random field $$f_N(\boldsymbol{\sigma })$$ evaluated at a random sample from the Gibbs measure associated to a Gaussian field $$H_N(\boldsymbol{\sigma })$$. In the high-temperature regime, we show that bounds on the probability that $$f_N(\boldsymbol{\sigma })\in A$$ for $$\boldsymbol{\sigma }$$ randomly sampled from the Gibbs measure can be deduced from similar bounds for deterministic $$\boldsymbol{\sigma }$$ under the conditional Gaussian law given that $$H_N(\boldsymbol{\sigma })/N=E$$ for *E* close to the derivative $F^{\prime }\left(\beta \right)$ of the free energy (which is the typical value of $$H_N(\boldsymbol{\sigma })/N$$ under the Gibbs measure). In the more challenging low-temperature regime we restrict to *k*-rsb spherical spin glasses, proving a similar result, now with a more elaborate conditioning. Namely, with $$q_i$$ denoting the locations of the non-zero atoms of the Parisi measure, in addition to specifying that $$H_N(\boldsymbol{\sigma })/N=E$$, here one needs to also condition on the energy and its gradient at points $$\textbf{x}_1,\ldots ,\textbf{x}_k$$ such that $$\langle \textbf{x}_i,\textbf{x}_j\rangle /N=q_{i\wedge j}$$ and $$\langle \textbf{x}_i,\boldsymbol{\sigma }\rangle /N\approx q_{i}$$. Like in the high-temperature phase, the energy and gradient values on which one conditions are also specified by the model’s Parisi measure. We apply our general results to two important problems from statistical physics. That is, computing the Franz-Parisi potential at any temperature and, reducing certain asymptotics of Langevin dynamics with initial conditions distributed according to the Gibbs measure, to the more manageable problem of studying dynamics with non-random initial conditions and conditional disorder.

## Introduction and main results

For any N≥1, let $(\Sigma _{N},\mathcal {F}_{N},\mu _{N})$ be a probability (configuration) space, with a random *Hamiltonian* $H_{N}:\Sigma _{N}\rightarrow \mathbb {R}$ which is a centered Gaussian field on an auxiliary probability space, denoted hereafter by $\mathbb {P}$ (our standing assumption is that $\mathbb {P}$-a.s. any function on $\Sigma _{N}$ we encounter, is $\mathcal {F}_{N}$-measurable). For any inverse-temperature $\beta \ge 0$, define the partition function and free energy by

$$\begin{aligned} Z_{N,\beta }:=\int _{\Sigma _{N}}e^{\beta H_{N}(\boldsymbol{\sigma })}d\mu _{N}(\boldsymbol{\sigma }),\qquad F_{N,\beta }:=\frac{1}{N}\log Z_{N,\beta } \end{aligned}$$

and the (random) Gibbs measure by its density

$$ \frac{dG_{N,\beta }}{d\mu _{N}}(\boldsymbol{\sigma })=\frac{e^{\beta H_{N}(\boldsymbol{\sigma })}}{Z_{N,\beta }}. $$

Suppose $f_{N}:\Sigma _{N}\rightarrow \mathbb {R}$ is another random field, possibly depending on $H_{N}$ in a complicated way. In this work we study the asymptotic behavior as $N \rightarrow \infty$ of such $f_{N}(\boldsymbol{\sigma })$ evaluated at a random sample $\boldsymbol{\sigma }$ from the Gibbs measure $G_{N,\beta }$. Precisely, for Borel $A\subset \mathbb {R}$ we wish to understand random variables of the form

1.1 $$\begin{aligned} G_{N,\beta }\left( f_{N}(\boldsymbol{\sigma })\in A\right) =\frac{\int _{\{\boldsymbol{\sigma }:\,f_{N}(\boldsymbol{\sigma })\in A\}}e^{\beta H_{N}(\boldsymbol{\sigma })}d\mu _{N}(\boldsymbol{\sigma })}{\int _{\Sigma _{N}}e^{\beta H_{N}(\boldsymbol{\sigma })}d\mu _{N}(\boldsymbol{\sigma })}. \end{aligned}$$

One often expects $f_{N}(\boldsymbol{\sigma })$ to asymptotically concentrate under the Gibbs measure around a specific value (i.e. its median). Namely, that for some value $E\in \mathbb {R}$ and any ε>0,

1.2 $$\begin{aligned} \lim _{N\rightarrow \infty }\mathbb {E}G_{N,\beta }\Big ( \big |f_N(\boldsymbol{\sigma })-E\big |>\varepsilon \Big )=0. \end{aligned}$$

The classical example for this is the normalized “energy” $f_{N}=\frac{1}{N}H_{N}$, whose average under the Gibbs measure is the derivative of the free energy,

1.3 $$\begin{aligned} \frac{d}{d\beta }F_{N,\beta } = \frac{\int _{\Sigma _{N}}\frac{1}{N}H_N(\boldsymbol{\sigma }) e^{\beta H_{N}(\boldsymbol{\sigma })}d\mu _{N}(\boldsymbol{\sigma })}{\int _{\Sigma _{N}}e^{\beta H_{N}(\boldsymbol{\sigma })}d\mu _{N}(\boldsymbol{\sigma })}. \end{aligned}$$

Respectively, in the large *N* limit, if for some deterministic, differentiable function *F* and any β,

1.4 $$\begin{aligned} \lim _{N\rightarrow \infty }\mathbb {E}F_{N,\beta }=F(\beta ), \end{aligned}$$

then under the Gibbs measure $$\frac{1}{N}H_{N}(\boldsymbol{\sigma })$$ concentrates around $E=F^{\prime }\left(\beta \right)$ in the sense of (1.2), see [5].

What about more general functions $f_{N}$, possibly depending on $H_{N}$ in a complicated way? From a statistical physics perspective, this question is fundamental: we wish to understand the behavior of a general “observable” $f_N$ at a configuration $\boldsymbol{\sigma }$ drawn from the thermal equilibrium distribution. Indeed, below we discuss two very well-known objects from spin glass theory that fit into this general framework. On the other hand, mathematically this question is closely related to understanding how the random function $H_N$ looks like around a point $\boldsymbol{\sigma }$ sampled from a given height. Indeed, the latter amounts to understanding $f_N(\boldsymbol{\sigma })=\bar{f}_N(\boldsymbol{\sigma }, H_N(\cdot ))$ for deterministic functionals $\bar{f}$, while sampling $\boldsymbol{\sigma }$ from the Gibbs measure approximates the uniform measure on the set of points with energy around a given level thanks to concentration of $\frac{1}{N} H_N(\boldsymbol{\sigma })$.

A naive approach may suggest to modify the definition of the free energy by replacing $\beta H_N(\boldsymbol{\sigma })$ by $\beta H_N(\boldsymbol{\sigma })+\alpha N f_N(\boldsymbol{\sigma })$, so that similarly to (1.3) the $\frac{d}{d\alpha }|_{\alpha =0}$ derivative is the average of $f_N(\boldsymbol{\sigma })$ under $G_{N,\beta }$. One then hopes to show that $f_N(\boldsymbol{\sigma })$ concentrates around the derivative of the $N\rightarrow \infty$ limit of this modified free energy, as in the case of $\frac{1}{N}H_N(\boldsymbol{\sigma })$. Even if we ignore the issue of concentration, for many choices of $f_N$ of interest, it is impossible to analytically compute such modified free energy. In contrast, our main results provide a general tool for arbitrary $f_N$ (modulo regularity conditions) to deduce concentration under the Gibbs measure around a specific value *E*, from analogous statements about deterministic $\boldsymbol{\sigma }$, under a modified Gaussian disorder arising from a suitable conditioning. Excluding specific examples like $f_N=\frac{1}{N} H_N$ above, these results are the first to provide a general principle for concentration under the Gibbs measure for the low temperature phase of spin glasses.

We shall consider two settings. The first allows for a very general choice of the configuration space and Hamiltonian, at the price of restricting to the easier regime of small β. We shall see that the behavior under the Gibbs measure can be described by the conditional law of $f_{N}(\boldsymbol{\sigma })$ given that the energy is equal to its typical value $\frac{1}{N}H_{N}(\boldsymbol{\sigma })=F'(\beta )$. The proof exploits the fact that in this regime $\mathbb {E}F_{N,\beta }=\frac{1}{N} \log \mathbb {E}Z_{N,\beta }+o(1)$. The argument we employ is related to a technique used in [1] for random constraint satisfaction problems. We present the result in Section 1.1 and its short proof in Section 4, setting the stage for our main theorem (for large β) by demonstrating how the conditional Gaussian field naturally arises.

In the second setting we consider the more challenging problem of large values of β. Here we shall restrict to spherical spin glass models with *k*-step replica symmetry breaking (rsb), for some *k* finite. As in the case of small β, we translate questions about the behavior under the Gibbs measure to ones about $f_{N}(\boldsymbol{\sigma })$ for deterministic $\boldsymbol{\sigma }$, under an appropriate conditional Gaussian law. However, large β requires a more elaborate conditioning that involves additional points $\textbf{x}_1,\ldots ,\textbf{x}_k$ with a specific geometry. Such $\textbf{x}_i$ are conditioned to be critical points on appropriate spheres and further to specifying the values of $H_N$ at $\boldsymbol{\sigma }$ we specify its values and ‘radial’ derivatives at all $\textbf{x}_1,\ldots ,\textbf{x}_k$. We present this result in Section 1.2.

For mean-field spin glasses, the Gibbs measure $$G_{N,\beta }$$ is well understood in the large *N* limit. Namely, its asymptotic structure is explicitly characterized by Ruelle probability cascades, see [36]. By itself, however, the latter is insufficient to analyze $$f_N(\boldsymbol{\sigma })$$ under $$G_{N,\beta }$$. Indeed, thanks to the concentration of the normalized energy $$\frac{1}{N} H_N(\boldsymbol{\sigma })$$ mentioned above, $$G_{N,\beta }$$ essentially only depends on the structure of the Hamiltonian $$H_N$$ at energy levels close to $F^{\prime }\left(\beta \right)$, while the value of $$f_N(\boldsymbol{\sigma })$$, even for $$\boldsymbol{\sigma }$$ sampled from $$G_{N,\beta }$$, may depend (and does in interesting examples) in a complicated way on the structure of $$H_N$$ outside of such regions. Our proof for the low-temperature phase of large $$\beta \ge 0$$ exploits the pure states decompositions of Talagrand [46] and Jagannath [31] to relate the Gibbs measure to the aforementioned critical points $$\textbf{x}_1,\ldots ,\textbf{x}_k$$. Thanks to the ultrametricity property proved by Panchenko [35], the pure states are organized in a tree structure, which can be given a geometric interpretation by an embedding in the interior of the sphere in $$\mathbb {R}^N$$. In [42] it was shown that each vertex $$\textbf{x}$$ in the tree roughly maximizes the energy over the sphere of radius $$\Vert \textbf{x}\Vert $$ and our Proposition 3.1, which is of independent interest, shows that for *k*-rsb models, there exists such a decomposition where the vertices are also critical points in an appropriate sense (see Section 8 for its proof). This allows us to relate averages over the Gibbs measure to ones over the relevant critical points and express the latter in terms of the conditional Gaussian law by combining a novel, multi-level Kac-Rice formula (see Proposition 7.1), with the available information on the complexity of such critical points from a recent work of Huang and Sellke [30] (which relates the annealed complexity to the Parisi measure).

It is plausible to carry out such a program also for other disordered mean-field models, involving multi-species, structured external fields (signals), or Ising spins (after improving our understanding of the complexity considerations associated with the pure-state decomposition).

Franz and Parisi introduced in [27] a certain “potential” as a tool to relate dynamical properties to the statics of the spherical spin glass model. The idea here is to sample a point $\boldsymbol{\sigma }$ from the Gibbs measure and calculate, at an exponential scale, the Gibbs probability of sampling another point $\boldsymbol{\sigma }'$ whose overlap with $\boldsymbol{\sigma }$ is approximately *r*. In Section 2.2 we compute the Franz-Parisi potential at any temperature, by applying both our result for the high temperature phase and for the low temperature phase. To the best of our knowledge, this is *the first rigorous computation of the Franz-Parisi potential at low temperature,* for any spherical model. Restricting for simplicity the low temperature phase to 1-rsb models, our formula (2.16) involves a maximization over the possible values of the overlap ρ between $\boldsymbol{\sigma }'$ and $\textbf{x}_1$. We thus expect the latter parameter, and in particular the continuity in *r* of the argmax in (2.16), or the lack thereof, to be highly relevant for the asymptotic behavior of certain natural dynamics which are reversible wrt $G_{N,\beta }$. Similarly, the formula for the Franz-Parisi potential of *k*-rsb models involves the maximization over the overlaps between $\boldsymbol{\sigma }'$ and $\textbf{x}_i$, $1 \le i \le k$.

Another important application of this work is to the study of the Langevin dynamics $(\textbf{x}_t, t \ge 0)$ at inverse-temperature $\widetilde{\beta }$ for spherical mixed *p*-spin Hamiltonians $H_N$. That is,

1.5 $$\begin{aligned} \textbf{x}_t=\textbf{x}_0-\int _0^t b_\ell '(\vert \vert \textbf{x}_u\vert \vert ^2/N)\textbf{x}_u du + \frac{1}{2}\widetilde{\beta }\int _0^t \nabla H_N(\textbf{x}_u) du + \textbf{B}_t, \end{aligned}$$

where $\textbf{B}_t$ is a standard *N*-dimensional Brownian motion and the soft constraint potentials $b_\ell (\cdot )$ concentrate at one as $\ell \rightarrow \infty$, thereby converting the $\mathbb {R}^N$-valued diffusion (1.5) to the spherical Langevin dynamic, reversible wrt the random Gibbs measure $G_{N,\widetilde{\beta }}$. Such disordered Langevin dynamics often exhibit very different behavior at various time-scales, including *aging* behavior (where the older it gets, the longer the system takes to forget its current state), and there is much interest in these dynamics in out-of-equilibrium statistical physics (cf. [14, 19, 21, 22] and the references therein). In particular, for Gibbs initial conditions, namely, with $\textbf{x}_0 =\boldsymbol{\sigma }$ drawn from $G_{N,\beta }$, possibly at $\beta \ne \widetilde{\beta }$. For $\textbf{x}_0$ uniformly distributed on the sphere, the thermodynamic limit as $N \rightarrow \infty$ (and then $\ell \rightarrow \infty$), while *t*, *s* are fixed, of the empirical covariance, the integrated response functions and the normalized potential, namely,

1.6 $$\begin{aligned} C_N(s,t) := \frac{1}{N} \langle \textbf{x}_s,\textbf{x}_t\rangle , \quad \chi _N(s,t):=\frac{1}{N} \langle \textbf{x}_s, \textbf{B}_t \rangle \quad \textrm{and} \quad \widetilde{H}_N(s):=\frac{1}{N} H_N(\textbf{x}_s), \end{aligned}$$

respectively, satisfy the ckchs-equations of Cugliandolo-Kurchan [21] and Crisanti-Horner-Sommers [19]. The ckchs-equations have been rigorously derived in [11], but prior to our work there has been no such result for (1.5) with Gibbs initial conditions. It was *not even known what the thermodynamic limit might then be*, except for pure *p*-spins at the replica symmetric high temperature ($\beta < \beta _c$), where using the non-rigorous replica method, [8, 9] predict such limit equations for $(C_N,\chi _N,\widetilde{H}_N)$. The challenge to merely predicting what the limit ought to be at low temperatures, stems from the lack of any representation of $(C_N,\chi _N,\widetilde{H}_N)$ (or their expected value), as explicit functionals of the random Hamiltonian. We show in Section 2.1 how our results reduce having Gibbs initial conditions within the finite rsb phase, to the much easier study of (1.5) for non-random $\textbf{x}_0$ with suitable linear constraints imposed on the Hamiltonian. Indeed, taking advantage of this reduction allows us to rigorously derive in [24] the thermodynamic limit of $(C_N,\chi _N,\widetilde{H}_N)$ for any Gibbsian initial conditions within the 1-rsb phase (the same applies for the *k*-rsb phase, apart from having then a more complex limiting system of equations and a technically more involved derivation).

### High temperature phase

Assume that the normalized variance $\frac{1}{N}\mathbb {E}\big [H_{N}(\boldsymbol{\sigma })^{2}\big ]$ is bounded uniformly in N≥1 and $\boldsymbol{\sigma }\in \Sigma _{N}$. For simplicity, we further assume that it is essentially constant over space, in the sense that for any ε>0,

1.7 $$\begin{aligned} \varlimsup _{N\rightarrow \infty }\frac{1}{N}\log \mu _{N}\Big (\boldsymbol{\sigma }:\,\Big |\frac{1}{N}\mathbb {E}\big [H_{N}(\boldsymbol{\sigma })^{2}\big ]-1\Big |>\varepsilon \Big )=-\infty . \end{aligned}$$

We consider small values of β, for which the analysis is simpler. Namely, we work here in the setting where whp,

$$ \frac{1}{N}\log Z_{N,\beta }=\frac{1}{N}\log \mathbb {E}Z_{N,\beta }+o(1). $$

Of course,

$$ \mathbb {E}Z_{N,\beta }=\int _{\Sigma _{N}}\mathbb {E}\big [e^{\beta H_{N}(\boldsymbol{\sigma })}\big ] d\mu _{N}(\boldsymbol{\sigma })=\int _{\Sigma _{N}}e^{\frac{1}{2}\beta ^{2}\mathbb {E}\big [H_{N}(\boldsymbol{\sigma })^{2}\big ]}d\mu _{N}(\boldsymbol{\sigma }) $$

and (1.7) thus implies that, for all β,

$$\begin{aligned} \lim _{N\rightarrow \infty }\frac{1}{N}\log \mathbb {E}Z_{N,\beta }=\frac{1}{2}\beta ^{2}. \end{aligned}$$

#### Remark 1.1

We define the critical inverse-temperature $\beta _{c}$ as the unique value such that^1^

1.8 $$\begin{aligned} \lim _{N\rightarrow \infty }\mathbb {E}F_{N,\beta }=\frac{1}{2}\beta ^{2}\iff \beta \le \beta _{c}. \end{aligned}$$

For any fixed $\boldsymbol{\sigma }\in \Sigma _N$ and Borel $A \subset \mathbb {R}$, the sub-probability measure on $\mathbb {R}$

$$ \mathbb {P}\big (f_{N}(\boldsymbol{\sigma })\in A,\,\frac{1}{N}H_{N}(\boldsymbol{\sigma })\in \cdot \big ) $$

is absolutely continuous wrt the Lebesgue measure allowing us to define the conditional probability

1.9 $$\begin{aligned} p_N(\boldsymbol{\sigma },E) := \mathbb {P}\big (f_{N}(\boldsymbol{\sigma })\in A\,\big |\,\frac{1}{N}H_{N}(\boldsymbol{\sigma })=E \big ) \end{aligned}$$

as the corresponding Radon-Nikodym derivative, normalized by the positive Gaussian density of $\frac{1}{N}H_{N}(\boldsymbol{\sigma })$ at *E*. We assume hereafter that $p_N$ of (1.9) are measurable on $\Sigma _N \times \mathbb {R}$ equipped with the product measure of $\mu _N$ and Lebesgue.

#### Theorem 1.2

Assume (1.7) and let $\beta \le \beta _{c}$. Suppose the $\mathbb {P}$-a.s. $\mathcal {F}_{N}$-measurable random field $f_{N}(\boldsymbol{\sigma })$ and Borel $A\subset \mathbb {R}$ are such that $p_N(\boldsymbol{\sigma },E)$ of (1.9) is measurable on $\Sigma _N \times \mathbb {R}$. If

1.10 $$\begin{aligned} \lim _{\varepsilon \rightarrow 0}\varlimsup _{N\rightarrow \infty }\frac{1}{N}\log \int _{\Sigma _{N}}\int _{\beta -\varepsilon }^{\beta +\varepsilon } \mathbb {P}\big (f_{N}(\boldsymbol{\sigma })\in A\,\big |\,\frac{1}{N}H_{N}(\boldsymbol{\sigma })=E \big ) dE d\mu _{N}(\boldsymbol{\sigma })<0, \end{aligned}$$

then

1.11 $$\begin{aligned} \varlimsup _{N\rightarrow \infty }\frac{1}{N}\log \mathbb {E}\Big [ G_{N,\beta }\left( f_{N}(\boldsymbol{\sigma })\in A\right) \Big ] <0. \end{aligned}$$

Of course, (1.10) follows whenever

1.12 $$\begin{aligned} \lim _{\varepsilon \rightarrow 0}\varlimsup _{N\rightarrow \infty }\frac{1}{N}\log \mathop {\mathrm {ess\,sup}}\limits _{\boldsymbol{\sigma }\in \Sigma _{N},\,|E-\beta |<\varepsilon }\mathbb {P}\Big (f_{N}(\boldsymbol{\sigma })\in A\,\Big |\,\frac{1}{N}H_{N}(\boldsymbol{\sigma })=E\Big )<0\,. \end{aligned}$$

#### Remark 1.3

Often the probabilities in (1.10) do not depend on $\boldsymbol{\sigma }\in \Sigma _{N}$ so it suffices to verify the relevant exponential decay only at one fixed, convenient choice of $\boldsymbol{\sigma }$. For example, for the mixed *p*-spin models that we discuss in the sequel, with either Ising or spherical spins, the variance $\mathbb {E}[H_{N}(\boldsymbol{\sigma })^{2}]$ is constant on $\Sigma _{N}$ and the free energy converges [29, 41]. In the absence of an external field, $\beta _{c}>0$ is the inverse-temperature where replica symmetry is broken (excluding the special case of the spherical 2-spin), and $H_{N}(\boldsymbol{\sigma })$ is invariant under rotations. Thus, if $f_{N}(\boldsymbol{\sigma })$ is such that $\boldsymbol{\sigma }\mapsto (H_{N}(\boldsymbol{\sigma }),f_{N}(\boldsymbol{\sigma }))$ is also invariant, for example as for the Franz-Parisi potential we consider in Section 2.2, then the conditional probabilities in (1.10) are indeed constant over $\Sigma _{N}$.

The proof of Theorem 1.2 in Section 4, is under the above general setting. However, understanding the Gibbs measure for large β is an extremely challenging problem even for specific models, so in the rest of the paper we always assume that $G_{N,\beta }$ corresponds to some spherical spin glass model.

### Low temperature phase: spherical spin glasses

As mentioned above, we now restrict our attention to the spherical mixed *p*-spin models, for which we are able to exploit results relating the Gibbs measure to critical points [3, 4, 13, 39, 40, 43, 44] and the generalized Thouless-Anderson-Palmer (tap) approach [16, 17, 42, 47]. Specifically, we take the configuration space

$$\begin{aligned} \Sigma _{N}=\mathbb {S}^{N-1}:=\Big \{\boldsymbol{\sigma }=(\sigma _{1},\ldots ,\sigma _{N})\in \mathbb {R}^{N}:\,\Vert \boldsymbol{\sigma }\Vert =\sqrt{N}\,\Big \} \end{aligned}$$

with its Borel σ-algebra $\mathcal {F}_{N}$ and uniform measure $\mu _{N}$. For deterministic $\gamma _{p}\ge 0$, we have the corresponding *mixture*

1.13 $$\begin{aligned} \xi (t):=\sum _{p\ge 2} \gamma _{p}^{2}t^{p} \,, \end{aligned}$$

and we assume hereafter that $\xi (1+\varepsilon )<\infty$ for some $\varepsilon =\varepsilon _\xi >0$. The model with $\xi (t)=t^p$ is called the pure *p*-spin model, whereas a mixture $\xi (\cdot )$ is called generic if

$$\begin{aligned} \sum _{p\,\text {odd}}p^{-1}1\!\!1\{\gamma _{p}>0\}=\sum _{p\,\text {even}}p^{-1}1\!\!1\{\gamma _{p}>0\}=\infty . \end{aligned}$$

The mixed *p*-spin Hamiltonian on $\mathbb {S}^{N-1}$ corresponding to such ξ is given by

1.14 $$\begin{aligned} H_{N}(\boldsymbol{\sigma })=\sum _{p=2}^{\infty }\gamma _{p}N^{-\frac{p-1}{2}}\sum _{i_{1},\dots ,i_{p}=1}^{N}J_{i_{1},\dots ,i_{p}}\sigma _{i_{1}}\cdots \sigma _{i_{p}}, \end{aligned}$$

where $J_{i_{1},\dots ,i_{p}}$ are iid standard normal variables, yielding the covariance function

$$ \mathbb {E}H_{N}(\boldsymbol{\sigma })H_{N}(\boldsymbol{\sigma }')=N\xi (\langle \boldsymbol{\sigma },\boldsymbol{\sigma }'\rangle /N), $$

where $\frac{1}{N}\langle \boldsymbol{\sigma },\boldsymbol{\sigma }'\rangle :=\frac{1}{N}\sum _{i\le N}\sigma _{i}\sigma _{i}'$ is called the overlap of $\boldsymbol{\sigma }$ and $\boldsymbol{\sigma }'$.

The free energy of such spherical models is given by the Parisi formula [37, 38],

$$\begin{aligned} \lim _{N\rightarrow \infty }\mathbb {E}F_{N,\beta }=F(\beta )=\min _{x}\mathcal {P}_{\xi ,\beta }(x) \end{aligned}$$

rigorously proved by Talagrand [45] and Chen [15]. Here the minimum is taken over all [0,1]→[0,1] distribution functions (i.e., non-decreasing and right-continuous) such that $x(\hat{q})=1$ for some $\hat{q}<1$ and the Crisanti-Sommers [20] functional $\mathcal {P}_{\xi ,\beta }(x)$ is given by

1.15 $$\begin{aligned} \mathcal {P}_{\xi ,\beta }(x)=\frac{1}{2}\Big ( \beta ^2\int _0^1x(t)\xi '(t)dt+\int _0^{\hat{q}}\frac{dt}{\int _t^1 x(s)ds}+\log (1-\hat{q}) \Big ). \end{aligned}$$

The unique minimizer of the strictly convex functional (1.15) is called the Parisi distribution and denoted by $x_{P}=x_{\beta ,P}$. The corresponding measure $\nu _{P}=\nu _{\beta ,P}$ given by $\nu _{P}([0,q])=x_{P}(q)$ is called the Parisi measure.

A zero-temperature analogue of the Parisi formula expressing the ground state energy as

1.16 $$\begin{aligned} \lim _{N\rightarrow \infty }\frac{1}{N}\mathbb {E}\Big [ \sup _{\textbf{x}\in \mathbb {S}^{N-1}}\big \{ H_{N}(\textbf{x})\big \} \Big ] = \min _{\alpha ,c}\mathcal {P}_{\xi ,\infty }(\alpha ,c) \end{aligned}$$

was also proved in [18, 32]. Here the minimum is taken over all non-decreasing, right-continuous and integrable functions $\alpha :[0,1)\rightarrow [0,\infty )$ and real, positive values c>0, where

$$ \mathcal {P}_{\xi ,\infty }(\alpha ,c) := \frac{1}{2}\Big [ \xi '(1)c + \int _0^1 \xi '(t)\alpha (t)dt + \int _0^1\frac{dt}{\int _t^1 \alpha (s)ds+c}\, \Big ]\,. $$

We denote the ground state energy at radius $\sqrt{Nq}>0$ and (twice) its derivative by

1.17 $$\begin{aligned} E_{\star }(q)&:=\lim _{N\rightarrow \infty }\frac{1}{N}\mathbb {E}\Big [ \sup _{\textbf{x}\in \sqrt{q}\mathbb {S}^{N-1}}\big \{ H_{N}(\textbf{x})\big \} \Big ],\qquad R_{\star }(q):=2\frac{d}{dq}E_{\star }(q). \end{aligned}$$

Denoting by $(\alpha _q,c_q)$ the minimizer of (1.16) for the mixture $\hat{\xi }_q(t):= \xi (qt)$, we have that

1.18 $$\begin{aligned} E_{\star }(q)&= \mathcal {P}_{\hat{\xi }_q,\infty }(\alpha _q,c_q),\qquad R_{\star }(q) = \frac{c_q}{q}(\hat{\xi }_q''(1)+\hat{\xi }_q'(1)) +\frac{1}{q} \int _0^1(s\hat{\xi }_q''(s)\nonumber \\&\quad +\hat{\xi }_q'(s))\alpha _q(s)ds. \end{aligned}$$

The first equality follows from (1.16), since up to space scaling (by $\sqrt{q}$), the restriction of $H_N(\textbf{x})$ to $\sqrt{q}\mathbb {S}^{N-1}$ is the Hamiltonian on $\mathbb {S}^{N-1}$ for mixture $\hat{\xi }_q(t)$ (of the form $\sum _{p\ge 2}\gamma _pq^{\frac{p}{2}}H_N^p(\boldsymbol{\sigma })$ with independent pure *p*-spin Hamiltonians $H_N^p(\boldsymbol{\sigma })$). For the second equality, fix $\lambda ,q_1,q_2 \in [0,1]$ and note that since $(\lambda q_1+(1-\lambda ) q_2)^{\frac{p}{2}} \le \lambda q_1^{\frac{p}{2}}+(1-\lambda ) q_2^{\frac{p}{2}}$ for all p≥2, it follows that

$$\begin{aligned}&\mathbb {E}\max _{\boldsymbol{\sigma }} \sum _{p\ge 2}\gamma _p(\lambda q_1+(1-\lambda ) q_2)^{\frac{p}{2}}H_N^p(\boldsymbol{\sigma })\\&\le \mathbb {E}\max _{\boldsymbol{\sigma }} \sum _{p\ge 2}\gamma _p(\lambda q_1^{\frac{p}{2}}+(1-\lambda ) q_2^{\frac{p}{2}})H_N^p(\boldsymbol{\sigma })\\&\le \lambda \mathbb {E}\max _{\boldsymbol{\sigma }} \sum _{p\ge 2}\gamma _p q_1^{\frac{p}{2}}H_N^p(\boldsymbol{\sigma })+(1-\lambda )\mathbb {E}\max _{\boldsymbol{\sigma }} \sum _{p\ge 2}\gamma _p q_2^{\frac{p}{2}}H_N^p(\boldsymbol{\sigma }), \end{aligned}$$

from which we deduce that $q \mapsto E_{\star }(q)$ is convex. Further, comparing (1.16) with the lhs of (1.17) we see that

$$E_{\star }(q')\le \mathcal {P}_{\hat{\xi }_{q'},\infty }(\alpha _q,c_q)$$, with equality if $q^{\prime }=q$ (by the first equality of (1.18)). Thus,^2^

$$ R_{\star }(q) = 2\frac{d}{dq}E_{\star }(q)=2\Big [\frac{d}{dq}\mathcal {P}_{\hat{\xi }_q,\infty }(\alpha ,c)\Big ]\Big |_{(\alpha ,c)=(\alpha _q,c_q)} $$

which coincides with the rhs of the second equality in (1.18). Recall also that by [42, Proposition 11], for any positive $q\in \text{ supp }(\nu _{\beta ,P})$,

$$ \alpha _q(t)=\beta x_P(qt)\quad \text{ and }\quad c_q=\frac{\beta }{q}\int _q^1 x_P(s)ds $$

with $E_{\star }(q)$ and $R_{\star }(q)$ thus expressed directly in terms of the Parisi distribution $x_{P}$.

Given a probability measure ν on [0, 1] with cumulative distribution x(q)=ν([0,q]) such that $x(\hat{q})=1$ for some $\hat{q}<1$, define the functions

1.19 $$\begin{aligned} \begin{aligned} \Phi _{\nu }(t)&=\beta ^2 \xi '(t)-\int _0^t\frac{ds}{(\int ^1_s x(r)dr)^2},\\ \phi _{\nu }(s)&=\int ^s_0\Phi _{\nu }(t)dt. \end{aligned} \end{aligned}$$

Talagrand [45, Proposition 2.1] proved that the Parisi measure $\nu _{\beta ,P}$ is the unique measure such that

1.20 $$\begin{aligned} \text{ supp }(\nu )\subset \mathcal {S}_{\nu }:=\Big \{ s\in [0,1]: \phi _{\nu }(s)=\sup _{t\in [0,1]}\phi _{\nu }(t) \Big \}. \end{aligned}$$

For the Parisi measure, we denote $\mathcal {S}_{P}:=\mathcal {S}_{\nu _{P}}$ and by $q_P:=q_P(\beta )=\max \text {supp}(\nu _{P})<1$ the rightmost point in its support. Note that, as can be verified from the Parisi formula, $q_P>0$ if and only if $\beta >\beta _{c}$. Recall that a model $\xi (\cdot )$ is *k*-rsb at β for some k≥1, if the Parisi measure $\nu _P=\nu _{\beta ,P}$ is supported on 0 and *k* distinct positive points $q_i$. Generalizing [30, Definition 4] (beyond k=1 and zero-temperature), we say that such $\xi (\cdot )$ is *strictly* *k*-rsb at β if there exist $0=q_0< q_1<\cdots <q_k=q_P$ such that

1.21 $$\begin{aligned} \mathcal {S}_P\cap [0,q_P] = \text{ supp }(\nu _P)=\{q_0,\ldots ,q_k\} \end{aligned}$$

(while we do not rely on it, we note in passing that for generic mixtures $\mathcal {S}_P$ is the set of all multi-samplable overlaps, as shown in [42, Theorem 10]).

The problem of computing the Parisi measure, and in particular identifying whether it is *k*-rsb, has been studied in [7, 33, 48]. If $$\xi (\cdot )$$ is known to be *k*-rsb, one can easily check whether it is also strictly *k*-rsb by using (1.21). Generically, one expects most *k*-rsb models to be strict, but at phase transitions we can find *k*-rsb models which are not strictly *k*-rsb. For example, if $\xi^{\prime \prime }\left(0\right)=0$ then at the critical $$\beta _c$$ the maximal $$q \in \mathcal {S}_P$$ is strictly positive while $$\nu _P=\delta _0$$; see [42, Remark 13] for a proof of this fact and a related discussion on the special case of the 2-spin model.

Assume henceforth that our mixture ξ(t) is *k*-rsb for some finite *k*. Denoting the Euclidean gradient of $H_{N}(\textbf{x})$ in $\mathbb {R}^{N}$ by $\nabla H_{N}(\textbf{x})$, we define directional derivatives by

$$ \partial _{\textbf{u}} H_{N}(\textbf{x}):=\left\langle \nabla H_{N}(\textbf{x}),\textbf{u}\right\rangle \,, $$

and with an arbitrary matrix $M_i=M_i(\vec {\textbf{x}})\in \mathbb {R}^{(N-i)\times N}$ whose rows form an orthonormal basis of $\big (\textrm{sp}\{\textbf{x}_1,\ldots ,\textbf{x}_i\}\big )^{\perp }$, we define $\bar{\nabla }H_{N}(\textbf{x}_i):=M_{i}\nabla H_{N}(\textbf{x}_i)$. Note that $\bar{\nabla }H_{N}(\textbf{x}_i)$ depends also on the points $\textbf{x}_1,\ldots ,\textbf{x}_{i-1}$ (whose choice will be clear from the context). In particular, denoting hereafter $a\wedge b=\min \{a,b\}$ and $\{\textbf{e}_i\}$ the standard basis of $\mathbb {R}^N$, in Theorem 1.4 we use the vectors $\vec {\textbf{x}}^\textbf{e}_k=(\textbf{x}_1^{\textbf{e}},\ldots ,\textbf{x}_k^{\textbf{e}})$ where $\textbf{x}^{\textbf{e}}_i:= \sum _{j=1}^i \sqrt{N(q_j-q_{j-1})} \textbf{e}_j$ are such that $\frac{1}{N}\langle \textbf{x}_i^{\textbf{e}},\textbf{x}_j^{\textbf{e}} \rangle =q_{i\wedge j}$.

Given two vectors $\vec {E}=(E_i)_{i=1}^k$ and $\vec {R}=(R_i)_{i=1}^k$ of real numbers, another real number *E*, a collection of points $\vec {\textbf{x}}=(\textbf{x}_i)_{i=1}^k$ in $\mathbb {R}^N$ such that^3^$\frac{1}{N}\langle \textbf{x}_i,\textbf{x}_j\rangle =q_{i\wedge j}$ and a point $\boldsymbol{\sigma }\in \mathbb {S}^{N-1}$, we set $\textbf{x}_0=\textbf{0}$ and let $\mathrm{\textsf {CP}}(\boldsymbol{\sigma },\vec {\textbf{x}},E,\vec {E},\vec {R})$ be the event that

1.22 $$\begin{aligned} \forall i\le k :\quad \Big (\frac{1}{N}H_{N}(\boldsymbol{\sigma }),\,\frac{1}{N}H_{N}(\textbf{x}_i),\,\frac{\partial _{\textbf{x}_i-\textbf{x}_{i-1}}H_{N}(\textbf{x}_i)}{\Vert \textbf{x}_i-\textbf{x}_{i-1}\Vert ^2},\,\bar{\nabla }H_{N}(\textbf{x}_i)\Big )=\big (E,E_i,R_i,\textbf{0}\big ). \end{aligned}$$

With $\mathbb {B}^{N}\subset \mathbb {R}^N$ denoting the ball of radius $\sqrt{N r}$ for some $1 \le r < 1 + \varepsilon _\xi$ and $C^\infty (\mathbb {B}^{N})$ the class of real smooth functions on it, the statistics we wish to study under the Gibbs measure are of the form $f_N(\boldsymbol{\sigma })=\bar{f}_N(\boldsymbol{\sigma },H_N(\cdot ))$ for some deterministic $\bar{f}_N(\boldsymbol{\sigma },\varphi ):\mathbb {S}^{N-1}\times C^\infty (\mathbb {B}^{N})\rightarrow \mathbb {R}$ which is rotationally invariant. That is, $\bar{f}_N(\boldsymbol{\sigma },\varphi (\cdot )) = \bar{f}_N(O\boldsymbol{\sigma },\varphi (O^{T}\cdot ))$ for any orthogonal *O*. We further assume that for any $\boldsymbol{\sigma }\in \mathbb {S}^{N-1}$, $\varphi ,\psi _i\in C^\infty (\mathbb {B}^{N})$ and k≥1,

1.23 $$\begin{aligned} \lim _{t_{i}\rightarrow 0}\bar{f}_{N}\Big (\boldsymbol{\sigma },\varphi +\sum _{i\le k}t_{i}\psi _{i}\Big )=\bar{f}_{N}(\boldsymbol{\sigma },\varphi ). \end{aligned}$$

We note in passing that $\partial _{\textbf{x}_i-\textbf{x}_{i-1}}H_{N}(\textbf{x}_i)$ is measurable wrt $H_{N}(\textbf{x}_i)$ iff i=1 and the model is pure *p*-spin for some *p* (see the covariance calculations in [13, Appendix A]), in which case we omit the condition $\frac{\partial _{\textbf{x}_1}H_{N}(\textbf{x}_1)}{\Vert \textbf{x}_1\Vert ^2}=R_1$ from the event $\mathrm{\textsf {CP}}(\boldsymbol{\sigma },\vec {\textbf{x}},E,\vec {E},\vec {R})$. Henceforth, wlog the Gaussian vector which determines the event $\mathrm{\textsf {CP}}(\boldsymbol{\sigma },\vec {\textbf{x}}^\textbf{e}_k,E,\vec {E},\vec {R})$ has a strictly positive density. We thus define, analogously to (1.9), the conditional probability

$$ p_N(\boldsymbol{\sigma },(E,\vec {E},\vec {R})) := \mathbb {P}\big (\, f_{N}(\boldsymbol{\sigma })\in A\,\Big |\,\mathrm{\textsf {CP}}(\boldsymbol{\sigma },\vec {\textbf{x}}_k^{\textbf{e}},E,\vec {E},\vec {R})\,\big ) \,, $$

as the density with respect to the underlying Lebesgue measure of the corresponding sub-probability measure, normalized by the relevant Gaussian vector density at $(E,\vec {E},\vec {R},\textbf{0})$ and in particular to consider the (essential) supremum of $p_N(\cdot ,\cdot )$ over

1.24 $$\begin{aligned} B(\vec {\textbf{x}},\delta )&:=\left\{ \boldsymbol{\sigma }\in \mathbb {S}^{N-1}:\,|\langle \boldsymbol{\sigma },\textbf{x}_i\rangle -\langle \textbf{x}_i,\textbf{x}_i\rangle |\le N\delta ,\,\;\; \forall 1\le i\le k \right\} \,, \end{aligned}$$

1.25 $$\begin{aligned} V(\varepsilon )&:=\left\{ (E,\vec {E},\vec {R}):\,|E-F'(\beta )|,\,| E_i-E_{\star }(q_i)|,\,|R_i-R_{\star }(q_i)|<\varepsilon ,\,\;\; \forall 1\le i\le k\right\} \,. \end{aligned}$$

#### Theorem 1.4

Consider the spherical mixed *p*-spin model with a generic mixture ξ(t) and $\beta >\beta _{c}$ at which ξ(t) is strictly *k*-rsb. Suppose that $f_N(\boldsymbol{\sigma })=\bar{f}_N(\boldsymbol{\sigma },H_N(\cdot ))$ for some deterministic rotationally invariant $\bar{f}_N(\boldsymbol{\sigma },\varphi )$ which satisfies the continuity property (1.23) and that $f_N(\boldsymbol{\sigma })$ is $\mathbb {P}$-a.s. a measurable function on $\mathbb {S}^{N-1}$. If for some Borel $A\subset \mathbb {R}$

1.26 $$\begin{aligned} \lim _{\varepsilon \rightarrow 0}\varlimsup _{N\rightarrow \infty }\mathop {\mathrm {ess\,sup}}\limits _{B(\vec {\textbf{x}}_k^{\textbf{e}},\varepsilon )\times V(\varepsilon )}\frac{1}{N}\log \mathbb {P}\big (\, f_{N}(\boldsymbol{\sigma })\in A\,\Big |\,\mathrm{\textsf {CP}}(\boldsymbol{\sigma },\vec {\textbf{x}}_k^{\textbf{e}},E,\vec {E},\vec {R})\,\big )<0 \end{aligned}$$

then

1.27 $$\begin{aligned} \lim _{N\rightarrow \infty }\mathbb {E}G_{N,\beta }\left( f_{N}(\boldsymbol{\sigma })\in A\right) =0. \end{aligned}$$

Note that $\vec {\textbf{x}}_k^{\textbf{e}}$, the nominal values of $(E,\vec {E},\vec {R})$ in (1.26), and the overlaps $\{ \langle \boldsymbol{\sigma },\textbf{x}^{\textbf{e}}_i\rangle , i \le k\}$ that determine the narrow band $B(\vec {\textbf{x}}_k^{\textbf{e}},\varepsilon )$ of (1.24), are explicit functions of the Parisi measure $\nu _{\beta ,P}$.

#### Remark 1.5

It is necessary to have an extra conditioning in Theorem 1.4, beyond conditioning only on $\frac{1}{N}H_N(\boldsymbol{\sigma })= E\approx F'(\beta )$, as in Theorem 1.2 for high temperature. For instance consider the observable $f_N(\boldsymbol{\sigma }) = \Vert M_0 \nabla H_{N}(\boldsymbol{\sigma })\Vert /\sqrt{N}$ for $M_0=\textbf{I}_N-\frac{1}{N}\boldsymbol{\sigma }\boldsymbol{\sigma }^T$ (projecting the gradient to the orthogonal space). Conditional only on $\frac{1}{N}H_N(\boldsymbol{\sigma })= E$, we have that $f_N(\boldsymbol{\sigma })\overset{\textrm{d}}{=} \sqrt{\xi '(1)} \Vert X\Vert /\sqrt{N}$ for standard Gaussian $X \in \mathbb {R}^{N-1}$, hence $\mathbb {P}( |f_N(\boldsymbol{\sigma }) - \sqrt{\xi '(1)}| > \delta | \frac{1}{N}H_N(\boldsymbol{\sigma })= E) \le e^{-C_\delta N}$. However, we claim that for any δ>0 and all sufficiently large β,

1.28 $$\begin{aligned} \lim _{N\rightarrow \infty }\mathbb {E}G_{N,\beta }\left( |f_N(\boldsymbol{\sigma })| < \delta \right) =1\,. \end{aligned}$$

Indeed, by [34, Lemma C.3], for some $C,C^{\prime }>0$ depending only on ξ

$$ \mathbb {P}\Big (\sup _{\Vert \textbf{x}\Vert \le \sqrt{N}}\Vert \nabla ^2 H_N(\textbf{x})\Vert _\textrm{op}\ge C\Big )\le e^{-C'N}\,, $$

lower bounding the contribution to $Z_{N,\beta }$ from a small region around $\arg \max \{ H_N(\textbf{x}): \textbf{x}\in \mathbb {S}^{N-1}\}$ and thereby showing that $F(\beta )/\beta \rightarrow E_{\star }(1)$ as $\beta \rightarrow \infty$. This in turn implies that $F'(\beta )\rightarrow E_{\star }(1)$ (by convexity of F(β)). Recall [5] that for a sample $\boldsymbol{\sigma }$ from the Gibbs measure, $H_N(\boldsymbol{\sigma })/N \rightarrow F'(\beta )$ in probability, as $N \rightarrow \infty$. Hence, if (1.28) fails, then for some ε>0 and a subsequence in *N*,

$$ \mathbb {P}\big (\exists \textbf{x}\in \mathbb {S}^{N-1}: H_N(\textbf{x})/N>E_{\star }(1)+\varepsilon \big )\ge \varepsilon $$

in violation of the Borell-TIS inequality. Moreover, let $M_k\in \mathbb {R}^{(N-k-1)\times N}$ be a matrix whose rows form an orthonormal basis of $\big ( \textrm{sp}\{\boldsymbol{\sigma },\vec {\textbf{x}}_k^{\textbf{e}} \} \big )^{\perp }$. Conditioning only on $\frac{1}{N}H_N(\boldsymbol{\sigma })= E\approx F'(\beta )$ the observable $f_N(\boldsymbol{\sigma },\vec {\textbf{x}}_k^{\textbf{e}})= \Vert M_k\nabla H_{N}(\boldsymbol{\sigma })\Vert /\sqrt{N}$ concentrates around $\sqrt{\xi '(1)}$, while an application of Lemma 7.3 shows that it concentrates around $\sqrt{\xi '(1)-\xi '(q_k)}<\sqrt{\xi '(1)}$ when conditioning on $\mathrm{\textsf {CP}}(\boldsymbol{\sigma },\vec {\textbf{x}}_k^{\textbf{e}},E,\vec {E},\vec {R})$. In particular, in the context of Corollary A.1 that generalizes Theorem 1.4, the extra conditioning is essential for this observable, at all $\beta >\beta _c$.

#### Remark 1.6

The conclusion (1.27) provides an *o*(1) bound, weaker than the exponential decay (1.11) in the high-temperature case. The reason is that we rely on the pure state decomposition [31, 46] that controls the Gibbs measure up only over a set of measure 1-o(1). Strengthening the latter decomposition to cover $1-e^{-\Theta (N)}$ of the measure would result in a similar strengthening for (1.27), by a straightforward modification of our proof.

#### Remark 1.7

The pure *p*-spin models with mixture $\xi (t)=t^p$, p≥3 are strictly 1-rsb whenever $\beta >\beta _c$. While they are not generic, one can derive Theorem 1.4 for such pure models at all large enough β. Indeed, genericity is used in the proof of the theorem mainly to invoke the pure states decomposition of Proposition 3.1, but for the pure models such a decomposition follows at large β from the decomposition of [40]. In the generic case the collection of Gaussian random variables in (1.22) is non-degenerate (namely, they are not linearly dependent). In contrast, for pure models the radial derivative $\partial _{\textbf{x}_1}H_{N}(\textbf{x}_1)/\Vert \textbf{x}_1\Vert ^2$ is measurable wrt $H_{N}(\textbf{x}_1)$ and, as noted after (1.23), we omit the condition $\partial _{\textbf{x}_1}H_{N}(\textbf{x}_1)/\Vert \textbf{x}_1\Vert ^2=R_1$ from the definition of $\mathrm{\textsf {CP}}(\boldsymbol{\sigma },\vec {\textbf{x}}^\textbf{e}_1,E,\vec {E},\vec {R})$. This entails several technical modifications to the proof of Theorem 1.4, which are straightforward and left to the interested reader. In Theorem 1.8 below, we only state the analogous result for the pure *p*-spin models, denoting the corresponding event by $\mathrm{\textsf {CP}}(\boldsymbol{\sigma },\vec {\textbf{x}}_1^{\textbf{e}},E,\vec {E})$. Specifically, in the pure case, since k=1 the required complexity computations as in Section 6 can be taken directly from [4], the multi-level (m≥1 or n≥2) Kac-Rice formulas of Section 7 are not required, while the conditional laws of Lemma 7.3 are required only for m=1 and thus derived by a trivial modification of the proof for the generic case.

#### Theorem 1.8

Consider the spherical pure *p*-spin model with mixture $\xi (t)=t^p$ and suppose that $f_N(\boldsymbol{\sigma })=\bar{f}_N(\boldsymbol{\sigma },H_N(\cdot ))$ is as in Theorem 1.4. For any p≥3 there exists $\beta _p$ such that if $\beta >\beta _p$ and for some Borel $A\subset \mathbb {R}$

$$\begin{aligned} \lim _{\varepsilon \rightarrow 0}\varlimsup _{N\rightarrow \infty }\mathop {\mathrm {ess\,sup}}\limits _{B(\vec {\textbf{x}}_1^{\textbf{e}},\varepsilon )\times V(\varepsilon )}\frac{1}{N}\log \mathbb {P}\big (\, f_{N}(\boldsymbol{\sigma })\in A\,\Big |\,\mathrm{\textsf {CP}}(\boldsymbol{\sigma },\vec {\textbf{x}}_1^{\textbf{e}},E,\vec {E})\,\big )<0\,, \end{aligned}$$

then

$$\begin{aligned} \lim _{N\rightarrow \infty }\mathbb {E}G_{N,\beta }\left( f_{N}(\boldsymbol{\sigma })\in A\right) =0. \end{aligned}$$

In Section 2 we illustrate the wide applicability of our main results in the context of spherical mixed *p*-spin models, by considering Langevin dynamics initialized according to a Gibbs measure, and by computing the Franz-Parisi potential [27], including at the challenging low-temperature phase. In Section 3 we state the pure states decomposition of [31, 46] and our Proposition 3.1, by which its vertices can be chosen to be critical points (on appropriate spheres). In Section 5 we outline the proof of Theorem 1.4 about the low-temperature phase of spherical spin glasses. Section 4 is devoted to the proof of Theorem 1.2 about the high temperature phase. In Section 6 we collect several results from [13, 30, 42] about the complexity of critical points and prove some consequences of theirs. In Section 7 we use the multi-level Kac-Rice formula to express averages over critical points by certain complexities and the corresponding conditional expectations and probabilities given $\mathrm{\textsf {CP}}(\boldsymbol{\sigma },\vec {\textbf{x}}_n^{\textbf{e}},E,\vec {E},\vec {R})$. In Section 8 we prove the aforementioned Proposition 3.1 on the pure states decomposition. Finally, in Section 9 we combine the results of Sections 3, 6 and 7 to prove Theorem 1.4 about the low temperature phase and in Appendix A extend it to get Corollary A.1 (which we use in our study of Langevin dynamics).

## Applications

Section 2.1 shows how Theorem 1.2 and Corollary A.1 are utilized to convert the thermodynamic limits of Langevin dynamic (1.5) initialized according to a Gibbs measure, into such limits for non-random initial conditions with a suitably modified Gaussian disorder, while in Section 2.2 we apply Theorems 1.2 and 1.4, to compute the Franz-Parisi potential at any temperature.

### Langevin dynamics

For mixture ξ(t) of (1.13) such that $\xi (1+\varepsilon )<\infty$, consider the Langevin dynamic (1.5) at some $\widetilde{\beta }>0$, for t∈[0,T] and $\textbf{x}_0=\boldsymbol{\sigma }$ drawn from $G_{N,\beta }$ for some β>0. As shown in [24, Corollary 4.6], having $1<\rho <1+\varepsilon$ with $b_\ell '(\cdot )$ locally Lipschitz on [0,ρ) and $(\rho - r) b_\ell '(r) \rightarrow \infty$ as $r \uparrow \rho$, guarantees the existence of unique strong solutions of (1.5) in $C(\mathbb {R}^+,\mathbb {R}^N)$, with $C_N(t,t) < \rho$. Fixing hereafter such $b_\ell (\cdot )$ and $\xi (\cdot )$, our approach applies for any generic mixture and $\beta >\beta _c$ at which ξ(t) is strictly *k*-rsb, but for clarity we restrict hereafter to k=1, in which case we amend $(C_N,\chi _N,\widetilde{H}_N)$ of (1.6) by

2.1 $$\begin{aligned} q_N(s):=\frac{1}{N} \langle \textbf{x}_s, \textbf{x}_\star \rangle \,, \end{aligned}$$

for some $\textbf{x}_\star \in \sqrt{q_1} \mathbb {S}^{N-1}$ chosen in the sequel (in the *k*-rsb case one should amend $(C_N,\chi _N,\widetilde{H}_N)$ by *k* such functions). Also, aiming at a more uniform presentation, we set $\textbf{x}_\star :=\textbf{0}$ whenever $\beta \le \beta _c$. Then, denoting by $\Vert U_N\Vert _T$ the sup-norm of a generic function $U_N: [0,T]^2 \rightarrow \mathbb {R}$, for *N*, *T*, $H_N(\cdot )$ and a strong solution of (1.5) starting at $\textbf{x}_0=\boldsymbol{\sigma }\in \mathbb {S}^{N-1}$, we set the error between $\textbf{U}_N:=(C_N,\chi _N, \widetilde{H}_N,q_N)$ and a proposed non-random limit $\textbf{U}_\infty =(C,\chi ,H,q)$, to be

$$\begin{aligned} \bar{f}_{N} (\boldsymbol{\sigma },\textbf{x}_\star ,H_N(\cdot ) )&:= \mathbb {E}\Big [ \Vert C_N-C\Vert _T \wedge 1 +\Vert \chi _N-\chi \Vert _T \wedge 1 \\&+\Vert \widetilde{H}_N - H\Vert _T \wedge 1 + \Vert q_N-q\Vert _T \wedge 1 \Big ] \,, \end{aligned}$$

where the expectation is only over the Brownian motion $\textbf{B}_t$ (and the induced solution of (1.5)). Setting $A=(\delta ,\infty )$ we utilize Theorem 1.2 when $\beta \le \beta _c$ and Corollary A.1 when $\beta >\beta _c$, to conclude in [24, Theorem 1.8] that $\bar{f}_N(\boldsymbol{\sigma },\textbf{x}_\star ,H_N(\cdot )) \rightarrow 0$ in probability, for some explicit $\textbf{U}_\infty$ which is given in [24, Definitions 1.5 & 1.1], respectively.

Indeed, for $\beta \le \beta _c$ the high-temperature condition (1.8) holds, with $p_N(\boldsymbol{\sigma },E)$ of (1.9) measurable on $\mathbb {S}^{N-1}\times \mathbb {R}$ and the exponential convergence condition (1.12) is established in [24, (2.3)].

Turning to $\beta >\beta _c$, as noted in [24, (1.26)] the functional $\bar{f}_N(\boldsymbol{\sigma },\textbf{x}_\star ,\varphi _N(\cdot ))$ is rotationally invariant, its continuity in $\varphi _N$ is established in [24, Lemma 2.4] and its continuity in $\textbf{x}_\star$ is obvious. Further, for generic ξ(t) which is strictly 1-rsb at $\beta >\beta _c$ we have the pure state decomposition $(\textbf{x}^\star _i,B_i)_{i \le d_N}$ of Proposition 3.1. Fixing non-random $\textbf{x}^\star _0 \in \sqrt{q_1} \mathbb {S}^{N-1}$ and setting $B_0:= \mathbb {S}^{N-1}\setminus \cup _i B_i$, the partition $(B_i)_{i=0}^{d_N}$ induces the measurable mapping $\textbf{x}_\star (\boldsymbol{\sigma },H_N)=\textbf{x}^\star _i \in \sqrt{q_1} \mathbb {S}^{N-1}$. With $G_{N,\beta }(B_0) \rightarrow 0$, we have thanks to (3.4) and properties (2), (5) of Proposition 3.1 that with probability approaching one $\boldsymbol{\sigma }\in B(\textbf{x}_\star ,\varepsilon _N)$ and $\textbf{x}_\star \in \sqrt{q_1}\mathbb {S}^{N-1}$ such that $\bar{\nabla }H_{N}(\textbf{x}_\star )=0$ and

$$ \Big (\frac{1}{N}H_{N}(\boldsymbol{\sigma }),\,\frac{1}{N}H_{N}(\textbf{x}_\star ),\,\frac{\partial _{\textbf{x}_\star }H_{N}(\textbf{x}_\star )}{Nq_1} \, \Big )\in V(\varepsilon _{N}) $$

for some $\varepsilon _{N} \rightarrow 0$, with the exponential convergence condition (A.2) provided yet again by [24, (2.3)].

The thermodynamic limits for $\textbf{U}_N$ are derived in [11] (for $\textbf{x}_0$ of iid components), and in [23] (with a conditional disorder, albeit not the one required here), only for finite mixtures and without an exponential in *N* convergence rate. Consequently, [24, Sections 4 & 5] must extend the proofs in [11, 23] to cover different affine conditioning on $H_N(\cdot )$, as well as handling generic (infinite) mixtures and producing exponential in *N* convergence rates. The non-random limit $\textbf{U}_\infty$ for Gibbsian initial conditions is novel, even wrt the physics literature. The derivation of [24, (2.3)] is therefore supplemented in [24, Sections 1.2, 3 & 6] by various properties of $\textbf{U}_\infty$ which are beyond our scope in this work.

### Computing the Franz-Parisi potential

The Franz-Parisi potential involves sampling a point $$\boldsymbol{\sigma }$$ from the Gibbs measure and calculating, at an exponential scale, the Gibbs probability of sampling another point $$\boldsymbol{\sigma }'$$ whose overlap with $$\boldsymbol{\sigma }$$ is approximately *r*, where $$\boldsymbol{\sigma }$$ and $$\boldsymbol{\sigma }'$$ are allowed to be sampled from the Gibbs measure at two different inverse-temperatures β and $\beta^{\prime }$ (see [27]). In doing so, there are two sources of randomness, the disorder $$H_N$$ (chosen by law $$\mathbb {P}$$) and $$\boldsymbol{\sigma }$$ which is chosen according to $$G_{N,\beta }$$, while the Gibbs probability to sample $$\boldsymbol{\sigma }'$$ with a given overlap with $$\boldsymbol{\sigma }$$ is determined once we fix $$H_N$$ and $$\boldsymbol{\sigma }$$ (i.e., it is measurable wrt them). One then aims to understand the typical behavior of this variable as a function of *r*, which can be interpreted as the typical rate function for the overlap $$\langle \boldsymbol{\sigma },\boldsymbol{\sigma }'\rangle /N$$ (for N≫1, see below). Specifically, the Franz-Parisi potential is the random variable

2.2 $$\begin{aligned} V_{N,\beta ,\beta '}(r):=f_{N,r}(\boldsymbol{\sigma })+\frac{1}{2}\log (1-r^2)-F_{N,\beta '}, \end{aligned}$$

with $\boldsymbol{\sigma }$ a random point sampled from $G_{N,\beta }$ and

2.3 $$\begin{aligned} f_{N,r}(\boldsymbol{\sigma }):=\frac{1}{N}\log \int _{S(\boldsymbol{\sigma },r)}e^{\beta ' H_N(\boldsymbol{\sigma }')}d\boldsymbol{\sigma }', \end{aligned}$$

where $d\boldsymbol{\sigma }'$ denotes integration wrt the uniform measure on

$$ S(\boldsymbol{\sigma },r):=\big \{\boldsymbol{\sigma }'\in \mathbb {S}^{N-1}:\,\langle \boldsymbol{\sigma }',\boldsymbol{\sigma }\rangle /N=r \big \}, $$

and the logarithmic term in (2.2) is the limit of $\frac{1}{N}\log$ of the Hausdorff measure of $S(\boldsymbol{\sigma },r)$. We note in passing that since $H_N(\cdot )$ is $O(\sqrt{N})$-Lipschitz with overwhelmingly high probability (see, e.g. [17, Lemma 6.1] or [13, Corollary C.2]), one also has that

2.4 $$\begin{aligned} V_{N,\beta ,\beta '}(r) = \frac{1}{N}\log G_{N,\beta '}\Big (\big \{\bar{\boldsymbol{\sigma }}:\, |\langle \bar{\boldsymbol{\sigma }},\boldsymbol{\sigma }\rangle /N -r|<\varepsilon \big \} \Big )+ o_{N,\varepsilon }(1), \end{aligned}$$

where $o_{N,\varepsilon }(1)$ denotes a random variable which is smaller than δ with probability at least $1-e^{-N\kappa }$ provided that $N>N_0$ and $\varepsilon <\varepsilon _0$, for some $\kappa =\kappa (\xi )>0$, $N_0=N_0(\xi ,\delta )$ and $\varepsilon _0=\varepsilon _0(\xi ,\delta )$, uniformly in $r\in [-r_0,r_0]$ for any fixed $r_0\in (0,1)$. To see that, let $P_\perp$ be the projection to the orthogonal space to $\boldsymbol{\sigma }$ and note if we project any point $\bar{\boldsymbol{\sigma }}$ from the set in (2.4) to

2.5 $$\begin{aligned} \boldsymbol{\sigma }'=\sqrt{N(1-r^2)}P_\perp (\bar{\boldsymbol{\sigma }})/\Vert P_\perp (\bar{\boldsymbol{\sigma }})\Vert +r\boldsymbol{\sigma }\in S(\boldsymbol{\sigma },r), \end{aligned}$$

the distance $\Vert \boldsymbol{\sigma }'-\bar{\boldsymbol{\sigma }}\Vert$ is bounded by $\varepsilon \sqrt{N}C(r_0)$ for a constant $C(r_0)$ depending on $r_0$, assuming that $\varepsilon <(1-r_0)/2$. Using this and the co-area formula, the Gibbs probability can be related to $V_{N,\beta ,\beta '}(r)$ to prove (2.4).

**I. High temperature phase** We start with the analysis in the easier case of $\beta \le \beta _c$. Define the mixture

2.6 $$\begin{aligned} \tilde{\xi }_q(t):=\xi (q+(1-q)t)-\xi (q) \end{aligned}$$

and denote by $\tilde{F}(\beta ,r)$ the limiting free energy of the spherical model with mixture $\tilde{\xi }_{r^2}(t)$ at inverse-temperature β, as defined in (1.4).

#### Corollary 2.1

For any spherical model $H_N(\boldsymbol{\sigma })$, $\beta \le \beta _c$, |r|<1, $\beta ', \delta >0$ and $f_{N,r}(\boldsymbol{\sigma })$ as in (2.3),

$$ \varlimsup _{N\rightarrow \infty }\frac{1}{N}\log \mathbb {E}G_{N,\beta }\Big ( \big | f_{N,r}(\boldsymbol{\sigma })- \beta \beta '\frac{\xi (r)}{\xi (1)}-\tilde{F}(\beta ',r) \big | > \delta \Big )<0. $$

A similar result to Corollary 2.1 was recently proved in [25] for the spherical models and [6] for Ising models, both using a technique from [1] (while stated there only for $\beta =\beta '$ and, in [25], for pure models, their argument works similarly in the setting below).

#### Proof

As $F_{N,\beta }$ concentrates around its mean, which is given by the Parisi formula, for understanding $V_{N,\beta ,\beta '}(r)$ it suffices to analyze $f_{N,r}(\boldsymbol{\sigma })$. Further, since $S(\boldsymbol{\sigma },r)$ is a sphere of co-dimension 1, we can think of the restriction of $H_N(\boldsymbol{\sigma }')$ to it, as a new spherical Hamiltonian. More precisely, by the well-known formulas for the conditional law of jointly Gaussian variables one can easily check that, conditionally on $\frac{1}{N}H_{N}(\boldsymbol{\sigma })=E$, for $\boldsymbol{\sigma }' \in S(\boldsymbol{\sigma },r)$,

$$ H_N(\boldsymbol{\sigma }') = \frac{\xi (r)}{\xi (1)}NE+\tilde{H}_N(\boldsymbol{\sigma }'), $$

where $\tilde{H}_N(\boldsymbol{\sigma }')$ is a centered Gaussian process with covariance

2.7 $$\begin{aligned} \mathbb {E}\big [ \tilde{H}_N(\boldsymbol{\sigma }_1')\tilde{H}_N(\boldsymbol{\sigma }_2') \big ]&= N\xi \Big ({\textstyle \frac{1}{N}} \langle \boldsymbol{\sigma }_1', \boldsymbol{\sigma }_2' \rangle \Big ) -N\frac{\xi (r)^2}{\xi (1)} \nonumber \\&= N \tilde{\xi }_{r^2}\Big ( \frac{\langle \boldsymbol{\sigma }_1'-r\boldsymbol{\sigma }, \boldsymbol{\sigma }_2'-r\boldsymbol{\sigma }\rangle }{\Vert \boldsymbol{\sigma }_1'-r\boldsymbol{\sigma }\Vert \Vert \boldsymbol{\sigma }_2'-r\boldsymbol{\sigma }\Vert }\Big )+N\xi (r^2)-N\frac{\xi (r)^2}{\xi (1)}\,. \end{aligned}$$

Note that $\tilde{H}_N(\boldsymbol{\sigma }')$ has the same law as $\hat{H}_N(\boldsymbol{\sigma }')+\sqrt{N} X$, for a centered Gaussian variable *X* of variance $\xi (r^2)-\xi (r)^2/\xi (1)\ge 0$ which is independent of the centered Gaussian process $\hat{H}_N(\boldsymbol{\sigma }')$ on $S(\boldsymbol{\sigma },r)$ whose covariance matches the left-most term in (2.7). In particular, $\tilde{H}_N(\boldsymbol{\sigma }')$ and $\hat{H}_N(\boldsymbol{\sigma }')$ have the same expected free energy. Further, by the preceding and the concentration of the free energy (see [36, Theorem 1.2]), for any δ>0,

$$\begin{aligned}&\varlimsup _{\varepsilon \rightarrow 0}\varlimsup _{N\rightarrow \infty }\frac{1}{N}\log \sup _{\boldsymbol{\sigma }\in \Sigma _{N},\,|E-\beta |<\varepsilon }\mathbb {P}\\&\quad \left( \big | f_{N,r}(\boldsymbol{\sigma })-\beta \beta '\frac{\xi (r)}{\xi (1)}-\tilde{F}(\beta ',r)\big | > \delta \,\Big |\,\frac{1}{N}H_{N}(\boldsymbol{\sigma })=E\right) <0. \end{aligned}$$

The corollary follows as a direct consequence of Theorem 1.2. □

**II. Low temperature phase** Here we treat $\beta >\beta _c$, for which to the best of our knowledge there is no rigorous computation of the Franz-Parisi potential, for any spherical model. To this end, note that $f_{N,r}(\boldsymbol{\sigma })$ of (2.3) is of the form $\bar{f}_N(\boldsymbol{\sigma },H_N(\cdot ))$ for a deterministic, rotationally invariant $\bar{f}_N$, that satisfies (1.23). We shall thus use Theorem 1.4 and work with the event $\mathrm{\textsf {CP}}(\boldsymbol{\sigma },\vec {\textbf{x}}^{\textbf{e}},E,\vec {E},\vec {R})$ of (1.22), while simplifying our presentation by assuming that $\xi (\cdot )$ is not pure. The straightforward adaptation to the pure case (and β large enough so that a pure states decomposition exists), by omitting the conditioning on $\partial _{\textbf{x}_1}H_{N}(\textbf{x}_1)$, is left to the reader (see Remark 1.7). We note in passing that in the final formula (2.16), this would amount to removing the 3rd column and row from the matrix $C(q_{\star })$ below, as well as removing the 3rd entry from the vectors ${\textsf {v}}$ and ${\textsf {u}}$. While for simplicity we consider only the case of strictly 1-rsb, the same principles apply, with obvious modifications, to the more involved case of strictly *k*-rsb for k≥2.

Proceeding to evaluate the value around which $f_{N,r}(\boldsymbol{\sigma })$ concentrates, set $\textbf{x}_1=\textbf{x}_1^{\textbf{e}}=(\sqrt{Nq_1},0,\ldots ,0)$, and fixing the point $\boldsymbol{\sigma }_1 =(\sqrt{N q_1},\sqrt{N(1-q_1)},0,\ldots ,0) \in B(\textbf{x}_1,0)$, for $|\rho | \le q_1$, let

$$ S(\boldsymbol{\sigma }_1,r;\textbf{x}_1,\rho ) := \big \{\boldsymbol{\sigma }'\in \mathbb {S}^{N-1}:\,\langle \boldsymbol{\sigma }',\boldsymbol{\sigma }_1 \rangle /N=r,\, \langle \boldsymbol{\sigma }',\textbf{x}_1 \rangle /N=\rho \big \}\,. $$

The set $S(\boldsymbol{\sigma }_1,r;\textbf{x}_1,\rho )$ is non-empty if and only if

2.8 $$\begin{aligned} \tau = \tau (q_1,r,\rho ):= \frac{\rho ^2}{q_1}+ \frac{(r-\rho )^2}{1- q_1} =r^2 +\frac{(\rho - r q_1)^2}{q_1-q_1^2} \le 1\,, \end{aligned}$$

in which case it is a sphere of radius $\sqrt{N(1-\tau )}$ in $\tilde{\boldsymbol{\sigma }}':= (\sigma '_3,\ldots ,\sigma '_N)$, centered at

$$ \boldsymbol{\sigma }_o:=\frac{r-\rho }{1- q_1}\boldsymbol{\sigma }_1 + \frac{\rho - rq_1}{q_1- q_1^2} \textbf{x}_1 \,. $$

Alternatively, $S(\boldsymbol{\sigma }_1,r;\textbf{x}_1,\rho )$ is non-empty over the interval

2.9 $$\begin{aligned} J=J(q_1,r):=\Big \{\rho :\,|\rho - rq_1|\le \sqrt{q_1-q_1^2}\sqrt{1-r^2}\,\Big \} \end{aligned}$$

and consequently

2.10 $$\begin{aligned} S(\boldsymbol{\sigma }_1,r) = S(\boldsymbol{\sigma }_1,r;\textbf{x}_1,J), \quad \text {for} \quad S(\boldsymbol{\sigma }_1,r;\textbf{x}_1,I) := \bigcup _{\rho \in I} S(\boldsymbol{\sigma }_1,r;\textbf{x}_1,\rho ). \end{aligned}$$

The event $\mathrm{\textsf {CP}}_1:=\mathrm{\textsf {CP}}(\boldsymbol{\sigma }_1,\textbf{x}_1,E, E_1, R_1)$ is the intersection of

2.11 $$\begin{aligned}&\Big (\frac{\partial }{\partial x_3}H_{N}(\textbf{x}_1),\ldots ,\frac{\partial }{\partial x_N}H_{N}(\textbf{x}_1)\Big )=0\,, \end{aligned}$$

2.12 $$\begin{aligned}&\bigg (\frac{1}{N} H_{N}(\boldsymbol{\sigma }_1),\,\frac{1}{N} H_{N}(\textbf{x}_1),\, \frac{1}{\sqrt{N q_1}} \frac{\partial }{\partial x_1} H_{N}(\textbf{x}_1),\, \frac{1}{\sqrt{N}} \frac{\partial }{\partial x_2} H_{N}(\textbf{x}_1)\bigg ) \!=\!\big (E,E_1, R_1,0\big ) \,. \end{aligned}$$

The covariances between these variables are easily computed (see the proof of Lemma 7.3 for a similar computation). Specifically, the covariance matrix of the vector in (2.11) is $\xi '(q_1) \textbf{I}_{N-2}$ (where $\textbf{I}_{N-2}$ denotes the identity matrix), while the vector in (2.12) has the covariance matrix $N^{-1} C(q_1)$, for

$$ C(q_1):= \begin{pmatrix} \xi (1) &  \xi (q_1) &  \xi '(q_1) &  \sqrt{1-q_1}\xi '(q_1)\\ \xi (q_1) &  \xi (q_1) &  \xi '(q_1) &  0\\ \xi '(q_1) &  \xi '(q_1) &  \xi ''(q_1)+\xi '(q_1)/q_1 &  0\\ \sqrt{1-q_1}\xi '(q_1) &  0 &  0 &  \xi '(q_1) \end{pmatrix}, $$

which is invertible (whenever ξ(t) is not pure).^4^ Further, for any $\boldsymbol{\sigma }'\in S(\boldsymbol{\sigma }_1,r;\textbf{x}_1,\rho )$, the covariance of $H_N(\boldsymbol{\sigma }')$ and the vector in (2.11) is $\xi '(\rho ) \tilde{\boldsymbol{\sigma }}'$, whereas that of $H_N(\boldsymbol{\sigma }')$ and the vector in (2.12) is then

$$ {\textsf {v}}={\textsf {v}}(q_1,r,\rho ):=\Big (\xi (r),\,\xi (\rho ),\,\xi '(\rho )\rho /q_1,\,\xi '(\rho )\frac{r-\rho }{\sqrt{1-q_1}}\Big )\,. $$

Since the vectors in (2.11) and in (2.12) are independent, we have that for any $\boldsymbol{\sigma }' \in S(\boldsymbol{\sigma }_1,r;\textbf{x}_1,\rho )$,

2.13 $$\begin{aligned} \frac{1}{N} \mathbb {E}[ H_N(\boldsymbol{\sigma }') \big | \, \mathrm{\textsf {CP}}_1 \, ] = {\textsf {v}}(q_1,r,\rho ) C(q_1)^{-1} \big (E,E_1, R_1,0\big )^{\top }. \end{aligned}$$

Further, conditionally on $\mathrm{\textsf {CP}}_1$, we have that $H_N(\cdot ) = \tilde{H}_N(\cdot ) + \mathbb {E}[H_{N}(\cdot )\,|\,\mathrm{\textsf {CP}}_1]$ for a centered Gaussian process $\tilde{H}_N(\cdot )$ such that for any $\boldsymbol{\sigma }'_1, \boldsymbol{\sigma }_2' \in S(\boldsymbol{\sigma }_1,r;\textbf{x}_1,\rho )$,

$$ \frac{1}{N}\mathbb {E}\big [ \tilde{H}_N(\boldsymbol{\sigma }'_1) \tilde{H}_N(\boldsymbol{\sigma }'_2) \big ] = \xi (\tfrac{1}{N}\langle \boldsymbol{\sigma }'_1,\boldsymbol{\sigma }'_2\rangle ) -\frac{\xi '(\rho )^2}{\xi '(q_1)}\frac{1}{N} \langle \tilde{\boldsymbol{\sigma }}'_1 ,\tilde{\boldsymbol{\sigma }}'_2 \rangle - {\textsf {v}}C(q_1)^{-1}{\textsf {v}}^{\top } \,. $$

Recall that then $\langle \boldsymbol{\sigma }'_1,\boldsymbol{\sigma }'_2\rangle =N \tau + \langle \tilde{\boldsymbol{\sigma }}'_1,\tilde{\boldsymbol{\sigma }}'_2 \rangle$ for $\tau =\tau (q_1,r,\rho )$ of (2.8), and consequently

2.14 $$\begin{aligned} \frac{1}{N}\mathbb {E}\big [ \tilde{H}_N(\boldsymbol{\sigma }'_1) \tilde{H}_N(\boldsymbol{\sigma }'_2) \big ]&= \xi _{q_1,\tau ,\rho }\Big ( \frac{\langle \tilde{\boldsymbol{\sigma }}'_1, \tilde{\boldsymbol{\sigma }}'_2 \rangle }{\Vert \tilde{\boldsymbol{\sigma }}'_1\Vert \Vert \tilde{\boldsymbol{\sigma }}'_2\Vert } \Big ) +\xi \left( \tau \right) - {\textsf {v}}C(q_1)^{-1}{\textsf {v}}^{\top }, \end{aligned}$$

so up to mapping $\boldsymbol{\sigma }\mapsto \tilde{\boldsymbol{\sigma }}$ and scaling, $\tilde{H}_N(\cdot )$ is the sum of the Hamiltonian for the mixture

2.15 $$\begin{aligned} \xi _{q_1,\tau ,\rho }(t) : = \xi \big (\tau +(1-\tau )t\big ) -\xi (\tau ) - \frac{\xi '(\rho )^2}{\xi '(q_1)}(1-\tau )t \end{aligned}$$

and an independent centered Gaussian variable of variance *NA*, where $A:=\xi \left( \tau \right) - {\textsf {v}}C(q_1)^{-1}{\textsf {v}}^{\top }$.

#### Remark 2.2

Indeed, $\xi _{q_1,\tau ,\rho }(t)=\sum _{p} (\xi ^{(p)}_{q_1,\tau ,\rho }(0)/p!) t^p$ is a mixture, as $\xi _{q_1,\tau ,\rho }(0)=0$ and $\xi ^{(p)}_{q_1,\tau ,\rho }(0) = (1-\tau )^p \xi ^{(p)}(\tau ) \ge 0$ for all p≥2, while with $\sqrt{\tau q_1}\ge \rho$ we have that

$$\begin{aligned} \xi '_{q_1,\tau ,\rho }(0)= (1-\tau )(\xi '(\tau )-\xi '(\rho )^2/\xi '(q_1)) \ge 0 \end{aligned}$$

(since $\xi '(\cdot ) \ge 0$ is non-decreasing and by Cauchy-Schwarz also $\xi '(a)\xi '(b)\ge \xi '(\sqrt{ab})^2$ for all a,b∈[0,1]). Moreover, A≥0 since there are N-2≫1 points $\textbf{x}_i$ such that the entries of the non-negative definite covariance matrix of $\{N^{-1/2} \tilde{H}_N(\textbf{x}_i)\}$ are $\xi _{q_1,\tau ,\rho }(1) \textbf{1}_{i=j}+A$.

#### Theorem 2.3

Consider the spherical mixed *p*-spin model with a generic mixture ξ(t), for $\beta >\beta _{c}$ at which ξ(t) is strictly 1-rsb. Let ${\textsf {u}}:= C(q_1)^{-1} (F'(\beta ),E_{\star }(q_1),R_{\star }(q_1),0)^{\top }$, $\tau =\tau (q_1,r,\rho )$ of (2.8) and $F(\beta ; q_1,\tau ,\rho )$ the limiting free energy, as in (1.4), for the spherical spin model that corresponds to $\xi _{q_1,\tau ,\rho }(\cdot )$ of (2.15). Then, for $f_{N,r}(\boldsymbol{\sigma })$ of (2.3) and any |r|<1, $\beta ',\delta >0$,

$$ \lim _{N\rightarrow \infty }\mathbb {E}G_{N,\beta }\big ( |f_{N,r}(\boldsymbol{\sigma })- \Lambda (\beta ,\beta ',r)|> \delta \big )=0, $$

for the limiting Franz-Parisi potential

2.16 $$\begin{aligned} \begin{aligned} \Lambda (\beta ,\beta ',r)&:= \sup _{\rho \in J(q_1,r)} \{ \lambda (\beta ,\beta ',r,\rho ) \} \,, \\ \lambda (\beta ,\beta ',r,\rho )&:= \beta ' \langle {\textsf {v}}(q_1,r,\rho ), {\textsf {u}}\rangle + F(\beta ' ; q_1,\tau ,\rho ) +\frac{1}{2} \log \Big (\frac{1-\tau (q_1,r,\rho )}{1-r^2}\Big ). \end{aligned} \end{aligned}$$

### Proof of Theorem 2.3

The following remark specifies our later use of Anderson’s inequality.

#### Remark 2.4

Anderson’s inequality [2], states that for any symmetric $f:\mathbb {R}^d\rightarrow [0,\infty )$ of

convex super-level sets, any symmetric, convex $K\subset \mathbb {R}^d$, all c∈[0,1] and $y\in \mathbb {R}^d$,

$$ \int _{K}f(x+cy)\,\textrm{d} x\ge \int _{K}f(x+y)\,\textrm{d} x \,. $$

As a consequence (stated as [2, Corollary 3]), if $X\sim \textsf {N}(0,\Sigma _1)$, $Y\sim \textsf {N}(0,\Sigma _2)$ with $\Sigma _1-\Sigma _2$ non-negative definite, then $\mathbb {P}(X\in K)\le \mathbb {P}(Y\in K)$ for any symmetric, convex *K*. Suppose that $(W(t))_{t\in T}$ is an a.s. continuous, centered Gaussian process on some closed $T\subset \mathbb {R}^n$, *U* is a Gaussian vector and W(·) and *U* are jointly Gaussian. Then, in distribution, $W(\cdot ) = \tilde{W}(\cdot ) + \mathbb {E}[W(\cdot )\,|\,U]$ with both summands independent and $\tilde{W}(\cdot )$ a centered Gaussian process such that $\mathbb {E}W(x) W(y) - \mathbb {E}\tilde{W}(x) \tilde{W}(y)$ is non-negative definite. Of course, $\tilde{W}(\cdot )$ and $\mathbb {E}[W(\cdot )\,|\,U]$ are a.s. continuous. Fixing a dense collection $\{x_i\} \subset T$ of distinct points, s≥0 and finite *I*, since the set $K= \{(s_i)_{i\in I}\in \mathbb {R}^I: \sup _{i\ne j} |s_i-s_j|/|x_i-x_j|\le s \}$ is convex and symmetric, we have that

$$\begin{aligned}&\mathbb {P}\Big ( \sup _{i\ne j\in I}\frac{|W(x_i)-W(x_j)|}{|x_i-x_j|}\le s \Big ) =\mathbb {P}\Big ( \big (W(x_i)\big )_{i\in I}\in K \Big )\\&\le \mathbb {P}\Big ( \big (\tilde{W}(x_i)\big )_{i\in I}\in K \Big )=\mathbb {P}\Big ( \sup _{i\ne j\in I}\frac{|\tilde{W}(x_i)-\tilde{W}(x_j)|}{|x_i-x_j|}\le s \Big ). \end{aligned}$$

Taking $$|I| \uparrow \infty $$

we see that the Lipschitz constant of $\tilde{W}(\cdot )$ is stochastically dominated by that of W(·).

Turning to prove Theorem 2.3, by Theorem 1.4, we only need to show that for any $\varepsilon _{N}\rightarrow 0$,

$$\begin{aligned}&\varlimsup _{N\rightarrow \infty }\sup _{B(\textbf{x}_1,\varepsilon _N)\times V(\varepsilon _N)}\frac{1}{N}\log \\&\quad \mathbb {P}\left( \,|f_{N,r}(\boldsymbol{\sigma })- \Lambda (\beta ,\beta ',r)|> \delta \,\Big |\,\mathrm{\textsf {CP}}(\boldsymbol{\sigma },\textbf{x}_1,E, E_1, R_1)\,\right) <0. \end{aligned}$$

Further, $f_{N,r}(\boldsymbol{\sigma })$ is a free energy of a model on $S(\boldsymbol{\sigma },r)$ whose conditional variance is bounded by the unconditional variance Nξ(1). Hence by concentration (see [36, Theorem 1.2]), it suffices to show that for any $\varepsilon _{N}\rightarrow 0$,

2.17 $$\begin{aligned} \varlimsup _{N\rightarrow \infty }\sup _{B(\textbf{x}_1,\varepsilon _N)\times V(\varepsilon _N)} \left| \mathbb {E}\big [\,f_{N,r}(\boldsymbol{\sigma })\,\big |\,\mathrm{\textsf {CP}}(\boldsymbol{\sigma },\textbf{x}_1,E, E_1, R_1)\,\big ] - \Lambda (\beta ,\beta ',r)\right| = 0. \end{aligned}$$

Since the law of $H_N(\boldsymbol{\sigma })$ is invariant under rotations of $\mathbb {R}^N$, the conditional expectation in (2.17) depends on $\boldsymbol{\sigma }$ only through $\langle \boldsymbol{\sigma },\textbf{x}_1\rangle /N$. Hence, it is enough to restrict the supremum there to $\boldsymbol{\sigma }=\boldsymbol{\sigma }(q):= (\sqrt{Nq},\sqrt{N(1-q)},0,\ldots ,0)$ for *q* such that $|\langle \boldsymbol{\sigma }(q),\textbf{x}_1\rangle -\langle \textbf{x}_1,\textbf{x}_1\rangle |/N=|\sqrt{qq_1}-q_1|\le \varepsilon _{N}$. We thus set analogously to (1.25),

$$\begin{aligned} \widetilde{V}(\varepsilon ) :\!=\! \left\{ (q,E,E_1,R_1):\,|q\!-\!q_1|,\,|E\!-\!F'(\beta )|,\,| E_1\!-\!E_{\star }(q_1)|,\,|R_1\!-\!R_{\star }(q_1)|<\varepsilon \right\} , \end{aligned}$$

and events $\mathrm{\textsf {CP}}_1^q:=\mathrm{\textsf {CP}}(\boldsymbol{\sigma }_1,O_q\textbf{x}_1,E, E_1, R_1)$, for $\mathrm{\textsf {CP}}(\cdot )$ of (1.22) and the orthogonal matrix $O_q\in \mathbb {R}^{N\times N}$ that maps $\boldsymbol{\sigma }(q)$ to $\boldsymbol{\sigma }_1=\boldsymbol{\sigma }(q_1)$ and acts as the identity on $\textrm{sp}\{\boldsymbol{\sigma }(q),\boldsymbol{\sigma }_1\}^{\perp }$. Now, by rotational invariance

$$ \mathbb {E}\big [\,f_{N,r}(\boldsymbol{\sigma }(q))\,\big |\,\mathrm{\textsf {CP}}(\boldsymbol{\sigma }(q),\textbf{x}_1,E, E_1, R_1)\,\big ] = \mathbb {E}\big [\,f_{N,r}(\boldsymbol{\sigma }_1)\,\big |\, \mathrm{\textsf {CP}}_1^q \,\big ] \,, $$

and with the nominal event $\mathrm{\textsf {CP}}_\star := \mathrm{\textsf {CP}}(\boldsymbol{\sigma }_1,\textbf{x}_1,F'(\beta ), E_{\star }(q_1), R_{\star }(q_1))$, we get (2.17) by showing that

2.18 $$\begin{aligned} \varlimsup _{N\rightarrow \infty }&\sup _{\widetilde{V}(\varepsilon _N)} \left| \mathbb {E}\big [\,f_{N,r}(\boldsymbol{\sigma }_1)\,\big |\,\mathrm{\textsf {CP}}_1^q \,\big ] - \mathbb {E}\big [\,f_{N,r}(\boldsymbol{\sigma }_1)\,\big |\,\mathrm{\textsf {CP}}_\star \,\big ] \right| = 0, \end{aligned}$$

2.19 $$\begin{aligned} \lim _{N\rightarrow \infty }&\mathbb {E}\big [\,f_{N,r}(\boldsymbol{\sigma }_1)\,\big |\,\mathrm{\textsf {CP}}_\star \,\big ] = \Lambda (\beta ,\beta ',r)\,. \end{aligned}$$

Turning to show (2.18), let

$$\begin{aligned} C_N&:= \beta ' \sup _{\widetilde{V}(\varepsilon _N)} \sup _{\boldsymbol{\sigma }_1'\in S(\boldsymbol{\sigma }_1,r)}\frac{1}{N}\left| \mathbb {E}\big [H_N(\boldsymbol{\sigma }_1')\,\big |\,\mathrm{\textsf {CP}}_1^q ] -\mathbb {E}\big [H_N(\boldsymbol{\sigma }_1')\,\big |\,\mathrm{\textsf {CP}}_\star ] \right| ,\\ D_N&:= \beta '^2 \sup _{\widetilde{V}(\varepsilon _N)} \sup _{\boldsymbol{\sigma }_1',\boldsymbol{\sigma }_2'\in S(\boldsymbol{\sigma }_1,r)}\frac{1}{N}\left| \text{ Cov }\big (H_N(\boldsymbol{\sigma }_1'),H_N(\boldsymbol{\sigma }_2')\,\big |\,\mathrm{\textsf {CP}}_1^q\big ) \right. \\&\left. -\text{ Cov }\big (H_N(\boldsymbol{\sigma }_1'),H_N(\boldsymbol{\sigma }_2')\,\big |\,\mathrm{\textsf {CP}}_\star \big ) \right| \end{aligned}$$

where $\text{ Cov }\big (\, \cdot ,\cdot \,\big |\,\mathrm{\textsf {CP}}_1^{q} \big )$ denotes the conditional covariance. Note that for all *N* the space on which the free energy of $H_N(\boldsymbol{\sigma }')$ is computed is $S(\boldsymbol{\sigma }_1,r)$ and by Gaussian interpolation (see [36, Section 1.2]),

$$\begin{aligned}&\left| \mathbb {E}\big [f_{N,r}(\boldsymbol{\sigma }_1)\,\big |\,\mathrm{\textsf {CP}}^q_1 ]-\mathbb {E}\big [f_{N,r}(\boldsymbol{\sigma }_1)\,\big |\,\mathrm{\textsf {CP}}_\star ] \right| \le C_N+D_N \,. \end{aligned}$$

Indeed, the term $C_N$ bounds the worst-case effect from the difference in the mean of the two conditional fields. Thereafter, let $\nu (\boldsymbol{\sigma }):= e^{\beta ' \mathbb {E}[H_N(\boldsymbol{\sigma })|\mathrm{\textsf {CP}}_\star ]}$ and consider the interpolation $g_N^t=\sqrt{t} g_N^1+\sqrt{1-t} g_N^0$ for independent, centered, Gaussian fields $g_N^0$, $g_N^1$ on $S(\boldsymbol{\sigma }_1,r)$, setting $\varphi (t)=\frac{1}{N}\mathbb {E}\log \int _{S(\boldsymbol{\sigma }_1,r)}e^{g_N^t(\boldsymbol{\sigma })}\nu (\boldsymbol{\sigma }) d\boldsymbol{\sigma }$. After Gaussian integration by parts, one has

$$ \varphi '(t)= \frac{1}{2} \mathbb {E}\langle C_1(\boldsymbol{\sigma }_1',\boldsymbol{\sigma }_1')-C_0(\boldsymbol{\sigma }_1',\boldsymbol{\sigma }_1')\rangle _t -\frac{1}{2} \mathbb {E}\langle C_1(\boldsymbol{\sigma }_1',\boldsymbol{\sigma }_2') - C_0(\boldsymbol{\sigma }_1',\boldsymbol{\sigma }_2') \rangle _t \,, $$

where $\langle \cdot \rangle _t$ denotes the product Gibbs averaging of $\boldsymbol{\sigma }_1',\boldsymbol{\sigma }_2'$ under Hamiltonian $g_N^t$ and $C_i(\boldsymbol{\sigma }_1',\boldsymbol{\sigma }_2'):=\frac{1}{N}\mathrm{ Cov } (g_N^i(\boldsymbol{\sigma }_1'),g_N^i(\boldsymbol{\sigma }_2'))$. Hence, $|\varphi (1)-\varphi (0)| \le \sup _{\boldsymbol{\sigma }_1',\boldsymbol{\sigma }_2'} |C_1(\boldsymbol{\sigma }_1',\boldsymbol{\sigma }_2') - C_0(\boldsymbol{\sigma }_1',\boldsymbol{\sigma }_2')|$, yielding in our case the term $D_N$ (as the worst-case over $\widetilde{V}(\varepsilon _N)$). We further claim that

2.20 $$\begin{aligned} \varlimsup _{N\rightarrow \infty } C_N=\varlimsup _{N\rightarrow \infty } D_N=0 \,. \end{aligned}$$

To see this, first note that setting $T:=\text{ span }(X)$ for $X=\{\boldsymbol{\sigma }_1',\boldsymbol{\sigma }_2',\boldsymbol{\sigma }_1,O_q\textbf{x}_1\}$, $\{\partial _{\textbf{x}}H_N(O_q\textbf{x}_1)\}_{\textbf{x}\in T^{\perp }}$ and $\{H_N(\textbf{y})\}_{\textbf{y}\in X}\cup \{\partial _{\textbf{x}}H_N(O_q\textbf{x}_1)\}_{\textbf{x}\in T}$ are independent of each other. Hence, letting $Y=\{\textbf{y}_1,\textbf{y}_2,\textbf{y}_3\}$ be unit vectors such that $\text{ span }(Y)=\{\textbf{z}\in T:\,\textbf{z}\perp O_q\textbf{x}_1\}$, the conditional law of $H_N(\boldsymbol{\sigma }_1')$ and $H_N(\boldsymbol{\sigma }_2')$ given $\mathrm{\textsf {CP}}_1^q$ is the same as their conditional law given that

$$\begin{aligned}&\Big (\frac{1}{N}H_{N}(\boldsymbol{\sigma }_1),\,\frac{1}{N}H_{N}(O_q \textbf{x}_1),\,\frac{\partial _{O_q\textbf{x}_1}H_{N}(O_q\textbf{x}_1)}{\Vert O_q\textbf{x}_1\Vert ^2},\,(\partial _{\textbf{y}_j} H_{N}(O_q\textbf{x}_1))_{j\le 3}\Big )\\&\quad =\big (E,E_1,R_1, \textbf{0}\big ). \end{aligned}$$

The conditional mean and covariance as in the definition of $$C_N$$ and $$D_N$$ above therefore depend continuously on $$(E,E_1,R_1)$$ and the values ξ, $\xi^{\prime }$ and $\xi^{\prime \prime }$ at the normalized inner products $$\langle \textbf{x},\textbf{y}\rangle /N$$ for $$\textbf{x},\textbf{y}\in X\cup Y$$, uniformly on $$\widetilde{V}(\varepsilon )$$ for any small ε (in a manner which is independent of *N*; see also the more involved setting, with k≥1, in our proof of Lemma 9.1). From this, (2.20) easily follows.

Moving next to (2.19), we fix k≥1 and partition the interval *J* of (2.9) to *k* equal sub-intervals $J_i$, writing in view of (2.10),

$$\begin{aligned}&f_{N,r}(\boldsymbol{\sigma }_1) = \frac{1}{N}\log \Big ( \sum _{i=1}^{k} e^{N f_{N,r}(\boldsymbol{\sigma }_1;J_i)} \Big ) \quad \text {for} \quad f_{N,r}(\boldsymbol{\sigma }_1;J_i)\\&\quad := \frac{1}{N} \log \Big ( \int _{S(\boldsymbol{\sigma }_1,r;\textbf{x}_1,J_i)}\!\!\!\!\!\!\!\!\!\!\!\! e^{\beta ' H_N(\boldsymbol{\sigma }')}d\boldsymbol{\sigma }' \Big ). \end{aligned}$$

Each $f_{N,r}(\boldsymbol{\sigma }_1;J_i)$ is a free energy of a Hamiltonian whose conditional variance given $\mathrm{\textsf {CP}}_\star$ is bounded everywhere by the unconditional variance Nξ(1). Hence, from concentration (see [36, Theorem 1.2]),

$$ \mathbb {E}\big [\,f_{N,r}(\boldsymbol{\sigma }_1)\,\big |\,\mathrm{\textsf {CP}}_\star \,\big ] = \max _{i=1}^k \mathbb {E}\Big [\, f_{N,r}(\boldsymbol{\sigma }_1;J_i) \,\Big |\,\mathrm{\textsf {CP}}_\star \,\Big ] + o_N(1)\,. $$

In addition, there exist δ>0 and closed interval $J' \subset J^o$ (the interior of *J*) such that for all large *N*,

$$ \max _{J_i \subset J \setminus J'} \mathbb {E}\Big [\, f_{N,r}(\boldsymbol{\sigma }_1;J_i) \,\Big |\,\mathrm{\textsf {CP}}_\star \,\Big ] \le \mathbb {E}\Big [\, f_{N,r}(\boldsymbol{\sigma }_1;J \setminus J')\,\Big |\,\mathrm{\textsf {CP}}_\star \,\Big ] \le \Lambda (\beta ,\beta ',r) - \delta $$

(since decreasing $J\backslash J^{\prime }$ makes $$\frac{1}{N}\log (\mathrm{ Vol }(S(\boldsymbol{\sigma }_1,r;\textbf{x}_1,J \setminus J')))$$ as negative as we wish).

To complete the proof of (2.19), it thus suffices to show that for $\lambda (\beta ,\beta ',r,\rho )$ of (2.16), some C<∞, all *k* large and any *i* such that $J_i \not \subset J\setminus J'$

2.21 $$\begin{aligned} \varlimsup _{N\rightarrow \infty } \sup _{\rho \in J_i} \Big | \mathbb {E}\Big [\, f_{N,r}(\boldsymbol{\sigma }_1;J_i) \,\Big |\,\mathrm{\textsf {CP}}_\star \,\Big ] - \lambda (\beta ,\beta ',r,\rho ) \Big | \le C/k \,. \end{aligned}$$

To establish (2.21), note that by the co-area formula

$$ f_{N,r}(\boldsymbol{\sigma }_1;J_i) =\frac{1}{N} \log \Big ( \int _{J_i} \text{ Jac }(s) \int _{S(\boldsymbol{\sigma }_1,r;\textbf{x}_1,s)} \!\!\!\!\!\!\!\!\!\!\!\!\!\!\!\! e^{\beta ' H_N(\boldsymbol{\sigma }')}d\boldsymbol{\sigma }' ds\Big ) , $$

where the inner integration is wrt the uniform measure on $S(\boldsymbol{\sigma }_1,r;\textbf{x}_1,s)$ and for τ as in (2.8),

$$ \text{ Jac }(s) \propto \Big (\frac{1-\tau (q_1,r,s)}{1-r^2}\Big )^{\frac{N-4}{2}}\qquad \text{ and }\qquad \ \ \int _J \text{ Jac }(s)ds=1\,. $$

Moreover,

$$\begin{aligned}&\lim _{N \rightarrow \infty } \frac{1}{N}\log \text{ Jac }(s) = \lim _{N\rightarrow \infty }\frac{1}{N}\log \Big (\frac{\mathrm{ Vol }(S(\boldsymbol{\sigma }_1,r;\textbf{x}_1,s))}{\mathrm{ Vol }(S(\boldsymbol{\sigma }_1,r))} \Big ) \\&\quad =\frac{1}{2} \log \bigg (\frac{1-\tau (q_1,r,s)}{1-r^2}\bigg ), \end{aligned}$$

uniformly over $s \in J_i$ (since $J_i \not \subset J\setminus J'$ and *k* is large, so $J_i$ is bounded away from $J \setminus J^o$). Consequently,

$$\begin{aligned} \varlimsup _{N\rightarrow \infty }&\sup _{\rho \in J_i} \Big |\, \mathbb {E}\big [\, f_{N,r}(\boldsymbol{\sigma }_1;J_i) \big |\,\mathrm{\textsf {CP}}_\star \,\big ] - \frac{1}{2} \log \Big (\frac{1-\tau (q_1,r,\rho )}{1-r^2}\Big ) \\&- \mathbb {E}\Big [\, \frac{1}{N}\log \Big ( \int _{S(\boldsymbol{\sigma }_1,r;\textbf{x}_1,\rho )}\!\!\!\!\!\!\!\!\!\!\!\!\!\!\!\!\!e^{\beta ' H_N(\boldsymbol{\sigma }')}d\boldsymbol{\sigma }' \Big ) \,\Big |\,\mathrm{\textsf {CP}}_\star \,\Big ] \Big | \le \frac{C}{k} \Big (1+\frac{\beta '}{\sqrt{N}}\mathbb {E}\Big [\,L_N \,\Big |\,\mathrm{\textsf {CP}}_\star \,\Big ]\Big ), \end{aligned}$$

where $L_N$ denotes the Lipschitz constant of $H_N(\boldsymbol{\sigma })$ over the ball of radius $\sqrt{N}$.

Recall that, conditionally on $$\mathrm{\textsf {CP}}_\star $$, we have that $$H_N(\boldsymbol{\sigma }') \!=\! \tilde{H}_N(\boldsymbol{\sigma }') \!+\! \mathbb {E}[H_{N}(\boldsymbol{\sigma }')\,|\,\mathrm{\textsf {CP}}_\star ]$$ for a centered Gaussian process $$\tilde{H}_N(\boldsymbol{\sigma }')$$. Also recall [17, Lemma 6.1] that for large enough $C^{\prime }$, $$\mathbb {E}L_N \le C'\sqrt{N}$$. By Anderson’s inequality, see Remark 2.4, $$\mathbb {E}\tilde{L}_N \le C'\sqrt{N}$$ where $$\tilde{L}_N$$ is the Lipschitz constant of the conditional Hamiltonian $$\tilde{H}_N(\boldsymbol{\sigma }')$$. Moreover, the conditional expectation $$\mathbb {E}[H_{N}(\boldsymbol{\sigma }')\,|\,\mathrm{\textsf {CP}}_\star ]$$ that we computed in (2.13), is also $$C'\sqrt{N}$$-Lipschitz in $$\boldsymbol{\sigma }'$$ (if $C^{\prime }$ is sufficiently large).

Finally, to prove (2.19), it remains to show that

2.22 $$\begin{aligned} \varlimsup _{N\rightarrow \infty } \sup _{\rho \in J_i} \bigg |\,&\mathbb {E}\Big [\, \frac{1}{N}\log \Big ( \int _{S(\boldsymbol{\sigma }_1,r;\textbf{x}_1,\rho )}\!\!\!\!\!\!\!\!\!\!\!\!\!\!\!\!\!e^{\beta ' H_N(\boldsymbol{\sigma }')}d\boldsymbol{\sigma }' \Big ) \,\Big |\,\mathrm{\textsf {CP}}_\star \,\Big ] \nonumber \\&- \Big (\beta ' \langle {\textsf {v}}(q_1,r,\rho ), {\textsf {u}}\rangle + F(\beta ' ; q_1,\tau ,\rho )\Big ) \bigg | =0\, . \end{aligned}$$

Indeed, $\mathbb {E}[ H_N(\boldsymbol{\sigma }') \,\big | \, \mathrm{\textsf {CP}}_\star ]=N \langle {\textsf {v}}(q_1,r,\rho ), {\textsf {u}}\rangle$ for any $\boldsymbol{\sigma }'\in S(\boldsymbol{\sigma }_1,r;\textbf{x}_1,\rho )$ (see (2.13)). Moreover, recall (2.14), that mapping the process $\tilde{H}_N(\boldsymbol{\sigma }')$ from $S(\boldsymbol{\sigma }_1,r;\textbf{x}_1,\rho )$ to the sphere $\mathbb {S}^{N-3}$ while preserving angles, yields a mixed model with mixture $\xi _{q_1,\tau ,\rho }(t)+A$ for some constant A≥0 (see Remark 2.2), up to scaling by a factor of $\sqrt{N/(N-2)}$. As explained after (2.6), the expected free energy for the mixture $\xi _{q_1,\tau ,\rho }(t)+A$ is the same as that for the mixture $\xi _{q_1,\tau ,\rho }(t)$. Hence, (2.22) follows.

## Pure states decomposition

In [46] Talagrand proved an asymptotic pure states decomposition for sequences of random measures on the sphere $\mathbb {S}^{N-1}$ as $N\rightarrow \infty$, assuming they satisfy the Ghirlanda-Guerra identities [28] and that the annealed overlap distribution converges to a law which charges the rightmost point in its support. In particular, his result applies to generic spherical models, if the rightmost point in the support of the Parisi measure is charged. That is, when $\nu _P(q_P)>0$ (which is part of our assumption of finite rsb). Using ultrametricity [35], Jagannath [31] proved that a pure states decomposition exists for generic models also when $\nu _P(q_P)=0$. Below we state these and some additional properties of those decompositions under the strict *k*-rsb condition. To this end, for any m≤k, let

3.1 $$\begin{aligned} \mathbb {A}_{m}:=\Big \{\vec {\textbf{x}}=(\textbf{x}_i)_{i=1}^m\in \mathbb {R}^{N\times m}:\,\frac{1}{N}\langle \textbf{x}_i,\textbf{x}_j\rangle =q_{i\wedge j},\,\forall i,j\le m\Big \} \,. \end{aligned}$$

In particular, if $\vec {\textbf{x}} \in \mathbb {A}_{m}$, then

3.2 $$\begin{aligned} \textbf{x}_m \in \Big \{\textbf{x}_{m-1}+\textbf{y}:\,\textbf{y}\in \sqrt{q_{m}-q_{m-1}} \mathbb {S}^{N-1},\,\langle \textbf{y},\textbf{x}_i\rangle =0,\forall i < m \Big \}\subseteq \sqrt{q_m} \mathbb {S}^{N-1}\,. \end{aligned}$$

Recall that in (1.22), given $\vec {\textbf{x}}=(\textbf{x}_i)_{i=1}^k\in \mathbb {A}_k$ we set, per i≤k, an arbitrary matrix $M_i=M_i(\vec {\textbf{x}})\in \mathbb {R}^{N-i\times N}$ whose rows form an orthonormal basis of $\big (\textrm{sp}\{\textbf{x}_1,\ldots ,\textbf{x}_i\}\big )^{\perp }$ and $\bar{\nabla }H_{N}(\textbf{x}_i):=M_{i}\nabla H_{N}(\textbf{x}_i)$. We likewise define $M_i$ and $\bar{\nabla }H_{N}(\textbf{x}_i)$ for any $1 \le i \le m \le k$ and $\vec {\textbf{x}}\in \mathbb {A}_{m}$. Then, denoting by $\bar{V}(\varepsilon )$ the projection of V(ε) of (1.25) to its last two components, we set

3.3 $$\begin{aligned} \begin{aligned} \mathscr {C}_{m}(\varepsilon ):=\Big \{\vec {\textbf{x}} \in \mathbb {A}_m:\,&\exists (\vec {E}, \vec {R})\in \bar{V}(\varepsilon ) \text{ such } \text{ that } \forall i\le m\\&\Big (\frac{1}{N}H_{N}(\textbf{x}_i),\,\frac{\partial _{\textbf{x}_i-\textbf{x}_{i-1}}H_{N}(\textbf{x}_i)}{\Vert \textbf{x}_i-\textbf{x}_{i-1}\Vert ^2},\,\bar{\nabla }H_{N}(\textbf{x}_i)\Big )=\big (E_i,R_i,0\big ) \Big \}\,. \end{aligned} \end{aligned}$$

From the results of Section 7, the size of $\mathscr {C}_{m}(\varepsilon )$ has finite expectation and is therefore a.s. finite.

For any vertices $\textbf{u}$ and $\textbf{v}$ of a rooted tree we denote by $|\textbf{u}|$ the depth of $\textbf{u}$ and by $\textbf{u}\wedge \textbf{v}$ the least common ancestor, writing $\textbf{u}\le \textbf{v}$ if $\textbf{u}$ is an ancestor of $\textbf{v}$ or $\textbf{u}=\textbf{v}$. Also, if $\mathbb {T}_N$ is a tree with vertex set $\mathbb {V}_N\subset \mathbb {R}^N$ rooted at the origin, for any vertex $\textbf{0}\ne \textbf{v}\in \mathbb {V}_N$ we let $\vec {\textbf{v}} :=(\textbf{v}_1,\ldots ,\textbf{v}_m=\textbf{v})$ record the path from the root to $\textbf{v}$ (excluding the root).

### Proposition 3.1

Consider the spherical mixed *p*-spin model with a generic mixture ξ(t), and $\beta >\beta _{c}$ at which the model is strictly *k*-rsb

for some *k* finite. For any positive $c_N \rightarrow 0$, there exist positive $\delta _N,\varepsilon _{N}\rightarrow 0$ and integer $d_N\rightarrow \infty$, such that the following holds. There exists a full regular tree $\mathbb {T}_N$ of degree $d_N$ and depth *k* with (random) vertex set $\mathbb {V}_N \subset \mathbb {R}^N$ and disjoint (random) sets $B_i\subset \mathbb {S}^{N-1}$ associated with the leaves $\{ \textbf{x}^{\star }_i, i \le d_N^k:= (d_N)^k \}$ of $\mathbb {T}_N$, so with probability tending to 1 as $N \rightarrow \infty$:

1. $$\displaystyle \sum _{i \le d_N^k} G_{N,\beta }\big (B_i\big )>1-\varepsilon _{N}$$   and   $$\displaystyle \min _{i\le d_N^k}G_{N,\beta }(B_i)>c_N$$.
2. $\displaystyle B_i \subset B(\vec {\textbf{x}}^\star _i,\delta _N)$ of (1.24), for all $i \le d_N^k$.
3. $$\displaystyle \sup _{\textbf{u},\textbf{v}\in \mathbb {V}_N} \big |\frac{1}{N} \langle \textbf{u},\textbf{v}\rangle - q_{|\textbf{u}\wedge \textbf{v}|} \big | < \varepsilon _{N}$$.
4. $$\displaystyle \min _{i,j \le d_N^k} G_{N,\beta }^{\otimes 2}\Big ( \big |\frac{1}{N} \langle \boldsymbol{\sigma }_1,\boldsymbol{\sigma }_2\rangle - q_{| \textbf{x}^{\star }_i\wedge \textbf{x}^{\star }_j|} \big |<\varepsilon _{N}\,\Big |\,\boldsymbol{\sigma }_1\in B_i,\,\boldsymbol{\sigma }_2\in B_j \Big )>1-\varepsilon _{N}$$.
5. $$\displaystyle \{ \vec {\textbf{x}}_i^{\star }, i \le d_N^k\} \subset \mathscr {C}_{k}(\varepsilon _N)$$.

In addition, as $N \rightarrow \infty$,

3.4 $$\begin{aligned} \sup _{\textbf{v}\in \mathbb {V}_N}\Big |\frac{1}{N}H_N(\textbf{v})-E_{\star }(q_{|\textbf{v}|})\Big | \longrightarrow 0 \,, \quad \text{ in } \text{ probability. } \end{aligned}$$

Moreover, upon ordering the leaves of ${\mathbb {T}}_N$ so that $i \mapsto G_{N,\beta }(B_i)$ be non-increasing (and padding it with zeros at all $i > d_N^k$), these infinite sequences of weights, converge weakly as $N \rightarrow \infty$ to a Poisson-Dirichlet distribution of parameter $1-\nu _P(q_P)$.

*Proof of Proposition* 3.1, *except Property* (5)

Using the results of [31], even without the full power of (1.21), the pure state decomposition of [42, Corollary 14] has properties (1)–(4) apart from having $\Vert \cdot \Vert ^2/N$ instead of $q_{|\cdot |}$ in both (3) and (4) and the convergence of the weights to a Poisson-Dirichlet distribution.^5^ Going through its construction in the presence of (1.21) allows one to verify, using [42, Point (4’) in p.49], that property (4) must also hold as stated here (for some other non-random $\varepsilon _N \rightarrow 0$). Upon suitably modifying the sequences $\{\varepsilon _N\}$, this implies in turn that (3) must hold as stated, first in case $\textbf{u}=\textbf{v}$, and hence by the triangle inequality, also in its full generality. In Proposition 3.1 we may take $c_N \rightarrow 0$ slowly enough (which in turn implies it with arbitrary $c_N\rightarrow 0$) for [42, Corollary 15] to hold as well. Then, as $\textbf{v}\mapsto H_N(\textbf{v})$ is $O(\sqrt{N})$-Lipschitz whp, combining the statement [42, (1.48)] of [42, Corollary 15] with Property (3) at $\textbf{u}=\textbf{v}$ and the Borell-TIS inequality yields (3.4). □

To summarize, we saw that the points in our embedding of the pure states decomposition, have energy which is (approximately) maximal over the relevant spheres. Property (5) of Proposition 3.1, proved in Section 8, goes further to show that such embedding $\mathbb {V}_N$ exists where also

$$\begin{aligned} \textbf{u}\le \textbf{v}\quad \Longrightarrow \quad \frac{1}{N} \langle \textbf{u},\textbf{v}\rangle = q_{|\textbf{u}|} \end{aligned}$$

(or equivalently, with $\vec {\textbf{v}} \in \mathbb {A}_{|\textbf{v}|}$ for any $\textbf{v}\in \mathbb {V}_N$), and where each $\textbf{x}_m \in \mathbb {V}_N$ of depth *m* is *also a critical point of the energy* on the subset (3.2) of the relevant sphere (as $\bar{\nabla }H_{N}(\textbf{x}_{m})=0$).

## High temperature: proof of Theorem 1.2

First note that since we work with $\beta \le \beta _{c}$, $\mathbb {E}F_{N,\beta }\rightarrow F(\beta )=\frac{1}{2}\beta ^{2}$ and the derivative exists and is equal to $F'(\beta )=\beta$. For $\beta <\beta _{c}$ this follows by definition. For $\beta =\beta _{c}$ it follows since the derivative from the left is $\beta _{c}$, by convexity the derivative of F(β) from the right exists and is at least $\beta _{c}$, and for any β (and in particular on some right neighborhood of $\beta _{c}$) by Jensen’s inequality $\mathbb {E}F_{N,\beta }\le \frac{1}{2}\beta ^{2}$.

The argument from this point up to (4.2) is standard, and is taken almost with no change from [5]. Let ε>0 and define the subsets

$$\begin{aligned} L_{N}^{+}(\varepsilon )&:=\left\{ \boldsymbol{\sigma }\in \Sigma _{N}:\,H_{N}(\boldsymbol{\sigma })/N-F'(\beta )>\varepsilon \right\} ,\\ L_{N}^{-}(\varepsilon )&:=\left\{ \boldsymbol{\sigma }\in \Sigma _{N}:\,H_{N}(\boldsymbol{\sigma })/N-F'(\beta )<-\varepsilon \right\} . \end{aligned}$$

For any λ>0,

$$\begin{aligned} \frac{1}{N}\log \int _{L_{N}^{\pm }(\varepsilon )}e^{\beta H_{N}(\boldsymbol{\sigma })}d\mu _{N}&\le \frac{1}{N}\log \int _{\Sigma _{N}}e^{(\beta \pm \lambda )H_{N}(\boldsymbol{\sigma })\mp \lambda N(F'(\beta )\pm \varepsilon )}d\mu _{N}\\&=F_{N,\beta \pm \lambda }\mp \lambda (F'(\beta )\pm \varepsilon ). \end{aligned}$$

Defining

$$ \delta _{N}^{\pm }(\lambda ):=\frac{F_{N,\beta \pm \lambda }-F_{N,\beta }}{\lambda }\mp F'(\beta ), $$

we have that

$$ F_{N,\beta \pm \lambda }\mp \lambda (F'(\beta )\pm \varepsilon )=F_{N,\beta }-\lambda (\varepsilon -\delta _{N}^{\pm }(\lambda )). $$

Also define

$$ \delta _{\infty }^{\pm }(\lambda ):=\frac{F(\beta \pm \lambda )-F(\beta )}{\lambda }\mp F'(\beta )\overset{\lambda \rightarrow 0}{\longrightarrow }0. $$

Choose some λ>0 sufficiently small so that $a(\varepsilon ,\lambda ):=\varepsilon -\delta _{\infty }^{+}(\lambda )\vee \delta _{\infty }^{-}(\lambda )>0$. Define the event

$$\begin{aligned} \mathcal {E}_{N}(\tau ):= \big \{ |F_{N,\beta \pm \lambda }-F(\beta \pm \lambda )|\vee |F_{N,\beta }-F(\beta )|<\tau \big \}\,. \end{aligned}$$

Since we assume that $\sup _{N,\boldsymbol{\sigma }} \frac{1}{N}\mathbb {E}\big [H_{N}(\boldsymbol{\sigma })^{2}\big ]$ is finite, from the well-known concentration of the free energy [36, Theorem 1.2], for any τ>0,

4.1 $$\begin{aligned} \varlimsup _{N\rightarrow \infty }\frac{1}{N}\log \mathbb {P}(\mathcal {E}_{N}(\tau )^{c})<0 \,. \end{aligned}$$

Taking hereafter $2\tau <\lambda a(\varepsilon ,\lambda )$, on $\mathcal {E}_{N}(\tau )$

$$ \frac{1}{N}\log \int _{L_{N}^{\pm }(\varepsilon )}e^{\beta H_{N}(\boldsymbol{\sigma })}d\mu _{N}\le F_{N,\beta }-\lambda a(\varepsilon ,\lambda )+ 2 \tau $$

and consequently also

4.2 $$\begin{aligned} \varlimsup _{N\rightarrow \infty }\frac{1}{N}\log \mathbb {E}\bigg (1\!\!1_{\mathcal {E}_{N}(\tau )}\cdot \frac{\int _{L_{N}^{+}(\varepsilon )\cup L_{N}^{-}(\varepsilon )}e^{\beta H_{N}(\boldsymbol{\sigma })}d\mu _{N}(\boldsymbol{\sigma })}{\int _{\Sigma _{N}}e^{\beta H_{N}(\boldsymbol{\sigma })}d\mu _{N}(\boldsymbol{\sigma })}\bigg )\le 2\tau -\lambda a(\varepsilon ,\lambda )<0. \end{aligned}$$

In view of (4.1), wlog we can multiply the [0, 1]-valued $G_{N,\beta }\left( f_{N}(\boldsymbol{\sigma })\in A\right)$ in (1.11) by $1\!\!1_{\mathcal {E}_{N}(\tau )}$. Then, by (1.1) and (4.2), we complete the proof upon showing that for $L_{N}(\varepsilon ):=\Sigma _{N}\setminus (L_{N}^{+}(\varepsilon )\cup L_{N}^{-}(\varepsilon ))$,

4.3 $$\begin{aligned} \varlimsup _{N\rightarrow \infty }\frac{1}{N}\log \mathbb {E}\bigg (1\!\!1_{\mathcal {E}_{N}(\tau )}\cdot \frac{\int _{L_{N}(\varepsilon )}e^{\beta H_{N}(\boldsymbol{\sigma })}1\!\!1\{f_{N}(\boldsymbol{\sigma })\in A\}d\mu _{N}(\boldsymbol{\sigma })}{\int _{\Sigma _{N}}e^{\beta H_{N}(\boldsymbol{\sigma })}d\mu _{N}(\boldsymbol{\sigma })}\bigg )<0. \end{aligned}$$

Now, applying (1.7),

$$\begin{aligned} \begin{aligned} \mathbb {E}\int _{L_{N}(\varepsilon )}e^{\beta H_{N}(\boldsymbol{\sigma })}&\cdot 1\!\!1\big \{ f_{N}(\boldsymbol{\sigma })\in A\big \} d\mu _{N}(\boldsymbol{\sigma }) \\&=\int _{\Sigma _{N}}\int _{F'(\beta )-\varepsilon }^{F'(\beta )+\varepsilon }e^{N(\beta E-\frac{1}{2}E^{2})+o(N)}\mathbb {P}\Big (f_{N}(\boldsymbol{\sigma })\in A\,\Big |\,\frac{1}{N}H_{N}(\boldsymbol{\sigma })\\&=E\Big )dE\,d\mu _{N}(\boldsymbol{\sigma }), \end{aligned} \end{aligned}$$

where the *o*(*N*) term is uniform in $\boldsymbol{\sigma }$ and *E*. Since on $\mathcal {E}_{N}(\tau )$ the denominator in (4.3) is at least $e^{(F(\beta ) -\tau )N}$ and

$$ \beta F'(\beta ) - \frac{1}{2} F'(\beta )^2 - F(\beta ) = 0 \,, $$

if we take small enough ε and τ then (4.3) follows from (1.10). □

## Proof sketch for Theorem 1.4 on the low temperature phase

In this section we outline the main steps in the proof of Theorem 1.4.

### Using the pure state decomposition of Proposition 3.1

First, define the measures (which may have mass larger than 1),

5.1 $$\begin{aligned} \bar{\Pi }^\varepsilon _{N,\beta }(\,\cdot \,):= \sum _{\vec {\textbf{x}}\in \mathscr {C}_{k}(\varepsilon )} G_{N,\beta }\big (\,\cdot \,\cap \, B(\vec {\textbf{x}},\varepsilon )\big ) \le \sum _{\vec {\textbf{x}}\in \mathscr {C}_{k}(\varepsilon )} G_{N,\beta }\big (\,\cdot \,\big | \, B(\vec {\textbf{x}},\varepsilon )\big ) =:\Pi ^\varepsilon _{N,\beta }(\,\cdot \,)\,. \end{aligned}$$

Then, denoting by $\mathcal {B}(\mathbb {S}^{N-1})$ the set of Borel measurable subsets of $\mathbb {S}^{N-1}$, it follows from properties (1), (2) and (5) of Proposition 3.1, that for any fixed ε>0,

5.2 $$\begin{aligned} \lim _{N\rightarrow \infty } \mathbb {P}\Big (\forall M\in \mathcal {B}(\mathbb {S}^{N-1}):\, \bar{\Pi }_{N,\beta }^\varepsilon (M)\ge G_{N,\beta }(M)-\varepsilon _{N}\Big )=1. \end{aligned}$$

Hence, to prove (1.27), it suffices to show that

5.3 $$\begin{aligned} \lim _{\varepsilon \rightarrow 0}\varlimsup _{N\rightarrow \infty }\frac{1}{N}\log \mathbb {E}\Big ( \Pi ^{\varepsilon }_{N,\beta }\big (f_{N}(\boldsymbol{\sigma })\in A \big )\Big )<0. \end{aligned}$$

More generally, for (sufficiently regular) $\alpha _k:\mathbb {A}_k\rightarrow [0,1]$ such that the law of $\alpha _k(\vec {\textbf{x}})$ is independent of $\vec {\textbf{x}}$, consider the limit

5.4 $$\begin{aligned} \lim _{\varepsilon \rightarrow 0}\varlimsup _{N\rightarrow \infty }\frac{1}{N}\log \mathbb {E}\Big ( \sum _{\vec {\textbf{x}}\in \mathscr {C}_{k}(\varepsilon )} \alpha _k(\vec {\textbf{x}}) \Big )\,. \end{aligned}$$

Indeed, by (5.1), the expectation of (5.3) is the particular case of

5.5 $$\begin{aligned} \alpha _k(\vec {\textbf{x}}) = G_{N,\beta }\big (\{\boldsymbol{\sigma }:\,f_N(\boldsymbol{\sigma })\in A \}\big | \, B(\vec {\textbf{x}},\varepsilon )\big )\,. \end{aligned}$$

### Applying the Kac-Rice formula

The advantage of dealing with (5.4) rather than (1.27) directly, is that to analyze such sums we may apply the Kac-Rice formula. Indeed, by considering the sequence of gradients

5.6 $$\begin{aligned} \vec {\textbf{x}} \mapsto \big (\bar{\nabla }H_N(\textbf{x}_1),\ldots ,\bar{\nabla }H_N(\textbf{x}_k)\big )\,, \end{aligned}$$

we obtain a mapping from the $D^{\prime }$-dimensional manifold $$\mathbb {A}_k$$ to $$\mathbb {R}^{D'}$$ as required for the application of the Kac-Rice formula, with $$D':=N-1+\cdots +N-k=k(N-\frac{k+1}{2})$$. Further, consider two vector-valued random fields on $$\mathbb {A}_k$$ induced by the real-valued random field $$H_N$$,

5.7 $$\begin{aligned} \vec {\textbf{x}}&\mapsto \big (H_N(\textbf{x}_1),\ldots ,H_N(\textbf{x}_k)\big )\,, \end{aligned}$$

5.8 $$\begin{aligned} \vec {\textbf{x}}&\mapsto \bigg (\frac{\partial _{\textbf{x}_1}H_{N}(\textbf{x}_1)}{\Vert \textbf{x}_1\Vert ^2},\ldots ,\frac{\partial _{\textbf{x}_k-\textbf{x}_{k-1}}H_{N}(\textbf{x}_k)}{\Vert \textbf{x}_k-\textbf{x}_{k-1}\Vert ^2}\bigg )\,. \end{aligned}$$

Then, by definition, $\mathscr {C}_{k}(\varepsilon )$ of (3.3) is the set of points in $\mathbb {A}_k$ such that (5.6) is equal to $0\in \mathbb {R}^{D'}$ and (5.7) normalized by *N* and (5.8) are within distance ε from $(E_{\star }(q_i))_{i\le k}$ and $(R_{\star }(q_i))_{i\le k}$ of (1.17) in sup-norm.

### Warm-up: the 1-RSB case

Before tackling the general case we explain what to do when k=1. Here, we write $\vec {\textbf{x}}=\textbf{x}\in \mathbb {A}_1$. If $\alpha _1(\textbf{x})\equiv 1$, then (5.4) is the ‘usual’ annealed complexity of critical points at radius $q_1$, with energy and radial derivative in a given interval [3, 4, 13]. By (6.4) below, it is equal in this case to

5.9 $$\begin{aligned} \lim _{\varepsilon \rightarrow 0}\varlimsup _{N\rightarrow \infty }\frac{1}{N}\log \Big ( \mathbb {E}\{|\mathscr {C}_{1}(\varepsilon )|\} \Big ) = \Theta _{\xi (q_1\cdot )}(E_{\star }(q_1),q_1R_{\star }(q_1))\,, \end{aligned}$$

for the explicit function $\Theta _\xi (E,R)$ of (6.1).

To obtain an upper bound for (5.4) with general $\alpha _1$ using the Kac-Rice formula, one essentially needs to augment the complexity calculation by an upper bound for the expectation of $\alpha _{1}(\textbf{x})$, under the same conditioning that appears in the Kac-Rice formula.^6^ Precisely, denoting by $\Delta (\alpha _1)$ the limit

5.10 $$\begin{aligned} \lim _{\varepsilon \rightarrow 0}\varlimsup _{N\rightarrow \infty }\frac{1}{N}\log \Big ( \sup _{\begin{array}{c} | E_1-E_{\star }(q_1)|<\varepsilon \\ |R_1-R_{\star }(q_1)|<\varepsilon \end{array}}\mathbb {E}\Big \{ \alpha _1(\textbf{x}) \,\Big |\, \Big (\frac{1}{N}H_{N}(\textbf{x}),\,\frac{\partial _{\textbf{x}}H_{N}(\textbf{x})}{\Vert \textbf{x}\Vert ^2},\,\bar{\nabla }H_{N}(\textbf{x})\Big )=\big (E_1,R_1,0\big ) \Big \}\Big ) \,, \end{aligned}$$

one can show that

5.11 $$\begin{aligned} (5.4)\le \Theta _{\xi (q_1\cdot )}(E_{\star }(q_1),q_1R_{\star }(q_1))+\Delta (\alpha _1)\,. \end{aligned}$$

For the case of interest to us (5.5), we shall see that under the conditioning as in (5.10), the Gibbs measure $G_{N,\beta }(\,\cdot \,\big | \, B(\textbf{x},\varepsilon ))$ on $B(\textbf{x},\varepsilon )$ is effectively in the high-temperature (rs) phase. Thus, by a similar argument to that used for Theorem 1.2 (which needs to be slightly strengthened to account for supremum), for (5.5) we show in Section 9 that if (1.26) holds then $\Delta (\alpha _1)<0$.

### The case of general k≥1

To bound (5.4) one may in principle apply the Kac-Rice formula directly on the space $\mathbb {A}_k$, but it is more natural to instead use an inductive argument on *k*. Specifically, for any $\vec {\textbf{y}}=(\textbf{y}_1,\ldots ,\textbf{y}_m)\in \mathbb {A}_m$ and $m\le n\le k$, we define, similarly to (3.1) and (3.3),

5.12 $$\begin{aligned} \begin{aligned}&\mathbb {A}_{m,n}(\vec {\textbf{y}}):=\Big \{\vec {\textbf{x}}=(\textbf{x}_i)_{i=1}^{n}\in \mathbb {A}_{n}:\,(\textbf{x}_1,\ldots ,\textbf{x}_m)=(\textbf{y}_1,\ldots ,\textbf{y}_m)\Big \}\\&\mathscr {C}_{m,n}(\varepsilon ,\vec {\textbf{y}}):=\Big \{\vec {\textbf{x}} \in \mathbb {A}_{m,n}(\vec {\textbf{y}}):\,\exists (\vec {E}, \vec {R})\in \bar{V}(\varepsilon ) \text{ such } \text{ that } \forall m+1\le i\le n,\\&\quad \Big (\frac{1}{N}H_{N}(\textbf{x}_i),\,\frac{\partial _{\textbf{x}_i-\textbf{x}_{i-1}}H_{N}(\textbf{x}_i)}{\Vert \textbf{x}_i-\textbf{x}_{i-1}\Vert ^2},\,\bar{\nabla }H_{N}(\textbf{x}_i)\Big )=\big (E_i,R_i,0\big ) \Big \}\,. \end{aligned} \end{aligned}$$

We may then write

5.13 $$\begin{aligned} \begin{aligned} \sum _{\vec {\textbf{x}}\in \mathscr {C}_{k}(\varepsilon )} \alpha _k(\vec {\textbf{x}}) = \sum _{\textbf{y}\in \mathscr {C}_{1}(\varepsilon )} \alpha _{1,k}^{\varepsilon }(\textbf{y}) = \sum _{\textbf{y}\in \mathscr {C}_{1}(\infty )} 1\!\!1\{\textbf{y}\in \mathscr {C}_{1}(\varepsilon )\} \cdot \alpha _{1,k}^{\varepsilon }(\textbf{y})\,, \end{aligned} \end{aligned}$$

where $\mathscr {C}_{1}(\infty )$ is the set of critical points of $H_N$ over $\mathbb {A}_1=\sqrt{q_1}\mathbb {S}^{N-1}$ and

5.14 $$\begin{aligned} \alpha _{1,k}^{\varepsilon }(\textbf{y}) := \sum _{\vec {\textbf{x}}\in \mathscr {C}_{1,k}(\varepsilon ,\textbf{y})} \alpha _k(\vec {\textbf{x}})\,. \end{aligned}$$

While (5.13) holds with $\alpha _{1,k}^{\varepsilon }(\textbf{y})$ defined by summing over $\{\vec {\textbf{x}}\in \mathscr {C}_{k}(\varepsilon ):\,\textbf{x}_1=\textbf{y}\}$ in (5.14), such $\alpha _{1,k}^{\varepsilon }$ being zero outside $\mathscr {C}_{1}(\infty )$, hence lacks the continuity over $\mathbb {A}_1$ needed for applying the Kac-Rice formula.

Fix $$\textbf{y}\in \sqrt{q_1}\mathbb {S}^{N-1}$$. Define

$$ \varphi (\textbf{z}) = \textbf{y}+\sqrt{\frac{N(1-q_1)}{N-1}}\theta (\textbf{z}) $$

for an arbitrary linear isometry $\theta :\mathbb {R}^{N-1}\rightarrow \textbf{y}^{\perp }=\{\textbf{x}:\textbf{x}\perp \textbf{y}\}$, and note that for this choice of the scale factor $\varphi (\mathbb {S}^{N-2})=(\textbf{y}+\textbf{y}^{\perp })\cap \mathbb {S}^{N-1}$. By abuse of notation, for vectors $\vec {\textbf{z}} = (\textbf{z}_1,\ldots ,\textbf{z}_{k-1})$ define $\varphi (\vec {\textbf{z}})=(\varphi (\textbf{z}_i))$ by element-wise operation. To make the dependence in $H_N$ and $q=(q_i)_{i=1}^k$ explicit, we use the notation $\mathscr {C}_{k}(\infty ;H_N,q)$ and $\mathscr {C}_{1,k}(\infty ,\textbf{y};H_N,q)$, where here taking $\varepsilon =\infty$ the values of $\vec {E}$ and $\vec {R}$ are irrelevant. Define $\hat{q}=(\hat{q}_i)_{i=1}^{k-1}$, where $\hat{q}_i= \frac{q_{i}-q_1}{1-q_1}$. One can verify that $\vec {\textbf{x}}\in \mathscr {C}_{1,k}(\infty ,\textbf{y};H_N,q)$ with

$$ \Big (\frac{1}{N}H_N(\textbf{y}),\,\frac{1}{N}H_{N}(\textbf{x}_i),\,\frac{\partial _{\textbf{x}_i-\textbf{x}_{i-1}}H_{N}(\textbf{x}_i)}{\Vert \textbf{x}_i-\textbf{x}_{i-1}\Vert ^2}\Big ) = (\bar{E},\,\bar{E}_i,\, \bar{R}_i) $$

if and only if $\vec {\textbf{x}}=\varphi (\vec {\textbf{z}})$ for $\vec {\textbf{z}}\in \mathscr {C}_{k-1}(\infty ;H_N^\textbf{y},\hat{q})$ such that

$$ \Big (\frac{1}{N-1}H^{\textbf{y}}_{N}(\textbf{z}_i),\,\frac{\partial _{\textbf{z}_i-\textbf{z}_{i-1}}H^{\textbf{y}}_{N}(\textbf{z}_i)}{\Vert \textbf{z}_i-\textbf{z}_{i-1}\Vert ^2}\Big ) = \sqrt{\frac{N}{N-1}}\big (\bar{E}_{i+1}-\bar{E},\, (1-q_1)\bar{R}_{i+1}\big )\,. $$

Hence, setting $\hat{E}=(E_{i+1}-E_1)_{i=1}^{k-1}$ and $\hat{R}=((1-q_1)R_{i+1})_{i=1}^{k-1}$, we have that

$$ \alpha _{1,k}^{\varepsilon }(\textbf{y}) = \sum _{\vec {\textbf{x}}\in \mathscr {C}_{1,k}(\varepsilon ,\textbf{y};H_N,q,\vec {E},\vec {R})} \alpha _{k}(\vec {\textbf{x}}) \le \sum _{\vec {\textbf{z}}\in \mathscr {C}_{k-1}(4\varepsilon ,\textbf{y};H^\textbf{y}_N,\hat{q},\hat{E},\hat{R})} \hat{\alpha }_{k-1}(\vec {\textbf{z}})\,, $$

where $\hat{\alpha }_{k-1}(\vec {\textbf{z}}):=\alpha _{k}(\varphi (\vec {\textbf{z}}))$ and where we included $\vec {E}$ and $\vec {R}$ in the notation to make the dependence on them explicit. The sum on rhs is of the same form as in (5.4), with *k* reduced by 1 and modified Hamiltonian and parameters.

Proposition 7.1 states an upper bound on (5.4) which is proved by an inductive argument using the above idea. Specifically, it yields a generalization of (5.11) for k≥1:

5.15 $$\begin{aligned} (5.4)\le \sum _{i=0}^{k-1} \Theta _{\xi _{(i)}}\Big (E_{\star }(q_{i+1})-E_{\star }(q_{i}),(q_{i+1}-q_i)R_{\star }(q_{i+1})\Big ) +\Delta (\alpha _k)\,, \end{aligned}$$

where $\xi _{(i)}$ are explicit mixtures defined by (6.5) and (6.6) and, generalizing (5.10), $\Delta (\alpha _k)$ is defined as the limit

$$\begin{aligned} \lim _{\varepsilon \rightarrow 0}\varlimsup _{N\rightarrow \infty }\frac{1}{N}\log \bigg ( \sup _{\bar{V}(\varepsilon )}\mathbb {E}\Big \{ \alpha _k(\vec {\textbf{x}}) \,\Big |\, \mathrm{\textsf {CP}}(\vec {\textbf{x}},\vec {E},\vec {R}) \Big \}\bigg )\,, \end{aligned}$$

for the event

$$\begin{aligned} \mathrm{\textsf {CP}}(\vec {\textbf{x}},\vec {E},\vec {R}) := \bigcap _{1\le i \le k} \Big \{ \Big (\frac{1}{N}H_{N}(\textbf{x}_i),\,\frac{\partial _{\textbf{x}_i-\textbf{x}_{i-1}}H_{N}(\textbf{x}_i)}{\Vert \textbf{x}_i-\textbf{x}_{i-1}\Vert ^2},\,\bar{\nabla }H_{N}(\textbf{x}_i)\Big )=\big (E_i,R_i,0\big ) \Big \}\,. \end{aligned}$$

By the same argument mentioned above for the case k=1, also for k≥1 we show in Section 9 that if (1.26) holds then $\Delta (\alpha _k)<0$.

### Vanishing of the annealed complexities

Recall the zero-temperature Parisi formula (1.16). Analogously to the set $\mathcal {S}_{P}=\mathcal {S}_{P,\xi ,\beta }$ of (1.20) which was defined for finite $\beta >\infty$, a zero-temperature analogue $\mathcal {S}_{P,\xi ,\infty }\subset [0,1]$ can be defined from the optimality condition for the Parisi measure, see (6.10).

Using the characterization (6.10) of the Parisi measure, it was proved in [42] that the mixtures $\xi _{(m)}$ as in (5.15) are strictly 1-rsb in the sense that $\mathcal {S}_{P,\xi _{(m)},\infty }=\{0,1\}$, see Corollary 6.2 and (6.13).

Making the dependence on the mixture explicit by writing $E_{\star }(q)=E_{\star }(\xi ,q)$ and $R_{\star }(q)=R_{\star }(\xi ,q)$, in Lemma 6.8 we show that

$$\begin{aligned} E_{\star }(\xi _{(m)},1)&=E_{\star }(\xi ,q_{m+1})-E_{\star }(\xi ,q_{m}),\\ R_{\star }(\xi _{(m)},1)&=(q_{m+1}-q_{m})R_{\star }(\xi ,q_{m+1}). \end{aligned}$$

Finally, by a result of Huang and Sellke [30], which we restate in Lemma 6.3, if $\mathcal {S}_{P,\xi _{(m)},\infty }=\{0,1\}$, then $\Theta _{\xi }(E_{\star }(1),R_{\star }(1))=0$.

By combining these facts, we conclude in Corollary 6.9 that each of the complexities in the sum of (5.15) is equal to zero. Since, as stated above, if (1.26) holds then $\Delta (\alpha _k)<0$, in Section 9 we conclude from this that (5.4) is strictly negative in the setting of Theorem 1.4, thereby completing its proof.

## Complexity of critical points and related results

In this section we collect several results from [13, 30, 42] about the complexity of critical points and prove some consequences of theirs that will be used later. For any mixture ξ(t) and intervals $$I,I'\subset \mathbb {R}$$ denote the number of critical points with normalized energy in *I* and normal derivative in $I^{\prime }$ by

$$\begin{aligned} \textrm{Crt}_{N,\xi }(I,I')&:=\#\Big \{\boldsymbol{\sigma }\in \mathbb {S}^{N-1}:\,\forall \textbf{u}\perp \boldsymbol{\sigma },\,\langle \nabla H_N(\boldsymbol{\sigma }),\textbf{u}\rangle =0, \\&\frac{1}{N} H_N(\boldsymbol{\sigma })\in I, \frac{1}{N}\langle \nabla H_N(\boldsymbol{\sigma }),\boldsymbol{\sigma }\rangle \in I' \Big \}. \end{aligned}$$

For a mixture ξ(t) which is not pure, define the function

6.1 $$\begin{aligned} \Theta _\xi (E,R)=\frac{1}{2}+\frac{1}{2}\log \Big (\frac{\xi ''(1)}{\xi '(1)}\Big )-\frac{1}{2}(E,R)\Sigma _{\xi }^{-1}(E,R)^{\top }+\Omega (R/\sqrt{\xi ''(1)}), \end{aligned}$$

where (see, e.g., [26, Prop. II.1.2])

6.2 $$\begin{aligned} \begin{aligned} \Omega (t)&=\frac{1}{2\pi } \int _{-2}^{2} \log |\lambda -t| \sqrt{4-\lambda ^2}d\lambda \\&= {\left\{ \begin{array}{ll} \frac{t^2}{4}-\frac{1}{2} & |t|\le 2\\ \frac{t^2}{4}-\frac{1}{2} - \Big [\frac{|t|}{4}\sqrt{t^2-4}-\log \Big (\sqrt{\frac{t^2}{4}-1}+\frac{|t|}{2}\Big )\Big ] & |t|>2, \end{array}\right. } \end{aligned} \end{aligned}$$

and

6.3 $$\begin{aligned} \Sigma _{\xi }=\left( \begin{matrix} \xi (1) &  \xi '(1) \\ \xi '(1) &  \xi ''(1) +\xi '(1) \end{matrix} \right) . \end{aligned}$$

In the pure case $\xi (t)=t^p$, define

$$\begin{aligned} \Theta _\xi (E,R)={\left\{ \begin{array}{ll} \frac{1}{2}+\frac{1}{2}\log \Big (\frac{\xi ''(1)}{\xi '(1)}\Big )-\frac{1}{2} E^2/\xi (1)+\Omega (R/\sqrt{\xi ''(1)}) & R=pE\\ -\infty & \text{ otherwise. } \end{array}\right. } \end{aligned}$$

Theorem 2.8 of [4]^7^ and Theorem 3.1 of [13] state that for any intervals $I,I'\subset \mathbb {R}$,

6.4 $$\begin{aligned} \lim _{N\rightarrow \infty }\frac{1}{N}\log \mathbb {E}\textrm{Crt}_{N,\xi }(I,I') = \sup _{(E,R) \in I\times I'}\Theta _\xi (E,R). \end{aligned}$$

For any q∈[0,1), define the mixtures

6.5 $$\begin{aligned} \xi _q(t)=\xi (t+q)-\xi (q)-\xi '(q)t, \qquad \bar{\xi }_q(t) :=\xi _q((1-q)t) \,. \end{aligned}$$

Assuming the strict *k*-rsb condition (1.21) and setting $q_{k+1}=1$, we define for any $0\le m\le k$ the mixtures

6.6 $$\begin{aligned} \begin{aligned} \xi _{(m)}(t)&:=\xi _{q_m}((q_{m+1}-q_m)t),\\ \bar{\xi }_{(m)}(t)&:=\bar{\xi }_{q_m}(t). \end{aligned} \end{aligned}$$

We recall the definitions (3.1) and (3.3) of

6.7 $$\begin{aligned} \mathbb {A}_{m}:=\Big \{\vec {\textbf{x}}=(\textbf{x}_i)_{i=1}^m\in \mathbb {R}^{N\times m}:\,\frac{1}{N}\langle \textbf{x}_i,\textbf{x}_j\rangle =q_{i\wedge j},\,\forall i,j\le m\Big \} \, \end{aligned}$$

and

6.8 $$\begin{aligned} \begin{aligned} \mathscr {C}_{m}(\varepsilon ):=\Big \{\vec {\textbf{x}} \in \mathbb {A}_m:\,&\exists (\vec {E}, \vec {R})\in \bar{V}(\varepsilon ) \text{ such } \text{ that } \forall i\le m\\&\Big (\frac{1}{N}H_{N}(\textbf{x}_i),\,\frac{\partial _{\textbf{x}_i-\textbf{x}_{i-1}}H_{N}(\textbf{x}_i)}{\Vert \textbf{x}_i-\textbf{x}_{i-1}\Vert ^2},\,\bar{\nabla }H_{N}(\textbf{x}_i)\Big )=\big (E_i,R_i,0\big ) \Big \}\,. \end{aligned} \end{aligned}$$

They involve discrete paths $\vec {\textbf{x}}=(\textbf{x}_1,\ldots ,\textbf{x}_m)$ such that each $\textbf{x}_{n+1}$ is a critical point of $H_N(\textbf{x})$ on the (N-n-1)-dimensional sphere

$$\begin{aligned} \Big \{ \textbf{x}_{n}+\textbf{y}: \Vert \textbf{y}\Vert =\sqrt{N (q_{n+1}-q_{n})}, \,\langle \textbf{y},\textbf{x}_i\rangle =0,\,\forall i\le n \Big \} \,. \end{aligned}$$

In the sequel we show that under an appropriate conditioning, the expected number of critical points on the latter sphere is expressed by the formula (6.4) for mixture $$\xi _{(n)}$$ with *I* and $I^{\prime }$ appropriately scaled (see (7.19)), hence by summing over the different levels, one obtains an asymptotic formula for $$\mathbb {E}|\mathscr {C}_{m}(\varepsilon )|$$ (see Remark 7.2).

Mixtures as in (6.6) were central to [42], where certain free energy functionals on spherical bands were studied and a generalized tap approach was established (also see [16, 17] for the Ising case). For example, the free energy mixture corresponding to $\bar{\xi }_{(m)}$ was used to define in [42] a tap correction that generalizes the classical Onsager correction term. These mixtures were also used before in the 1-rsb case [13, 40] to study the free energy of spherical bands around critical points.

Most relevant to our current discussion are certain relations between the Parisi measure of $\xi _{(m)}$ at zero-temperature and that of ξ at inverse-temperature β, which were established in [42]. Recall the set of overlaps $\mathcal {S}_{P}=\mathcal {S}_{P,\xi ,\beta }$ defined by the optimality condition of Talagrand in (1.20), where we now add ξ and β to the notation, to make the distinction from its zero-temperature analogue and between the mixtures ξ and $\bar{\xi }_{(m)}$.

### Corollary 6.1

([42]) If $x_\beta$ is the Parisi distribution of ξ at β and $q\in \mathcal {S}_{P,\xi ,\beta }$, then

$$ \bar{x}_\beta ^q(t) = x_\beta (q+(1-q)t) $$

is the Parisi distribution of $\bar{\xi }_q$ of (6.5) (at β), and

6.9 $$\begin{aligned} \forall t\in [0,1]:\quad t\in \mathcal {S}_{P,\bar{\xi }_q,\beta }\iff q+(1-q)t\in \mathcal {S}_{P,\xi ,\beta }. \end{aligned}$$

### Proof

The first claim, about the relation between the Parisi measures, is part of [42, Proposition 11]. Fixing ξ and β, let $\phi :=\phi _{\nu _{\beta ,P}}$ denote the function defined by (1.19) for the Parisi measure $\nu _{\beta ,P}$ and define $\phi _q(s):=\phi (q+(1-q)s)-\phi (q)$. Recall that $\mathcal {S}_{P,\xi ,\beta }=\{s\in [0,1]:\phi (s)=\sup _{t\in [0,1]}\phi (t)\}$. In the proof of [42, Proposition 11] it was shown that $\mathcal {S}_{P,\bar{\xi }_q,\beta }=\{s\in [0,1]:\phi _q(s)=\sup _{t\in [0,1]}\phi _q(t)\}$ from which (6.9) follows. □

An analogue of the optimality condition [45, Proposition 2.1] was also proved for the zero-temperature Parisi formula (1.16). For any non-decreasing, right continuous and integrable $\alpha :[0,1)\rightarrow [0,\infty )$ and c>0 define

$$\begin{aligned} \Psi _{\alpha ,c}(t)&=\xi '(t)-\int _0^t\frac{ds}{(\int _s^1\alpha (r)dr+c)^2},\\ \psi _{\alpha ,c}(s)&=\int _s^1\Psi _{\alpha ,c}(t)dt, \end{aligned}$$

and let $\eta _{\alpha }$ be the measure on [0, 1) defined by $\eta _{\alpha }([0,s])=\alpha (s)$. It was proved in [18] that the minimizer in the Parisi formula (1.16) is the unique pair (α,c) such that $\Psi _{\alpha ,c}(1)=\min _{s\in [0,1]}\psi _{\alpha ,c}(s)=0$ and

6.10 $$\begin{aligned} \text{ supp }(\eta _{\alpha })\subset \mathcal {S}^{\infty }_{\alpha ,c}:=\{s\in [0,1]:\,\psi _{\alpha ,c}(s)=0\}. \end{aligned}$$

Denote by $\mathcal {S}_{P,\xi ,\infty }$ the set $\mathcal {S}^{\infty }_{\alpha ,c}$ that corresponds to the minimizer (α,c).

### Corollary 6.2

([42]) If $x_\beta$ is the Parisi distribution of ξ at β and $0<q\in \mathcal {S}_{P,\xi ,\beta }$, then the minimizer of the Parisi formula (1.16) for $\hat{\xi }_q(t)= \xi (qt)$ is

$$ (\alpha _q(t),c_q)=\Big ( \beta x_\beta (qt),\frac{\beta }{q}\int _q^1 x_\beta (s)ds \Big ) \,. $$

Further,

6.11 $$\begin{aligned} \forall t\in [0,1]:\quad t\in \mathcal {S}_{P,\hat{\xi }_q,\infty }\iff qt\in \mathcal {S}_{P,\xi ,\beta }. \end{aligned}$$

### Proof

The first claim, about the relation between the Parisi measures, is part of [42, Proposition 11]. Fixing ξ and β, recall that with $\phi =\phi _{\nu _{\beta ,P}}$ as above, $\mathcal {S}_{P,\xi ,\beta }=\{s\in [0,1]:\phi (s)=\sup _{t\in [0,1]}\phi (t)\}$. In the proof of [42, Proposition 11], it was shown that $\mathcal {S}_{P,\hat{\xi }_q,\infty }=\{s\in [0,1]:\psi _q(s)=\min _{t\in [0,1]}\psi _q(t)\}$ for $\psi _q(s):=-\phi (qs)/\beta ^2$ (using ϕ(q)=0), from which (6.11) follows. □

Assuming (1.21), Corollary 6.1 implies that for $1 \le m\le k$, the Parisi distribution of $\bar{\xi }_{(m)}$ at β is

6.12 $$\begin{aligned} \begin{aligned} x_\beta ^m(t)&= x_\beta (q_m+(1-q_m)t), \qquad with \\ \mathcal {S}_{P,\bar{\xi }_{(m)},\beta }&= \bigg \{\frac{q_i-q_m}{1-q_m}:\,i=m,\ldots ,k \bigg \}. \end{aligned} \end{aligned}$$

Corollary 6.2 with $\xi =\bar{\xi }_{(m)}$ and $q=(q_{m+1}-q_{m})/(1-q_{m})$ then implies that the minimizer of the zero-temperature Parisi formula for $\xi _{(m)}$ is

6.13 $$\begin{aligned} (\alpha ^m_q(t),c^m_q)&=\Big ( \beta x_\beta (q_m + (q_{m+1}-q_m) t ),\frac{\beta }{q_{m+1}-q_m}\int _{q_{m+1}}^{1} x_\beta (s)ds \Big ),\quad with \nonumber \\ \mathcal {S}_{P,\xi _{(m)},\infty }&= \{0,1\}. \end{aligned}$$

Models such as (6.13), where $\mathcal {S}_{P,\xi ,\infty }=\{0,1\}$ are called in [30] strictly 1-rsb. The next two results of Huang and Sellke [30] about the complexity of critical points of such models will be important to us.

### Lemma 6.3

([30]) If ξ is a mixture such that $\mathcal {S}_{P,\xi ,\infty }=\{0,1\}$ and $E_{\star }(1)$ and $R_{\star }(1)$ are defined by (1.17), then

$$\begin{aligned} \Theta _{\xi }(E_{\star }(1),R_{\star }(1))=0. \end{aligned}$$

### Proof

Assuming $\mathcal {S}_{P,\xi ,\infty }=\{0,1\}$, it is shown in [30, Lemma 3.18] that $\Theta _{\xi }(E_0,R_0)=0$ for $(E_0,R_0)$ of [30, (3.6)]. Thus, it suffices to verify that

6.14 $$\begin{aligned} (E_0,R_0)=(E_{\star }(1),R_{\star }(1)). \end{aligned}$$

Indeed, by [30, Lemma 3.5], $E_0=E_{\star }(1)$ and L,u>0 of that lemma are such that the minimizer of the zero-temperature Parisi formula (1.16) for ξ is $\alpha (t)\equiv u$ and c=L-u. In the proof of [30, Lemma 3.5] it is further shown that $\xi '(1)=(L(L-u))^{-1}$. We thus get from [30, (3.6)] that $R_0=(c+u)\xi '(1)+c\xi ''(1)$. In comparison, for $\alpha (t)\equiv u$ we get from (1.18) (at q=1), that also $R_{\star }(1)=(c+u)\xi '(1)+c\xi ''(1)$. □

Relying on (6.14), we next re-state [30, Proposition 3.14] in our notation, while writing $\textrm{Crt}_{N,\xi }(E,R)$ for $\textrm{Crt}_{N,\xi }(\{E\},\{R\})$ with singletons.

### Proposition 6.4

([30]) Assume that $\mathcal {S}_{P,\xi ,\infty }=\{0,1\}$. For any sufficiently small η>0 there exist some $a(\eta ),b(\eta )>0$ such that for any $a\le a(\eta )$, $b\le b(\eta )$ and $\boldsymbol{\sigma }\in \mathbb {S}^{N-1}$,

$$ \lim _{N\rightarrow \infty }\sup _{\begin{array}{c} |E-E_{\star }(1)|\le a\\ |R-R_{\star }(1)|\le a \end{array}}\frac{1}{N}\log \bigg ( \mathbb {P}\bigg [ \frac{1}{N}\sup _{\begin{array}{c} \textbf{x}\in \mathbb {S}^{N-1}:\\ \langle \textbf{x},\boldsymbol{\sigma }\rangle =(1-\eta ) N \end{array}} \{ H_N(\textbf{x}) \} \ge E_{\star }(1)-b\,\bigg |\,\boldsymbol{\sigma }\in \textrm{Crt}_{N,\xi }(E,R) \bigg ] \bigg )<0. $$

We observe that the gradient, and thus the radial derivative, are roughly determined at approximate ground states in the following sense.

### Lemma 6.5

Let q∈(0,1) and define

$$ L_{N}(q,\varepsilon ):=\left\{ \textbf{x}\in \sqrt{q}\mathbb {S}^{N-1}:\,\Big |\frac{1}{N}H_{N}(\textbf{x})-E_{\star }(q)\Big |<\varepsilon \right\} . $$

Then, for any fixed $\varepsilon ',\tau >0$, for sufficiently small ε>0,

6.15 $$\begin{aligned} \varlimsup _{N\rightarrow \infty }\frac{1}{N}\log \bigg (\mathbb {P}\bigg [\sup _{\textbf{x}\in L_{N}(q,\varepsilon )}\Big |\frac{1}{\Vert \textbf{x}\Vert ^2}\partial _{\textbf{x}}H_{N}(\textbf{x})-R_{\star }(q)\Big |>\varepsilon '\bigg ]\bigg )&<0, \end{aligned}$$

6.16 $$\begin{aligned} \varlimsup _{N\rightarrow \infty }\frac{1}{N}\log \bigg (\mathbb {P}\bigg [\sup _{\textbf{x}\in L_{N}(q,\varepsilon )} \sup _{\textbf{y}:\,\textbf{y}\perp \textbf{x}}\Big |\frac{1}{\sqrt{N}\Vert \textbf{y}\Vert }\partial _{\textbf{y}}H_{N}(\textbf{x})\Big |>\tau \bigg ]\bigg )&<0. \end{aligned}$$

### Remark 6.6

We follow the usual convention that the supremum over an empty set is -∞ (but this does not really matter, since by the Borell-TIS inequality, $\mathbb {P}(L_{N}(q,\varepsilon )=\varnothing ) \le e^{-c N}$ for some c=c(ε)>0).

### Proof

Fix $\varepsilon^{\prime }>0$ and assume towards contradiction that for any ε>0,

6.17 $$\begin{aligned} \varlimsup _{N\rightarrow \infty }\frac{1}{N}\log \bigg (\mathbb {P}\bigg [\sup _{\textbf{x}\in L_{N}(q,\varepsilon )} \frac{1}{\Vert \textbf{x}\Vert ^2}\partial _{\textbf{x}}H_{N}(\textbf{x})-R_{\star }(q) >\varepsilon '\bigg ]\bigg )=0. \end{aligned}$$

By [13, Corollary C.2], for some constants C=C(ξ) and c=c(ξ), denoting $\mathbb {B}^{N}:=\{\textbf{x}:\,\Vert \textbf{x}\Vert \le \sqrt{N}\}$,

6.18 $$\begin{aligned} \mathbb {P}\Big (\forall \textbf{x},\textbf{y}\in \mathbb {B}^{N}:\,\Vert \nabla H_{N}(\textbf{x})-\nabla H_{N}(\textbf{y})\Vert _{\textrm{op}}<C\Vert \textbf{x}-\textbf{y}\Vert \Big )>1-e^{-cN}. \end{aligned}$$

Let t>0 be a small number to be determined below. Define $q_{t}:=(\sqrt{q}+t)^2$. Recall the definition (1.17) of $E_{\star }(q)$. By the Borell-TIS inequality, for any δ>0, for some $c'=c'(\xi ,\delta )>0$,

6.19 $$\begin{aligned} \mathbb {P}\left( \max _{\textbf{x}\in \sqrt{q}\mathbb {S}^{N-1}}\frac{1}{N}H_{N}\Big (\textbf{x}\!+\!t\sqrt{N}\frac{\textbf{x}}{\Vert \textbf{x}\Vert }\Big )\!=\!\max _{\textbf{x}\in \sqrt{q_{t}}\mathbb {S}^{N-1}}\frac{1}{N}H_{N} (\textbf{x})\!<\!E_{\star }(q_{t})\!+\!\delta \right) \!\ge \! 1\!-\!e^{-c'N}. \end{aligned}$$

On some subsequence of $N\rightarrow \infty$, with positive probability the three events in (6.17)-(6.19) occur simultaneously. From (6.18) we have that whp for all $\textbf{x}\in \mathbb {B}^{N}$,

6.20 $$\begin{aligned} \begin{aligned} H_{N}\Big (\textbf{x}&+t\sqrt{N}\frac{\textbf{x}}{\Vert \textbf{x}\Vert }\Big ) = H_{N}(\textbf{x})+\int _0^{t\sqrt{N}} \partial _{\textbf{x}/\Vert \textbf{x}\Vert } H_{N}\Big (\textbf{x}+s\frac{\textbf{x}}{\Vert \textbf{x}\Vert }\Big ) ds \\&=H_{N}(\textbf{x})+t\sqrt{N} \partial _{\textbf{x}/\Vert \textbf{x}\Vert } H_{N}(\textbf{x}) + \int _0^{t\sqrt{N}} \bigg [\int _0^{s} \partial _{\textbf{x}/\Vert \textbf{x}\Vert }^2 H_{N}\Big (\textbf{x}+w\frac{\textbf{x}}{\Vert \textbf{x}\Vert }\Big ) dw\bigg ]ds \\&\ge H_{N}(\textbf{x})+ t\sqrt{q}N\frac{1}{\Vert \textbf{x}\Vert ^2}\partial _{\textbf{x}}H_{N}(\textbf{x})- \frac{N}{2} C t^2 \end{aligned} \end{aligned}$$

where we used that $t\sqrt{N}\partial _{\textbf{x}/\Vert \textbf{x}\Vert }H_{N}(\textbf{x})=t\sqrt{q}N\frac{1}{\Vert \textbf{x}\Vert ^2}\partial _{\textbf{x}}H_{N}(\textbf{x})$. Thus, when the events in (6.17)-(6.18) occur there exists a point $\textbf{x}\in L_{N}(q,\varepsilon )$ such that

6.21 $$\begin{aligned} \frac{1}{N}H_{N}\Big (\textbf{x}+t\sqrt{N}\frac{\textbf{x}}{\Vert \textbf{x}\Vert }\Big )\ge E_{\star }(q)-\varepsilon +t\sqrt{q}\big (R_{\star }(q)+\varepsilon '\big )-\frac{1}{2}Ct^{2}\,. \end{aligned}$$

Since $\frac{d}{dt}q_{t}|_{t=0}=2\sqrt{q}$ and $R_{\star }(q):=2\frac{d}{dq}E_{\star }(q)$, for all small enough t>0,

$$ E_{\star }(q_{t})\le E_{\star }(q)+t\sqrt{q}\big (R_{\star }(q)+\varepsilon '/2\big ). $$

Combining the last two inequalities and canceling like terms, the point as in (6.21) satisfies

$$\begin{aligned} \frac{1}{N}H_{N}\Big (\textbf{x}+t\sqrt{N}\frac{\textbf{x}}{\Vert \textbf{x}\Vert }\Big )&\ge E_{\star }(q_t)+t\sqrt{q}\varepsilon '/2-\varepsilon -\frac{1}{2}Ct^{2} \ge E_{\star }(q_t)+\delta , \end{aligned}$$

for $\varepsilon^{\prime }$ fixed and $$\varepsilon ,\delta ,t$$ appropriately chosen, (first taking *t* sufficiently small so that $$t\sqrt{q}\varepsilon '/2 -\frac{1}{2}Ct^{2}>0$$ and then $$\varepsilon ,\delta $$ small enough) in contradiction to the event in (6.19). It follows that, for some ε>0, (6.17) does not hold. A similar argument proves the same bound for the opposite inequality (with $-\varepsilon^{\prime }$) and (6.15) follows.

The bound of (6.16) can be proved by a similar idea. Fix τ>0 and assume towards contradiction that for any ε>0,

6.22 $$\begin{aligned} \varlimsup _{N\rightarrow \infty }\frac{1}{N}\log \bigg (\mathbb {P}\bigg [\sup _{\textbf{x}\in L_{N}(q,\varepsilon )} \sup _{\textbf{y}:\,\textbf{y}\perp \textbf{x}}\Big |\frac{1}{\sqrt{N}\Vert \textbf{y}\Vert }\partial _{\textbf{y}}H_{N}(\textbf{x})\Big |>\tau \bigg ]\bigg )=0. \end{aligned}$$

Similarly to (6.20), from (6.18) we have that whp for all $\textbf{x}\in \mathbb {B}^{N}$ and $\textbf{y}\perp \textbf{x}$,

$$\begin{aligned} H_{N}\Big (\textbf{x}+t\sqrt{N}\frac{\textbf{y}}{\Vert \textbf{y}\Vert }\Big ) \ge H_{N}(\textbf{x})+ t\sqrt{N} \partial _{\textbf{y}/\Vert \textbf{y}\Vert } H_{N}(\textbf{x})- \frac{N}{2} C t^2\,. \end{aligned}$$

From (6.22), whp for some $\textbf{x}\in L_{N}(q,\varepsilon )$ and $\textbf{y}\perp \textbf{x}$,

$$\begin{aligned} \frac{1}{N}H_{N}\Big (\textbf{x}\!+\!t\sqrt{N}\frac{\textbf{y}}{\Vert \textbf{y}\Vert }\Big ) \!\ge \! E_{\star }(q)\!-\!\varepsilon \!+\! t\tau \!-\! \frac{1}{2} C t^2\!\ge \! E_{\star }(q\!+\!t^2)\!+\!2\delta \!-\!\varepsilon \!\ge \! E_{\star }(q\!+\!t^2)\!+\!\delta \,, \end{aligned}$$

where the second inequality follows for sufficiently small *t* and δ and the third for small ε. Since $\textbf{x}+t\sqrt{N}\textbf{y}/\Vert \textbf{y}\Vert \in \sqrt{q+t^2}\mathbb {S}^{N-1}$, we arrive at a contradiction and (6.16) follows. □

Similarly to $\mathscr {C}_{m}(\varepsilon )$ of (6.8), let

6.23 $$\begin{aligned} \begin{aligned} \hat{\mathscr {C}}_{m}(\varepsilon ) :=\Big \{\vec {\textbf{x}} \in \mathbb {A}_m:\,&\exists (\vec {E}, \vec {R})\in \bar{V}(\varepsilon ) \text{ such } \text{ that } \forall i\le m \\&\Big (\frac{1}{N}H_{N}(\textbf{x}_i),\,\frac{\partial _{\textbf{x}_i}H_{N}(\textbf{x}_i)}{\Vert \textbf{x}_i\Vert ^2},\,\bar{\nabla }H_{N}(\textbf{x}_i)\Big )=\big (E_i,R_i,0\big ) \Big \} \end{aligned} \end{aligned}$$

and

6.24 $$\begin{aligned} \begin{aligned} \mathscr {C}^0_{m}(\varepsilon ) :=\Big \{\vec {\textbf{x}} \in \mathbb {A}_m:\,&\exists E_i\in [E_{\star }(q_i)-\varepsilon , E_{\star }(q_i)+\varepsilon ] \text{ such } \text{ that } \forall i\le m \\&\Big (\frac{1}{N}H_{N}(\textbf{x}_i),\,\bar{\nabla }H_{N}(\textbf{x}_i)\Big )=\big (E_i,0\big ) \Big \}\,. \end{aligned} \end{aligned}$$

### Corollary 6.7

For any $$1 \le m\le k$$ and fixed $\varepsilon^{\prime }>0$, for sufficiently small ε>0,

$$ \varlimsup _{N\rightarrow \infty } \frac{1}{N}\log \bigg (1-\mathbb {P}\Big [ \mathscr {C}_{m}(\varepsilon )\cup \hat{\mathscr {C}}_{m}(\varepsilon )\subset \mathscr {C}^0_{m}(\varepsilon ) \subset \mathscr {C}_{m}(\varepsilon ')\cap \hat{\mathscr {C}}_{m}(\varepsilon ') \Big ]\bigg )<0. $$

### Proof

The first containment follows deterministically by definition of the various sets. For $\mathscr {C}^0_{m}(\varepsilon ) \subset \hat{\mathscr {C}}_{m}(\varepsilon ')$, since $(\vec {E}, \vec {R})\in \bar{V}(\varepsilon )$ the bound on the probability follows directly from (6.15) (see (1.25)).

To prove that $\mathscr {C}^0_{m}(\varepsilon ) \subset \mathscr {C}_{m}(\varepsilon ')$ with overwhelming probability, take some small τ and assume that ε is sufficiently small so that (6.16) holds. Let $\vec {\textbf{x}}=(\textbf{x}_1,\ldots ,\textbf{x}_m)\in \mathscr {C}^0_{m}(\varepsilon )$, so that in particular $\textbf{x}_i\in L_N(q_i,\varepsilon )$. Define the projection $\textbf{z}_i=\langle \textbf{x}_i-\textbf{x}_{i-1},\textbf{x}_i\rangle \textbf{x}_i/\Vert \textbf{x}_i\Vert ^2$ and $\textbf{y}_i=\textbf{x}_i-\textbf{x}_{i-1}-\textbf{z}_i\perp \textbf{x}_i$. Since $\vec {\textbf{x}}\in \mathbb {A}_m$, $\Vert \textbf{x}_i-\textbf{x}_{i-1}\Vert ^2=\langle \textbf{z}_i,\textbf{x}_i\rangle =N(q_i-q_{i-1})$, $\Vert \textbf{x}_i\Vert ^2=Nq_i$ and $\Vert \textbf{y}_i\Vert ^2=N(q_i-q_{i-1})q_{i-1}/q_i$. Thus,

$$\begin{aligned} \frac{\partial _{\textbf{x}_i-\textbf{x}_{i-1}}H_{N}(\textbf{x}_i)}{\Vert \textbf{x}_i-\textbf{x}_{i-1}\Vert ^2}&=\frac{1}{N(q_i-q_{i-1})}\Big (\langle \textbf{z}_i,\nabla H_N(\textbf{x}_i) \rangle +\langle \textbf{y}_i,\nabla H_N(\textbf{x}_i) \rangle \Big )\\&=\frac{1}{N(q_i-q_{i-1})}\Big (\langle \textbf{z}_i,\textbf{x}_i \rangle \frac{\partial _{\textbf{x}_i}H_{N}(\textbf{x}_i)}{\Vert \textbf{x}_i\Vert ^2}+\partial _{\textbf{y}_i} H_N(\textbf{x}_i) \Big )\\&=\frac{\partial _{\textbf{x}_i}H_{N}(\textbf{x}_i)}{\Vert \textbf{x}_i\Vert ^2}+\sqrt{\frac{q_{i-1}}{q_i(q_i-q_{i-1})}}\frac{1}{\sqrt{N}\Vert \textbf{y}_i\Vert }\partial _{\textbf{y}_i}H_{N}(\textbf{x}_i). \end{aligned}$$

Recall that $\big |\frac{\partial _{\textbf{x}_i}H_{N}(\textbf{x}_i)}{\Vert \textbf{x}_i\Vert ^2}-R_{\star }(q_i)\big |<\varepsilon$. Hence, if ε and τ are sufficiently small, on the complement of the event in (6.16), $\vec {\textbf{x}}\in \mathscr {C}_{m}(\varepsilon ')$. □

For later use we also relate the ground state energy and its derivative for the mixtures $\xi _{(m)}$ (see (6.6)) to those of the mixture ξ and to make explicit the dependence on ξ, below we use the notations $E_{\star }(\xi ,q)$ and $R_{\star }(\xi ,q)$.

### Lemma 6.8

Assuming (1.21), we have that

6.25 $$\begin{aligned} E_{\star }(\xi _{(m)},1)&=E_{\star }(\xi ,q_{m+1})-E_{\star }(\xi ,q_{m}), \end{aligned}$$

6.26 $$\begin{aligned} R_{\star }(\xi _{(m)},1)&=(q_{m+1}-q_{m})R_{\star }(\xi ,q_{m+1}). \end{aligned}$$

### Proof

While the lemma can be proved using the Parisi formula, we instead use the tap representation of [42, Theorem 5] (which by [42, Theorem 10] applies to overlaps in $\mathcal {S}_{P,\xi ,\beta }$). Specifically, denote by F(ξ) the limiting free energy of the spherical model with mixture ξ at inverse-temperature β and, recalling the definition (6.5) of $\bar{\xi }_q(t)$, define the operators

$$\begin{aligned} \mathcal {A}_q&:\xi (t)\mapsto \bar{\xi }_q(t), \\ \mathcal {K}_q&:\xi (t)\mapsto \xi (qt). \end{aligned}$$

The tap representation of [42, Theorems 5 and 10] state that for any q∈[0,1),

6.27 $$\begin{aligned} F(\xi )\ge \beta E_{\star }(\xi ,q)+\frac{1}{2}\log (1-q)+F(\mathcal {A}_q(\xi )), \end{aligned}$$

and for any $q\in \mathcal {S}_{P,\xi ,\beta }$

6.28 $$\begin{aligned} F(\xi )= \beta E_{\star }(\xi ,q)+\frac{1}{2}\log (1-q)+F(\mathcal {A}_q(\xi )). \end{aligned}$$

By applying the tap representation again to the free energy on the rhs (namely, to the mixture $\mathcal {A}_q(\xi )$), we obtain that for any $\bar{q}\in [0,1)$,

6.29 $$\begin{aligned}&F(\xi )\ge \beta E_{\star }(\xi ,q)+\frac{1}{2}\log (1-q)+\beta E_{\star }(\mathcal {A}_q(\xi ),\bar{q})+\frac{1}{2}\log (1-\bar{q})\nonumber \\&\quad +F(\mathcal {A}_{\bar{q}}\circ \mathcal {A}_q(\xi )), \end{aligned}$$

with equality if both $q\in \mathcal {S}_{P,\xi ,\beta }$ and $\bar{q}\in \mathcal {S}_{P,\mathcal {A}_q(\xi ),\beta }$.

By assumption, $q_m,q_{m+1}\in \mathcal {S}_{P,\xi ,\beta }$ and by (6.12) also $\bar{q}:= \frac{q_{m+1}-q_m}{1-q_m}\in \mathcal {S}_{P,\bar{\xi }_{(m)},\beta }$. One can verify by a direct calculation that $\mathcal {A}_{\bar{q}}\circ \mathcal {A}_{q_m}(\xi )= \mathcal {A}_{q_{m+1}}(\xi )$, $\mathcal {K}_{\bar{q}}\mathcal {A}_{q_m}(\xi )=\xi _{(m)}$ and

6.30 $$\begin{aligned} \begin{aligned} E_{\star }(\mathcal {A}_{q_m}(\xi ),\bar{q})&= E_{\star }(\mathcal {K}_{\bar{q}}\mathcal {A}_{q_m}(\xi ),1)=E_{\star }(\xi _{(m)},1),\\ \log (1-\bar{q})&=\log (1-q_{m+1})-\log (1-q_m). \end{aligned} \end{aligned}$$

Hence, by using the first representation (6.28) for F(ξ) with $q=q_{m+1}$ and the second representation (6.29) with $q=q_m$ and $\bar{q}$ as above, we arrive at (6.25) upon canceling the like-terms.

Now, for $q>q_m$, consider the second representation (6.29) with *q* replaced by $q_m$ and $\bar{q}:= \frac{q-q_{m}}{1-q_m}$. Namely,

6.31 $$\begin{aligned}&F(\xi )\ge \beta E_{\star }(\xi ,q_m)+\frac{1}{2}\log (1-q_m) +\beta E_{\star }(\mathcal {A}_{q_m}(\xi ),\bar{q})+\frac{1}{2}\log (1-\bar{q})\nonumber \\&\quad +F(\mathcal {A}_{\bar{q}}\circ \mathcal {A}_{q_m}(\xi ))\nonumber \\&=\beta E_{\star }(\xi ,q_m)+\beta E_{\star }\Big (\xi _{(m)},\frac{q-q_m}{q_{m+1}-q_m}\Big )+\frac{1}{2}\log (1-q)+F(\mathcal {A}_{q}(\xi )) \end{aligned}$$

where we used $\mathcal {A}_{\bar{q}}\circ \mathcal {A}_{q_m}(\xi )= \mathcal {A}_{q}(\xi )$ and similarly to (6.30),

$$\begin{aligned} \begin{aligned} E_{\star }(\mathcal {A}_{q_m}(\xi ),\bar{q})&= E_{\star }(\mathcal {K}_{\bar{q}}\mathcal {A}_{q_m}(\xi ),1)=E_{\star }\Big (\xi _{(m)},\frac{q-q_m}{q_{m+1}-q_m}\Big ),\\ \log (1-\bar{q})&=\log (1-q)-\log (1-q_m). \end{aligned} \end{aligned}$$

Both representations (6.28) and (6.31) hold with equality for $q=q_{m+1}$. Since the lhs does not depend on *q*, the derivative in *q* of the rhs of each of them is equal to zero. In particular, by comparing those two derivatives and canceling like-terms we have

$$ \frac{d}{dq}\Big |_{q=q_{m+1}}E_{\star }(\xi ,q)= \frac{d}{dq}\Big |_{q=q_m}E_{\star }\Big (\xi _{(m)},\frac{q-q_m}{q_{m+1}-q_m}\Big ), $$

from which (6.26) follows by the definition of $R_{\star }(q):=2\frac{d}{dq}E_{\star }(q)$. □

For later use, we record the following immediate corollary of Lemmas 6.8 and 6.3 and (6.13).

### Corollary 6.9

Assuming (1.21), for $E_{\star }(q_m)$ and $R_{\star }(q_m)$ as in (1.17),

$$ \Theta _{\xi _{(m)}}\Big (E_{\star }(q_{m+1})-E_{\star }(q_{m}),(q_{m+1}-q_m)R_{\star }(q_{m+1})\Big )=0. $$

## Kac-Rice formulas

The results of this section do not assume condition (1.21). Thus, while we fix throughout some $0=q_0< q_1<\cdots<q_k<1$, these $\{q_i\}$ do not have to be related to the Parisi measure. We recall that $\bar{\nabla }H_{N}(\textbf{x}_i):=M_{i}\nabla H_{N}(\textbf{x}_i)$ for $M_i=M_i(\vec {\textbf{x}})\in \mathbb {R}^{(N-i)\times N}$ an arbitrary matrix whose rows form an orthonormal basis of $\big (\textrm{sp}\{\textbf{x}_1,\ldots ,\textbf{x}_i\}\big )^{\perp }$ and by an abuse of notation, for m≤k and $\vec {\textbf{x}}\in \mathbb {A}_m$ of (6.7), define the event

7.1 $$\begin{aligned} \mathrm{\textsf {CP}}(\vec {\textbf{x}},\vec {E},\vec {R}) := \bigcap _{1\le i \le m} \Big \{ \Big (\frac{1}{N}H_{N}(\textbf{x}_i),\,\frac{\partial _{\textbf{x}_i-\textbf{x}_{i-1}}H_{N}(\textbf{x}_i)}{\Vert \textbf{x}_i-\textbf{x}_{i-1}\Vert ^2},\,\bar{\nabla }H_{N}(\textbf{x}_i)\Big )=\big (E_i,R_i,0\big ) \Big \}\,. \end{aligned}$$

While the set of indices *i* in (7.1) is determined by the size of the vector $\vec {\textbf{x}}$, the vectors $\vec {E}$ and $\vec {R}$ are allowed to be longer (and $\mathscr {C}_m(\varepsilon )$ of (6.8) is merely the subset of $\mathbb {A}_m$ for which $\cup _{\bar{V}(\varepsilon )} \mathrm{\textsf {CP}}(\vec {\textbf{x}},\vec {E},\vec {R})$ occurs). Fixing n≤k we consider a non-random $\bar{\alpha }_{N,n}(\vec {\textbf{x}},\varphi ): \mathbb {A}_n\times C^\infty (\mathbb {B}^{N}) \mapsto \mathbb {R}_+$ which as in Theorem 1.4 is rotationally invariant in the sense that $\bar{\alpha }_{N,n}(\vec {\textbf{x}},\varphi )=\bar{\alpha }_{N,n}(O\vec {\textbf{x}},\varphi (O^T\cdot ))$ for any orthogonal *O* (where $O\vec {\textbf{x}}:=(O\textbf{x}_1,\ldots ,O\textbf{x}_n)$), and such that for any $\varphi ,\psi _{i} \in C^\infty (\mathbb {B}^{N})$ and d≥1,

7.2 $$\begin{aligned} \lim _{t_{i}\rightarrow 0}\bar{\alpha }_{N,n}\Big (\vec {\textbf{x}},\varphi +\sum _{i\le d}t_{i}\psi _{i}\Big )=\bar{\alpha }_{N,n}(\vec {\textbf{x}},\varphi ) \,. \end{aligned}$$

Our goal in this section is to prove the following multi-level Kac-Rice formula. To lighten the notation we often omit *N* from various functions, such as $\alpha _{n}(\vec {\textbf{x}}):=\bar{\alpha }_{N,n}(\vec {\textbf{x}},H_{N}(\cdot ))$.

### Proposition 7.1

Suppose that $\alpha _{n}(\vec {\textbf{x}})$ as above, is a.s. continuous in the topology induced on $\mathbb {A}_n$ from $\mathbb {R}^{N\times n}$. For ε>0 and 0≤m<n, set

7.3 $$\begin{aligned} I_m(\varepsilon )&=[E_{\star }(q_{m+1})-E_{\star }(q_m)-2\varepsilon ,E_{\star }(q_{m+1})-E_{\star }(q_m)+2\varepsilon ], \end{aligned}$$

7.4 $$\begin{aligned} I'_m(\varepsilon )&=(q_{m+1}-q_m)[R_{\star }(q_{m+1})-\varepsilon ,R_{\star }(q_{m+1})+\varepsilon ]. \end{aligned}$$

Then, for any finite *M*,

7.5 $$\begin{aligned}&\varlimsup _{N\rightarrow \infty }\frac{1}{N}\log \mathbb {E}\Big [ \big (\sum _{\vec {\textbf{x}}\in \mathscr {C}_{n}(\varepsilon )} \alpha _n(\vec {\textbf{x}}) \big ) \wedge e^{MN} \Big ] \nonumber \\&\le \sum _{m=0}^{n-1}\sup _{(E,R) \in I_m(\varepsilon )\times I'_m(\varepsilon )}\Theta _{\xi _{(m)}}(E,R) \nonumber \\&\quad + \varlimsup _{N\rightarrow \infty }\frac{1}{N}\log \Big (\sup _{\bar{V}(\varepsilon )}\mathbb {E}\Big [ \alpha _n(\vec {\textbf{x}}_{n}^{\textbf{e}}) \wedge e^{MN} \,\Big |\,\mathrm{\textsf {CP}}(\vec {\textbf{x}}_{n}^\textbf{e},\vec {E},\vec {R}) \Big ]\Big )\,. \end{aligned}$$

### Remark 7.2

In particular, taking $\bar{\alpha }_{N,n}(\cdot )\equiv 1$ we arrive at

$$ \varlimsup _{N\rightarrow \infty }\frac{1}{N}\log \mathbb {E}\Big [ |\mathscr {C}_{n}(\varepsilon )| \wedge e^{MN}\Big ] \le \sum _{m=0}^{n-1} \sup _{(E,R) \in I_m(\varepsilon )\times I'_m(\varepsilon )}\Theta _{\xi _{(m)}}(E,R) \,. $$

For $q_i$ the atoms of the Parisi measure as in (1.21), typically $\mathscr {C}_{n}(\varepsilon )\ne \varnothing$ (for it contains the paths of the tree of pure states, as in Proposition 3.1), but as $\varepsilon \rightarrow 0$, the preceding bound on its exponential in *N* growth rate, goes to zero.

In Section 7.3 we prove Proposition 7.1 by induction on *m*, where in each step we apply the one-level Kac-Rice formulas of Section 7.2. Towards this, we first provide in Section 7.1 explicit representations of $H_N(\cdot )$ and its derivatives, on the relevant sub-spaces ($S_0(\vec {\textbf{x}}_m^\textbf{e})$ below), conditional on $\mathrm{\textsf {CP}}(\vec {\textbf{x}}_m^\textbf{e},\vec {E},\vec {R})$.

### Conditional laws

We start with the conditional law of $H_N(\cdot )$ given $\mathrm{\textsf {CP}}(\vec {\textbf{x}},\vec {E},\vec {R})$, for $\vec {\textbf{x}} \in \mathbb {A}_m$, on certain (N-m)-dimensional linear sub-spaces.

#### Lemma 7.3

Let m≤k and $\vec {\textbf{x}}=(\textbf{x}_1,\ldots ,\textbf{x}_m)\in \mathbb {A}_m$. For any

7.6 $$\begin{aligned} \textbf{y}_1,\textbf{y}_2\in S_0(\vec {\textbf{x}}):=\Big \{\textbf{y}\in \mathbb {R}^N:\,\forall i\le m,\, \frac{1}{N}\langle \textbf{y}, \textbf{x}_i \rangle =q_i\Big \}, \end{aligned}$$

(such that $\xi (\Vert \textbf{y}_i\Vert ^2/N)<\infty$) we have that

7.7 $$\begin{aligned} \mathbb {E}\Big [ H_N(\textbf{y}_1)\,\Big |\, \mathrm{\textsf {CP}}(\vec {\textbf{x}},\vec {E},\vec {R})\Big ]=N E_m \end{aligned}$$

and

7.8 $$\begin{aligned}&\mathbb {E}\Big [(H_N(\textbf{y}_1)-N E_m)(H_N(\textbf{y}_2)-N E_m)\,\Big |\, \mathrm{\textsf {CP}}(\vec {\textbf{x}},\vec {E},\vec {R})\Big ]\nonumber \\&= N\Big (\xi (\langle \textbf{y}_1,\textbf{y}_2\rangle /N)-\xi (q_m)-\xi '(q_m)\langle \textbf{y}_1-\textbf{x}_m,\textbf{y}_2-\textbf{x}_m\rangle /N \Big )\nonumber \\&=N\xi _{q_m}(\langle \textbf{y}_1,\textbf{y}_2\rangle /N-q_m), \end{aligned}$$

for the mixture $\xi _q$ of (6.5).

#### Proof

We implicitly assume throughout that $\xi (\Vert \textbf{y}_i\Vert ^2/N)<\infty$. Consider first the case m=1 and set $[\textbf{y},\textbf{x}]:=\langle \textbf{y},\textbf{x}\rangle /N$. We then have that for any unit vectors $\textbf{u}\perp \textbf{x}_1$, $\textbf{u}_1,\textbf{u}_2$ and $\textbf{y}_1\in S_0(\textbf{x}_1)$,

7.9 $$\begin{aligned}&\mathbb {E}\big [(H_N(\textbf{y}_1)-H_N(\textbf{x}_1))H_N(\textbf{x}_1)\big ]=N(\xi ( [\textbf{y}_1,\textbf{x}_1])-\xi ([ \textbf{x}_1,\textbf{x}_1]))=0, \end{aligned}$$

7.10 $$\begin{aligned}&\mathbb {E}\big [H_N(\textbf{x}_1) \partial _{\textbf{u}_1}H_N(\textbf{x})|_{\textbf{x}=\textbf{x}_1}\big ]=N\partial _{\textbf{u}_1}\xi ([\textbf{x}_1,\textbf{x}])|_{\textbf{x}=\textbf{x}_1}=\xi '(q_1)\langle \textbf{x}_1,\textbf{u}_1\rangle , \end{aligned}$$

7.11 $$\begin{aligned}&\mathbb {E}\big [(H_N(\textbf{y}_1)\!-\!H_N(\textbf{x}_1))\partial _{\textbf{u}}H_N(\textbf{x})|_{\textbf{x}\!=\!\textbf{x}_1}\big ]\!=\!\xi '(q_1)\langle \textbf{y}_1,\textbf{u}\rangle \!-\! \xi '(q_1) \langle \textbf{x}_1,\textbf{u}\rangle \!=\! \xi '(q_1)\langle \textbf{y}_1,\textbf{u}\rangle , \end{aligned}$$

7.12 $$\begin{aligned}&\mathbb {E}\big [(H_N(\textbf{y}_1)-H_N(\textbf{x}_1))\partial _{\textbf{x}_1}H_N(\textbf{x})|_{\textbf{x}=\textbf{x}_1}\big ]=\xi '(q_1)\langle \textbf{y}_1,\textbf{x}_1\rangle - \xi '(q_1) \langle \textbf{x}_1,\textbf{x}_1 \rangle =0, \end{aligned}$$

7.13 $$\begin{aligned}&\quad \mathbb {E}\big [\partial _{\textbf{u}_1}H_N(\textbf{x})|_{\textbf{x}=\textbf{x}_1}\partial _{\textbf{u}_2}H_N(\textbf{y})|_{\textbf{y}=\textbf{x}_1}\big ]=N\partial _{\textbf{u}_1}\partial _{\textbf{u}_2}\xi ([\textbf{x},\textbf{y}])|_{\textbf{x}=\textbf{x}_1,\textbf{y}=\textbf{x}_1} \nonumber \\&=\xi '(q_1)\langle \textbf{u}_1,\textbf{u}_2\rangle +\frac{1}{N}\xi ''(q_1)\langle \textbf{x}_1,\textbf{u}_1\rangle \langle \textbf{x}_1,\textbf{u}_2\rangle , \end{aligned}$$

where $\partial _{\textbf{v}}f(\textbf{x})=\langle \nabla f(\textbf{x}),\textbf{v}\rangle$ and on the rhs we apply $\partial _{\textbf{u}_1}$ wrt the argument $\textbf{x}$ and $\partial _{\textbf{u}_2}$ wrt $\textbf{y}$.

Hence, $H_N(\textbf{y}_1)-H_N(\textbf{x}_1)$ and $\bar{\nabla }H_{N}(\textbf{x}_1)$ are independent of $H_{N}(\textbf{x}_1)$ and $\partial _{\textbf{x}_1} H_{N}(\textbf{x}_1)$. The conditional mean of $H_N(\textbf{y}_1)-H_N(\textbf{x}_1)$ given $\mathrm{\textsf {CP}}(\vec {\textbf{x}},\vec {E},\vec {R})$ is therefore equal to its conditional mean given that $\bar{\nabla }H_{N}(\textbf{x}_1)=0$. That is, it is equal to 0 and (7.7) follows. Moreover, the conditional covariance of $H_N(\textbf{y}_1)-H_N(\textbf{x}_1)$ and $H_N(\textbf{y}_2)-H_N(\textbf{x}_1)$ given $\mathrm{\textsf {CP}}(\vec {\textbf{x}},\vec {E},\vec {R})$ is equal to the conditional covariance only given that $\bar{\nabla }H_{N}(\textbf{x}_1)=0$. From the well-known formulas for the conditional law of jointly Gaussian variables,

$$\begin{aligned}&\mathbb {E}\big [(H_N(\textbf{y}_1)-H_N(\textbf{x}_1))(H_N(\textbf{y}_2)-H_N(\textbf{x}_1)) \,|\,\bar{\nabla }H_{N}(\textbf{x}_1)=0 \big ]\\&=\mathbb {E}\big [(H_N(\textbf{y}_1)-H_N(\textbf{x}_1))(H_N(\textbf{y}_2)-H_N(\textbf{x}_1))\big ] \\&- \mathbb {E}\big [(H_N(\textbf{y}_1)-H_N(\textbf{x}_1))\bar{\nabla }H_{N}(\textbf{x}_1)^{\top }\big ] \big (\mathbb {E}\big [\bar{\nabla }H_{N}(\textbf{x}_1)\bar{\nabla }H_{N}(\textbf{x}_1)^{\top }\big ]\big )^{-1}\mathbb {E}\big [(H_N(\textbf{y}_2)\\&-H_N(\textbf{x}_1))\bar{\nabla }H_{N}(\textbf{x}_1)\big ]\,. \end{aligned}$$

Note that the first expectation on the right-side is $N \xi ([\textbf{y}_1,\textbf{y}_2])-N \xi (q_1)$. Further, the entries of $\mathbb {E}\big [\bar{\nabla }H_{N}(\textbf{x}_1)\bar{\nabla }H_{N}(\textbf{x}_1)^{\top }\big ]$ correspond to (7.13) for an orthonormal basis $\{\textbf{u}_j\}$ of $\textbf{x}_1^{\perp }$, so this matrix is merely $\xi '(q_1)\textbf{I}$. Likewise, by (7.11),

$$ \mathbb {E}\big [(H_N(\textbf{y}_i)-H_N(\textbf{x}_1))\bar{\nabla }H_{N}(\textbf{x}_1)^{\top }\big ] = \xi '(q_1) (\langle \textbf{y}_i,\textbf{u}_1\rangle ,\ldots , \langle \textbf{y}_i,\textbf{u}_{N-1}\rangle ) \,, $$

and we arrive at (7.8) upon utilizing the obvious identity $\langle \textbf{y}_1-\textbf{x}_1,\textbf{y}_2-\textbf{x}_1\rangle = \sum _{j} \langle \textbf{y}_1,\textbf{u}_j\rangle \langle \textbf{y}_2,\textbf{u}_j\rangle$.

We proceed by induction on *m*, setting $\vec {\textbf{x}}_m=(\textbf{x}_1,\ldots ,\textbf{x}_m)\in \mathbb {A}_m$, $\vec {\textbf{x}}_{m+1}=(\textbf{x}_1,\ldots ,\textbf{x}_{m+1}) \in \mathbb {A}_{m+1}$ and assuming that the lemma holds for *m*. Defining $\hat{H}_N(\textbf{y})=H_N(\textbf{y}+\textbf{x}_m)-H_N(\textbf{x}_m)$, we have by our induction hypothesis that conditional on $\mathrm{\textsf {CP}}(\vec {\textbf{x}}_{m},\vec {E},\vec {R})$, on $S_{\perp }(\vec {\textbf{x}}_m):=\{\textbf{y}:\,\textbf{y}\perp \textbf{x}_i,\forall i\le m\}$, the process $\hat{H}_N(\textbf{y})$ has the same law as the mixed model with the mixture $\xi _{q_m}$ of (6.5). Note that the conditional law given $\mathrm{\textsf {CP}}(\vec {\textbf{x}}_{m+1},\vec {E},\vec {R})$ can be obtained by first conditioning on $\mathrm{\textsf {CP}}(\vec {\textbf{x}}_{m},\vec {E},\vec {R})$ and then further conditioning on

$$\begin{aligned} \Big (\frac{1}{N}\hat{H}_{N}(\hat{\textbf{x}}_{m+1}),\,\frac{\partial _{\hat{\textbf{x}}_{m+1}}\hat{H}_{N}(\hat{\textbf{x}}_{m+1})}{\Vert \hat{\textbf{x}}_{m+1}\Vert ^2},\,\bar{\nabla }\hat{H}_N(\hat{\textbf{x}}_{m+1}) \Big )=\big (\hat{E}_{m+1}, R_{m+1},0\big ), \end{aligned}$$

where $\hat{\textbf{x}}_{m+1}:=\textbf{x}_{m+1}-\textbf{x}_{m}$, $\hat{E}_{m+1}:= E_{m+1}-E_m$ and $\bar{\nabla }\hat{H}_N(\textbf{y}):= M_{m+1}(\vec {\textbf{x}}_{m+1})\nabla \hat{H}_N(\textbf{y})$ (with $M_{m+1}$ as in (7.1)). Hence, by the lemma at m=1, we have that for any $\textbf{y}_1,\textbf{y}_2\in S_{\perp }(\vec {\textbf{x}}_m)$ such that $[\textbf{y}_i,\hat{\textbf{x}}_{m+1}]=q_{m+1}-q_m$,

$$\begin{aligned} \mathbb {E}\Big [ \hat{H}_N(\textbf{y}_1)\,\Big |\, \mathrm{\textsf {CP}}(\vec {\textbf{x}}_{m+1},\vec {E},\vec {R})\Big ]=N \hat{E}_{m+1} \end{aligned}$$

and, denoting $$\hat{q}:=q_{m+1}-q_m$$,

7.14 $$\begin{aligned} \begin{aligned}&\mathbb {E}\Big [(\hat{H}_N(\textbf{y}_1)-N \hat{E}_{m+1})(\hat{H}_N(\textbf{y}_2)-N \hat{E}_{m+1})\,\Big |\, \mathrm{\textsf {CP}}(\vec {\textbf{x}}_{m+1},\vec {E},\vec {R})\Big ] \\&=N\Big ( \xi _{q_{m}}([ \textbf{y}_1,\textbf{y}_2])-\xi _{q_{m}}(\hat{q})-\xi _{q_{m}}'(\hat{q}) [ \textbf{y}_1-\hat{\textbf{x}}_{m+1},\textbf{y}_2-\hat{\textbf{x}}_{m+1}] \Big ). \end{aligned} \end{aligned}$$

Now, note that $\check{\textbf{y}}_i:= \textbf{y}_i+\textbf{x}_m\in S_0(\vec {\textbf{x}}_{m+1})$, $[\textbf{y}_1,\textbf{y}_2]=[ \check{\textbf{y}}_1,\check{\textbf{y}}_2]-q_m$ and

$$\begin{aligned} {[} \textbf{y}_1-\hat{\textbf{x}}_{m+1},\textbf{y}_2-\hat{\textbf{x}}_{m+1}] = [ \check{\textbf{y}}_1,\check{\textbf{y}}_2]-q_{m+1}. \end{aligned}$$

By substituting the above and using (6.5), we obtain that the rhs of (7.14) is equal to

$$ N\Big (\xi ([ \check{\textbf{y}}_1,\check{\textbf{y}}_2])-\xi (q_{m+1})-\xi '(q_{m+1})([ \check{\textbf{y}}_1,\check{\textbf{y}}_2]-q_{m+1})\Big )=N\xi _{q_{m+1}}([ \check{\textbf{y}}_1,\check{\textbf{y}}_2]-q_{m+1}). $$

This establishes the lemma for m+1 and concludes the proof. □

Recall that $\vec {\textbf{x}}_m^\textbf{e}=(\textbf{x}_1^\textbf{e},\ldots , \textbf{x}_m^\textbf{e})\in \mathbb {A}_m$ for $\textbf{x}^{\textbf{e}}_i:= \sum _{j=1}^i \sqrt{N(q_j-q_{j-1})} \textbf{e}_j$. We next re-write the representation of Lemma 7.3 in terms of the linear mapping $T^{(m)}:\mathbb {R}^{N-m} \rightarrow S_0(\vec {\textbf{x}}_m^{\textbf{e}})$, given by

810

$$\begin{aligned} T^{(m)}(\textbf{z})&= T^{(m)}((z_1,\ldots ,z_{N-m})) = \textbf{x}_{m}^{\textbf{e}}+\sqrt{{\textstyle \frac{N}{N-m}} (q_{m+1}-q_m)}(0,\ldots ,0,z_1,\ldots ,z_{N-m})\,, \end{aligned}$$

and note in passing that $T^{(m)}$ maps $\mathbb {S}^{N-m-1}=\{\textbf{x}\in \mathbb {R}^{N-m}: \Vert \textbf{x}\Vert ^2=N-m\}$ to $S(\vec {\textbf{x}}_{m}^{\textbf{e}})$ of (7.43).

#### Corollary 7.4

As a process on $\mathbb {S}^{N-m-1}$, conditional on $\mathrm{\textsf {CP}}(\vec {\textbf{x}}^{\textbf{e}}_{m},\vec {E},\vec {R})$,

7.15 $$\begin{aligned} H_N^{(m)}(\textbf{z}):=\sqrt{\frac{N-m}{N}} \Big ( H_N\big (T^{(m)}(\textbf{z})\big ) - NE_m\Big ) \end{aligned}$$

has the same law as the mixed spherical model with the mixture $\xi _{(m)}$ of (6.6).

Moreover, conditional on $\mathrm{\textsf {CP}}(\vec {\textbf{x}}^{\textbf{e}}_{m},\vec {E},\vec {R})$, if $\textbf{y}=T^{(m)}(\textbf{z})$ and

$$\begin{aligned} \Big (\frac{1}{N}H_{N}(\textbf{y}),\,\frac{\partial _{\textbf{y}-\textbf{x}^{\textbf{e}}_{m}}H_{N}(\textbf{y})}{\Vert \textbf{y}-\textbf{x}^{\textbf{e}}_{m}\Vert ^2}\Big )=\big (E,R\big ), \end{aligned}$$

then

7.16 $$\begin{aligned} \Big (\frac{1}{N-m} H^{(m)}_{N}(\textbf{z}),\,\frac{\partial _{\textbf{z}} H^{(m)}_{N}(\textbf{z})}{\Vert \textbf{z}\Vert ^2}\Big )=\sqrt{\frac{N}{N-m}}\big ((E-E_m),\,(q_{m+1}-q_m)R\big ). \end{aligned}$$

#### Proof

For $$\textbf{z}_1,\textbf{z}_2\in \mathbb {S}^{N-m-1}$$,

$$ \frac{1}{N} \langle T^{(m)}(\textbf{z}_1), T^{(m)}(\textbf{z}_2) \rangle = q_m+\frac{q_{m+1}-q_m}{N-m} \langle \textbf{z}_1, \textbf{z}_2 \rangle \,. $$

Hence, by (7.7) and (7.8), $\mathbb {E}[ H_N^{(m)}(\textbf{z}_1)\,|\, \mathrm{\textsf {CP}}(\vec {\textbf{x}},\vec {E},\vec {R})]=0$ and

$$ \mathbb {E}\big [H_N^{(m)}(\textbf{z}_1)H_N^{(m)}(\textbf{z}_2)\,\big |\,\mathrm{\textsf {CP}}(\vec {\textbf{x}},\vec {E},\vec {R})\big ]=(N-m)\xi _{(m)}\Big ( \frac{1}{N-m}\langle \textbf{z}_1,\textbf{z}_2\rangle \Big ), $$

and our first claim thus follows.

Next, from the definitions it follows that, if $\textbf{y}=T^{(m)}(\textbf{z})$, then $\partial _{\textbf{y}-\textbf{x}^{\textbf{e}}_{m}}H_{N}(\textbf{y})=\sqrt{\frac{N}{N-m}}\partial _{\textbf{z}} H^{(m)}_{N}(\textbf{z})$. Since $\Vert T^{(m)}(\textbf{z})-\textbf{x}_m^\textbf{e}\Vert ^2/\Vert \textbf{z}\Vert ^2=N(q_{m+1}-q_m)/(N-m)$, the second claim follows. □

Building on Corollary 7.4 we proceed to represent the conditional on $\mathrm{\textsf {CP}}(\vec {\textbf{x}}_{m}^\textbf{e},\vec {E},\vec {R})$ joint law of $H^{(m)}_N$ and certain first and second derivatives of it. Specifically, let $(F_i)_{i=1}^{N-m-1}$ be some piecewise smooth orthonormal frame field on the sphere $\mathbb {S}^{N-m-1}$ and for $\textbf{z}\in \mathbb {S}^{N-m-1}$ define $\nabla _{\textrm{sp}}H^{(m)}_{N}(\textbf{z}) := (F_iH^{(m)}_N(\textbf{z}))_{i=1}^{N-m-1}$ and $\nabla _{\textrm{sp}}^{2}H^{(m)}_{N}(\textbf{z}) := (F_iF_jH^{(m)}_N(\textbf{z}))_{i,j=1}^{N-m-1}$. Recall that the *d*-dimensional goe matrix $\textbf{M}$ is a real symmetric matrix whose elements are centered Gaussian variables, independent of each other, up to symmetry, and throughout we shall use the normalization $\mathbb {E}\big [\textbf{M}_{ij}^2\big ]=(1+\delta _{ij})/d$.

#### Corollary 7.5

Let $\textbf{z}\in \mathbb {S}^{N-m-1}$ be an arbitrary point and define the matrix

$$\begin{aligned} \textbf{G}:= \sqrt{\frac{N-m}{(N-m-1)\xi _{(m)}''(1)}}\Bigg ( \nabla _{\textrm{sp}}^{2}H^{(m)}_N(\textbf{z})+\frac{\partial _{\textbf{z}}H^{(m)}_N(\textbf{z})}{\Vert \textbf{z}\Vert ^2}\textbf{I} \Bigg ). \end{aligned}$$

Conditional on $\mathrm{\textsf {CP}}(\vec {\textbf{x}}_{m}^\textbf{e},\vec {E},\vec {R})$, the three random variables

$$ U_N(\textbf{z}):=\Big (\frac{1}{N-m} H^{(m)}_N(\textbf{z}),\frac{\partial _{\textbf{z}}H^{(m)}_N(\textbf{z})}{\Vert \textbf{z}\Vert ^2}\Big ),\qquad \nabla _{\textrm{sp}}H^{(m)}_N(\textbf{z})\qquad \text{ and } \qquad \textbf{G}$$

are independent. Moreover, with $\Sigma _{\xi }$ as defined in (6.3), we have that $U_N(\textbf{z})\sim \textsf {N}(0,(N-m)^{-1}\Sigma _{\xi _{(m)}})$, $\nabla _{\textrm{sp}}H^{(m)}_N(\textbf{z})\sim \textsf {N}(0,\xi _{(m)}'(1)\textbf{I})$ and $\textbf{G}$ is an (N-m-1)-dimensional goe matrix.

#### Proof

In [13, Section 4.1] this corollary is proved for the Hamiltonian of a general spherical model on $\mathbb {S}^{N-1}$. Here we translated this result to our setting on $\mathbb {S}^{N-m-1}$ with the mixture $\xi _{(m)}$, which corresponds to the conditional law of $H_N^{(m)}(\textbf{z})$ by Corollary 7.4. □

### One-level Kac-Rice formulas

For $\vec {\textbf{y}}=(\textbf{y}_1,\ldots ,\textbf{y}_m)\in \mathbb {A}_m$ and $m\le n\le k$, we recall (5.12),

7.17 $$\begin{aligned}&\mathbb {A}_{m,n}(\vec {\textbf{y}}):=\Big \{\vec {\textbf{x}}=(\textbf{x}_i)_{i=1}^{n}\in \mathbb {A}_{n}:\,(\textbf{x}_1,\ldots ,\textbf{x}_m)=(\textbf{y}_1,\ldots ,\textbf{y}_m)\Big \} \end{aligned}$$

7.18 $$\begin{aligned}&\mathscr {C}_{m,n}(\varepsilon ,\vec {\textbf{y}}):=\Big \{\vec {\textbf{x}} \in \mathbb {A}_{m,n}(\vec {\textbf{y}}):\,\exists (\vec {E}, \vec {R})\in \bar{V}(\varepsilon ) \text{ such } \text{ that } \forall m+1\le i\le n,\nonumber \\&\quad \Big (\frac{1}{N}H_{N}(\textbf{x}_i),\,\frac{\partial _{\textbf{x}_i-\textbf{x}_{i-1}}H_{N}(\textbf{x}_i)}{\Vert \textbf{x}_i-\textbf{x}_{i-1}\Vert ^2},\,\bar{\nabla }H_{N}(\textbf{x}_i)\Big )=\big (E_i,R_i,0\big ) \Big \}\,. \end{aligned}$$

By going through the proof of (6.4) in [13], one can verify that multiplying the mixture by a factor that goes to one as $$N\rightarrow \infty $$ or scaling *I* and $I^{\prime }$ by such a factor do not affect the asymptotic result. Hence, from Corollary 7.4, we have that

7.19 $$\begin{aligned} \lim _{N\rightarrow \infty }\frac{1}{N}\log \Big (\mathbb {E}\big [ \#\mathscr {C}_{m,m+1}(\varepsilon ,\vec {\textbf{x}}_m^{\textbf{e}})\,\big |\, \mathrm{\textsf {CP}}(\vec {\textbf{x}}^{\textbf{e}}_{m},\vec {E},\vec {R}) \big ]\Big ) = \sup _{I_m(\varepsilon ,E_m)\times I'_m(\varepsilon )}\Theta _{\xi _{(m)}}(E,R), \end{aligned}$$

for $I'_m(\cdot )$ of (7.4) and $I_m(\varepsilon ,E_m)=[E_{\star }(q_{m+1})-E_m-\varepsilon ,E_{\star }(q_{m+1})-E_m+\varepsilon ]$.

Our next lemma provides the one-level Kac-Rice formula we need for our inductive argument. To this end, we further set $\vec {\textbf{x}}_0^\textbf{e}=\textbf{x}_0^\textbf{e}:=0$, $\mathscr {C}_{0,1}(\varepsilon ,\textbf{x}_0^\textbf{e}):=\mathscr {C}_{1}(\varepsilon )$, $\mathbb {A}_{0,1}:=\mathbb {A}_1$ with $\mathrm{\textsf {CP}}(\vec {\textbf{x}}_0^\textbf{e},\vec {E},\vec {R})$ the entire sample space. We recall that $\mathbb {B}^{N}\subset \mathbb {R}^N$ denotes the ball of radius $\sqrt{N r}$ for some $1 \le r < 1 + \varepsilon _\xi$.

#### Lemma 7.6

Fix $0\le m\le k-1$ and a non-random $\bar{w}_N(\textbf{x},\varphi ): \sqrt{q_{m+1}}\,\mathbb {S}^{N-1}\times C^\infty (\mathbb {B}^{N}) \rightarrow \mathbb {R}$, assuming as in Theorem 1.4, that $\bar{w}_N$ is rotationally invariant and that for any $\varphi ,\psi _{i}\in C^\infty (\mathbb {B}^{N})$ and d≥1,

7.20 $$\begin{aligned} \lim _{t_{i}\rightarrow 0}\bar{w}_{N}\Big (\textbf{x},\varphi +\sum _{i\le d}t_{i}\psi _{i}\Big )=\bar{w}_{N}(\textbf{x},\varphi ). \end{aligned}$$

Suppose further that for any $(\vec {E},\vec {R})\in \bar{V}(\varepsilon )$, the function $w_{N}(\textbf{x}):=\bar{w}_{N}(\textbf{x},H_{N}(\cdot ))$ is a.s. continuous on^8^$\sqrt{q_{m+1}}\,\mathbb {S}^{N-1}$ conditionally on $\mathrm{\textsf {CP}}(\vec {\textbf{x}}_m^\textbf{e},\vec {E},\vec {R})$ and $\mathbb {E}[w_{N}(\textbf{x})^{2}|\mathrm{\textsf {CP}}(\vec {\textbf{x}}_m^\textbf{e},\vec {E},\vec {R})]<\infty$ for any $\textbf{x}$ and *N*. Let $I\subset \mathbb {R}$ be an open set and $\bar{I}$ its closure. Then, for $I_m$ and $I'_m$ of (7.3) and (7.4),

$$\begin{aligned}&\varlimsup _{N\rightarrow \infty }\frac{1}{N}\log \Big (\sup _{\bar{V}(\varepsilon )} \mathbb {E}\Big [ \sum _{\vec {\textbf{x}}\in \mathscr {C}_{m,m+1}(\varepsilon ,\vec {\textbf{x}}_m^\textbf{e})} 1\!\!1\big \{w_N(\textbf{x}_{m+1})\in I \big \} \,\Big |\,\mathrm{\textsf {CP}}(\vec {\textbf{x}}_m^\textbf{e},\vec {E},\vec {R}) \Big ]\Big )\\&\le \sup _{(E,R) \in I_m(\varepsilon )\times I'_m(\varepsilon )}\Theta _{\xi _{(m)}}(E,R) +\varlimsup _{N\rightarrow \infty }\frac{1}{N}\log \Big (\sup _{\bar{V}(\varepsilon )}\mathbb {P}\Big [ w_N(\textbf{x}_{m+1}^{\textbf{e}})\in \bar{I} \,\\&\quad \Big |\,\mathrm{\textsf {CP}}(\vec {\textbf{x}}_{m+1}^\textbf{e},\vec {E},\vec {R}) \Big ]\Big ). \end{aligned}$$

#### Proof

In the pure case of $\xi (t)=ct^{p}$ with m=0 the random variable $\partial _{\textbf{x}}H_{N}(\textbf{x})=\partial _{\textbf{x}-\textbf{x}_0^{\textbf{e}}}H_{N}(\textbf{x})$ is measurable wrt $H_{N}(\textbf{x})$, while in the mixed case (that is, any other mixture ξ(t)), or if m≥1 the law of $(H_{N}(\textbf{x}),\partial _{\textbf{x}-\textbf{x}_m^{\textbf{e}}}H_{N}(\textbf{x}))$ is non-degenerate for $\textbf{x}\perp \textbf{x}_m^{\textbf{e}}$. We assume hereafter either that the mixture ξ(t) is not pure or that m≥1. In the case that ξ(t) is pure and m=0 some modifications to the proof are needed. The main one being that the terms involving $\partial _{\textbf{z}} H^{(m)}_{N}(\textbf{z})$ should be removed from the definitions of $U_N(\textbf{z})$, $V_N(\textbf{z})$ and $\mathcal {H}_N(\textbf{z})$ below and, since $\partial _{\textbf{z}} H^{(m)}_{N}(\textbf{z})/\Vert \textbf{z}\Vert ^2=\alpha _{p,q_1} H^{(m)}_{N}(\textbf{z})/(N-m)$ for $\alpha _{p,q}=\sqrt{q}p=qR_{\star }(q)/E_{\star }(q)$, the set $I_1\times I_2$ should be replaced by $\bar{I}_1:=\{E\in I_1, \alpha _{p,q_1}E\in I_2\}$. Noting that the degeneracy only simplifies the proof and that $\mathrm{\textsf {CP}}(\vec {\textbf{x}}_m^\textbf{e},\vec {E},\vec {R})=\Omega _0$ for m=0 where $\Omega _0$ is the whole sample space, other modifications in this case are straightforward and left to the reader.

Throughout the proof, we denote expectation conditional on $\mathrm{\textsf {CP}}(\vec {\textbf{x}}_m^\textbf{e},\vec {E},\vec {R})$ for fixed values of $(E_i,R_i)_{i\le m}$ by $\mathbb {E}_0$. Recalling the definition (7.15) of $H^{(m)}_N(\textbf{z})$, we have from Corollary 7.4 that

7.21 $$\begin{aligned}&\mathbb {E}_0\Big [ \sum _{\vec {\textbf{x}}\in \mathscr {C}_{m,m+1}(\varepsilon ,\vec {\textbf{x}}_m^\textbf{e})} \!\!\!\!\!\!\!\! 1\!\!1\big \{w_N(\textbf{x}_{m+1}) \in I\big \} \Big ]\nonumber \\&\quad =\mathbb {E}_0\Big [ \sum _{\begin{array}{c} \textbf{z}\in \mathbb {S}^{N-m-1}:\\ \nabla _{\textrm{sp}}H_{N}^{(m)}(\textbf{z})=0 \end{array}} \!\!\!\! 1\!\!1\big \{ w_N(T^{(m)}(\textbf{z}))\in I,\, U_N(\textbf{z})\in I_1\times I_2 \big \} \Big ], \end{aligned}$$

where

7.22 $$\begin{aligned} \begin{aligned} U_N(\textbf{z})&:=\Big (\frac{1}{N-m} H^{(m)}_{N}(\textbf{z}),\,\frac{\partial _{\textbf{z}} H^{(m)}_{N}(\textbf{z})}{\Vert \textbf{z}\Vert ^2}\Big ),\\I_1&:=\sqrt{\frac{N}{N-m}}\big (E_{\star }(q_{m+1})-E_m-\varepsilon ,E_{\star }(q_{m+1})-E_m+\varepsilon \big ),\\I_2&:=\sqrt{\frac{N}{N-m}}(q_{m+1}-q_m)\big (R_{\star }(q_{m+1})-\varepsilon ,R_{\star }(q_{m+1})+\varepsilon \big ). \end{aligned} \end{aligned}$$

We thus proceed to apply the Kac-Rice formula in order to bound the expectation in (7.21). To this end, fixing ε>0 we set the process $w_{N}^{\varepsilon }(\textbf{x})=w_{N}(\textbf{x})+\varepsilon Z$ where $Z\sim \textsf {N}(0,1)$ is independent of all other random variables. We further define the vector-valued processes

$$\begin{aligned} V_{N}(\textbf{z})&:=\left( H^{(m)}_{N}(\textbf{z}),\partial _{\textbf{z}} H^{(m)}_{N}(\textbf{z}),\nabla _{\textrm{sp}}H^{(m)}_{N}(\textbf{z}),\text{ vec }(\nabla _{\textrm{sp}}^{2}H^{(m)}_{N}(\textbf{z})),w_{N}^{\varepsilon }(T^{(m)}(\textbf{z}))\right) , \\\mathcal {H}_{N}(\textbf{z})&:=\left( H^{(m)}_{N}(\textbf{z}),\partial _{\textbf{z}} H^{(m)}_{N}(\textbf{z}),\nabla _{\textrm{sp}}H^{(m)}_{N}(\textbf{z}),\text{ vec }(\nabla _{\textrm{sp}}^{2}H^{(m)}_{N}(\textbf{z}))\right) , \end{aligned}$$

where $\text{ vec }(\nabla _{\textrm{sp}}^{2}H^{(m)}_{N}(\textbf{z}))$ means the vectorization of the on-and-above elements of $\nabla _{\textrm{sp}}^{2}H^{(m)}_{N}(\textbf{z})$. Note that by rotational invariance, the laws of $V_{N}(\textbf{z})$ and $\mathcal {H}_{N}(\textbf{z})$ do not depend on $\textbf{z}\in \mathbb {S}^{N-m-1}$.

Set ${\bar{N}}:=N-m-1$ and $D=2+{\bar{N}}+\frac{1}{2}{\bar{N}}({\bar{N}}+1)$. By Corollary 7.5, conditional on $\mathrm{\textsf {CP}}(\vec {\textbf{x}}_m^\textbf{e},\vec {E},\vec {R})$, per fixed $\textbf{z}$, the *D*-dimensional vector $\mathcal {H}_{N}(\textbf{z})$ has non-degenerate Gaussian density $\varphi _{\mathcal {H}_{N}(\textbf{z})}(\cdot )$ on $\mathbb {R}^{D}$. Since $H_{N}$ is a Gaussian process, conditional on $\mathrm{\textsf {CP}}(\vec {\textbf{x}}_m^\textbf{e},\vec {E},\vec {R})$ and on $\mathcal {H}_{N}(\textbf{z})=\underline{t}=(t_{1},\ldots ,t_{D})$, the process $H_{N}(\cdot )$ has the same law as

$$ \mathbb {E}_0\left[ H_{N}(\cdot )\,|\,\mathcal {H}_{N}(\textbf{z})=\underline{t}\right] +Y_{N}(\cdot )=\sum _{i=1}^{D}t_{i}\psi _{N}^{(i)}(\cdot )+Y_{N}(\cdot ), $$

for some deterministic functions $\psi _{N}^{(i)}\in C^\infty (\mathbb {B}^{N})$, where $Y_{N}\in C^\infty (\mathbb {B}^{N})$ is a Gaussian process whose law is that of $H_N$ given that $\mathcal {H}_{N}(\textbf{z})=0$. In particular, conditionally on $\mathrm{\textsf {CP}}(\vec {\textbf{x}}_m^\textbf{e},\vec {E},\vec {R})$, the vector $V_{N}(\textbf{z})$ has at any $(\underline{t},s)$ the density $p_{\varepsilon }(s|\underline{t}) \varphi _{\mathcal {H}_{N}(\textbf{z})}(\underline{t})$, where

$$\begin{aligned} \begin{aligned} p_{\varepsilon }(s|\underline{t})&:=\frac{1}{\sqrt{2\pi \varepsilon ^{2}}}\mathbb {E}_0\bigg (\exp \Big [-\frac{1}{2\varepsilon ^{2}}\big (s-\bar{w}_{N}\big (T^{(m)}(\textbf{z}),H_{N}(\cdot )\big )\big )^{2}\Big ] \,\Big |\, \mathcal {H}_N(\textbf{z})=\underline{t}\bigg ) \\&=\frac{1}{\sqrt{2\pi \varepsilon ^{2}}}\mathbb {E}_0\exp \Big [-\frac{1}{2\varepsilon ^{2}}\Big (s-\bar{w}_{N}\big (T^{(m)}(\textbf{z}),\sum _{i=1}^{D}t_{i}\psi _{N}^{(i)}(\cdot )+Y_{N}(\cdot )\big )\Big )^{2}\Big ]. \end{aligned} \end{aligned}$$

From the bounded convergence theorem and (7.20), the density $p_{\varepsilon }(s|\underline{t})$ is continuous in $(\underline{t},s)$, and hence, by the preceding, conditionally, $V_{N}(\textbf{z})$ has a strictly positive, continuous density. This implies in turn, by the same reasoning as in [23, Proposition 5.1], that the rhs of (7.21) is bounded above by $K(\Omega _0)$ where $\Omega _0$ is the whole sample space and

$$\begin{aligned} K(\cdot )&:=C_{N,m} \mathbb {E}_0\bigg [ \left| \det \left( \nabla _{\textrm{sp}}^{2}H^{(m)}_{N}(\textbf{z})\right) \right| 1\!\!1\{\cdot \}1\!\!1\big \{ w_N(T^{(m)}(\textbf{z}))\in \bar{I},\, U_N(\textbf{z})\in I_1 \\&\times I_2 \big \} \, \bigg | \, \nabla _{\textrm{sp}}H^{(m)}_{N}(\textbf{z})=0 \bigg ], \end{aligned}$$

with $\textbf{z}\in \mathbb {S}^{\bar{N}}$ an arbitrary point,

7.23 $$\begin{aligned} C_{N,m}:=(\sqrt{ N-m})^{N-m-1} \frac{2\pi ^{(N-m)/2}}{\Gamma \left( (N-m)/2\right) } \widetilde{\varphi }_{N,m} (0) \end{aligned}$$

and $\widetilde{\varphi }_{N,m}(\cdot )$ denotes the Gaussian density of $\nabla _{\textrm{sp}}H^{(m)}_{N}(\textbf{z})$ conditional on $\mathrm{\textsf {CP}}(\vec {\textbf{x}}_m^\textbf{e},\vec {E},\vec {R})$, which by Corollary 7.5 is merely the density of a $\textsf {N}(0,\xi _{(m)}'(1)\textbf{I}_{{\bar{N}}})$ vector. To bound $K(\Omega _0)=K(\mathcal {E})+K(\mathcal {E}^c)$ we will bound each of the two summands for $\mathcal {E}$ to be determined below.

Now, by Corollary 7.5 the density of $U_N(\textbf{z})$ conditional on $\mathrm{\textsf {CP}}(\vec {\textbf{x}}_{m}^\textbf{e},\vec {E},\vec {R})$ and $\nabla _{\textrm{sp}}H^{(m)}_{N}(\textbf{z})=0$, is merely the density $\varphi _{N,m}$ of $\textsf {N}(0,\frac{1}{N-m}\Sigma _{\xi _{(m)}})$, while upon conditioning also on $U_N(\textbf{z})=(E,R)$, the matrix $\nabla _{\textrm{sp}}^{2}H^{(m)}_{N}(\textbf{z})$ has the law of $b_m \textbf{G}- R \,\textbf{I}$ where $b_m^2 := \xi _{(m)}''(1)$ and $\textbf{G}$ is an ${\bar{N}}$-dimensional goe matrix, scaled by the factor $\sqrt{\frac{{\bar{N}}}{{\bar{N}}+1}}$. To bound $K(\Omega _0)$, we set

7.24 $$\begin{aligned} Z_{{\bar{N}},R}:=\frac{1}{{\bar{N}}}\log \big |\det (\textbf{G}-(R/b_m)\textbf{I})\big | = \int \log |\lambda - R/b_m| d\mu _{{\bar{N}}}(\lambda ) \,, \end{aligned}$$

where $\mu _{{\bar{N}}}$ is the empirical measure of eigenvalues of $\textbf{G}$. Considering for t>0 the event $\mathcal {E}:=\big \{ Z_{{\bar{N}},R} \le \Omega (R/b_m) + t \big \}$ and decomposing according to the value of $U_N(\textbf{z})$, we see that

$$ K(\mathcal {E}) \le C_{N,m} \int _{I_1\times I_2} \Gamma _{{\bar{N}},R}(t) Q_{E,R} (\bar{I}) \varphi _{N,m}(E,R)dE dR \,, $$

where for $\Omega (\cdot )$ of (6.2),

7.25 $$\begin{aligned} \Gamma _{{\bar{N}},R}(t)&: = b_m^{{\bar{N}}} \exp \big ( {\bar{N}}(\Omega (R/b_m)+t)\big ) \qquad \text {and} \nonumber \\ Q_{E,R} (\bar{I})&:= \mathbb {P}_0\Big [ w_N(T^{(m)}(\textbf{z}))\in \bar{I} \, \Big | \, \nabla _{\textrm{sp}}H^{(m)}_{N}(\textbf{z})=0,\, U_N(\textbf{z})=(E,R) \Big ]\,. \end{aligned}$$

Similarly,

$$\begin{aligned} K(\mathcal {E}^c) \le C_{N,m} \int _{I_1 \times I_2} b_m^{{\bar{N}}} \mathbb {E}\Big [ e^{{\bar{N}}Z_{{\bar{N}},R}}\cdot 1\!\!1\{Z_{{\bar{N}},R} > \Omega (R/b_m)+ t \} \Big ] \varphi _{N,m}(E,R)dE dR \,, \end{aligned}$$

from which we deduce by Cauchy-Schwarz, that $K(\mathcal {E}^c) \le C_{N,m} A(t) |I_1||I_2|$, for

$$\begin{aligned} A (t)^2 := \sup _{R\in I_2} \Big \{ b_m^{2 {\bar{N}}} \mathbb {E}\Big [ e^{2 {\bar{N}}Z_{{\bar{N}},R}} \Big ] \Big \} \sup _{R\in I_2} \Big \{ \mathbb {P}\big ( Z_{{\bar{N}},R} > \Omega (R/b_m) + t \big ) \Big \} \,. \end{aligned}$$

Denoting by $\lambda _i$ the eigenvalues of $\textbf{G}-(R/b_m)\textbf{I}$ and setting $\bar{\lambda }:=\max _i |\lambda _i|$, we have that $Z_{{\bar{N}},R} \le \log \bar{\lambda }$. Recall from [10, Lemma 6.3], that for some universal constant $x_0$ and any $x \ge x_0$

7.26 $$\begin{aligned} \mathbb {P}\big ( |\bar{\lambda }| > x + |R|/b_m \big ) \le e^{-{\bar{N}}x^2/9} \qquad \forall {\bar{N}}, R \,, \end{aligned}$$

hence,

7.27 $$\begin{aligned} \limsup _{N \rightarrow \infty } \sup _{R \in I_2} \frac{1}{{\bar{N}}} \log \Big ( b_m^{2{\bar{N}}} \mathbb {E}\big [ e^{2 {\bar{N}}Z_{{\bar{N}},R}} \big ] \Big ) < \infty \,. \end{aligned}$$

Further, fixing t>0, define for δ>0 the bounded, continuous truncated functions

$$ \log _\delta (t) := \log \Big ( ( |t| \vee \delta ) \wedge (1/\delta ) \Big )\,, $$

whereby

7.28 $$\begin{aligned}&\mathbb {P}\big ( Z_{{\bar{N}},R}> \Omega (R/b_m) + t \big )\le \mathbb {P}\Big ( \int \log _\delta (\lambda - R/b_m) d\mu _{{\bar{N}}}(\lambda )> \Omega (R/b_m) \nonumber \\&\quad + t \Big )+\mathbb {P}\big (\bar{\lambda } > \frac{1}{\delta }\big ) \,. \end{aligned}$$

Note that there exists a number $\delta _o(t)>0$ depending on *t*, such that for any $\delta \le \delta _o(t)$ and all $R\in I_2$,

$$ \frac{1}{2\pi } \int _{-2}^{2} \log _{\delta } (\lambda -R/b_m) \sqrt{4-\lambda ^2}d\lambda - \Omega (R/b_m) \le \frac{t}{2} \,, $$

in which case, by the large deviation principle at rate ${\bar{N}}^2$ for $\mu _{\bar{N}}$ (see [12, Theorem 2.1.1]), we bound the first term on the rhs of (7.28) by $\exp (-C_\delta {\bar{N}}^2)$ for some $C_\delta >0$, all ${\bar{N}}$ large enough and any $R \in I_2$. Bounding the second term in (7.28) by (7.26), then taking $N \rightarrow \infty$ followed by $\delta \rightarrow 0$ we conclude that for any t>0,

$$\begin{aligned} \lim _{N \rightarrow \infty } \sup _{R \in I_2} \frac{1}{{\bar{N}}} \log \mathbb {P}\big ( Z_{{\bar{N}},R} > \Omega (R/b_m) + t \big )&= -\infty \,. \end{aligned}$$

This, together with (7.27) implies in turn that $A(t_N)\le e^{-C_N N}$ for some $t_N \rightarrow 0$ and $C_N \rightarrow \infty$.

Assume wlog that $\textbf{z}=(T^{(m)})^{-1}(\textbf{x}_{m+1}^\textbf{e})$ and set $(E_{m+1},R_{m+1})$ by the inverse of (7.16), namely

7.29 $$\begin{aligned} E_{m+1}:= \sqrt{\frac{N-m}{N}}E+E_m,\qquad R_{m+1}:= \sqrt{\frac{N-m}{N}}\frac{1}{q_{m+1}-q_m}R, \end{aligned}$$

to get that

$$ Q_{E,R}(\bar{I}) = \mathbb {P}\left( w_N(\textbf{x}_{m+1}^{\textbf{e}})\in \bar{I} \, \Big | \, \mathrm{\textsf {CP}}(\vec {\textbf{x}}_{m+1}^\textbf{e},\vec {E},\vec {R}) \right) \,. $$

Consequently, subject to (7.29), we have that for some $t_N \rightarrow 0$ and $C_N \rightarrow \infty$,

7.30 $$\begin{aligned}&\mathbb {E}\Big [ \sum _{\vec {\textbf{x}}\in \mathscr {C}_{m,m+1}(\varepsilon ,\vec {\textbf{x}}_m^\textbf{e})} 1\!\!1\big \{w_N(\textbf{x}_{m+1})\in I \big \} \,\Big |\,\mathrm{\textsf {CP}}(\vec {\textbf{x}}_m^\textbf{e},\vec {E},\vec {R}) \Big ] \le C_{N,m} \Big [ e^{-C_N N} \nonumber \\&\quad + \int _{I_1\times I_2} \Gamma _{{\bar{N}},R}(t_N) \mathbb {P}\left( w_N(\textbf{x}_{m+1}^{\textbf{e}})\in \bar{I} \, \Big | \, \mathrm{\textsf {CP}}(\vec {\textbf{x}}_{m+1}^\textbf{e},\vec {E},\vec {R}) \right) \varphi _{N,m}(E,R)dEdR \Big ]\,. \end{aligned}$$

By definition, the mapping (7.29) is a bijection between $(E,R) \in I_1\times I_2$ and

$$ \big \{(E_{m+1},R_{m+1}):\, |E_{m+1}-E_{\star }(q_{m+1})|<\varepsilon ,\,|R_{m+1}-R_{\star }(q_{m+1})|<\varepsilon \big \}\,. $$

Thus, if $(E_i,R_i)_{i \le m} \in \bar{V}(\varepsilon )$ and $(E,R) \in I_1 \times I_2$, then also $(E_i,R_i)_{i \le m+1} \in \bar{V}(\varepsilon )$ (more precisely, in both cases, $(E_i,R_i)_i$ can be completed to a length *k* vector in $\bar{V}(\varepsilon )$). Hence,

7.31 $$\begin{aligned}&\varlimsup _{N\rightarrow \infty } \frac{1}{N}\log \Big (\sup _{\bar{V}(\varepsilon )} \mathbb {E}\big [ \sum _{\vec {\textbf{x}}\in \mathscr {C}_{m,m+1}(\varepsilon ,\vec {\textbf{x}}_m^\textbf{e})} 1\!\!1\big \{w_N(\textbf{x}_{m+1})\in I \big \} \,\Big |\,\mathrm{\textsf {CP}}(\vec {\textbf{x}}_m^\textbf{e},\vec {E},\vec {R}) \big ]\Big ) \nonumber \\&\quad \le \varlimsup _{N\rightarrow \infty } \sup _{E_m}\sup _{I_1\times I_2} \frac{1}{N}\log \Big ( C_{N,m} \Gamma _{{\bar{N}},R}(0) \varphi _{N,m}(E,R) \Big ) \nonumber \\&\quad + \varlimsup _{N\rightarrow \infty }\frac{1}{N}\log \Big (\sup _{\bar{V}(\varepsilon )}\mathbb {P}\Big [ w_N(\textbf{x}_{m+1}^{\textbf{e}})\in \bar{I} \,\Big |\,\mathrm{\textsf {CP}}(\vec {\textbf{x}}_{m+1}^\textbf{e},\vec {E},\vec {R}) \Big ]\Big )=:A+B\,, \end{aligned}$$

where the supremum in (7.31) is over $E_m$ such that $|E_m - E_{\star }(q_m)|<\varepsilon$. Noting that *B* is the same term as in the bound stated in the lemma, it remains to show that

7.32 $$\begin{aligned} A \le \sup _{(E,R) \in I_m(\varepsilon )\times I'_m(\varepsilon )}\Theta _{\xi _{(m)}}(E,R)\,. \end{aligned}$$

The function whose supremum is taken in (7.31) only depends on (*E*, *R*). The dependence on $E_m$ is through the set $I_1$ defined in (7.22). Instead of the double supremum, we can therefore take in (7.31) a supremum over (*E*, *R*) in

$$ J_N(\varepsilon ):=\sqrt{\frac{N}{N-m}}\Big (E_{\star }(q_{m+1})-E_{\star }(q_{m})-2\varepsilon ,E_{\star }(q_{m+1})-E_{\star }(q_{m})+2\varepsilon \Big )\times I_2\,. $$

For $I_m(\varepsilon )$ and $I'_m(\varepsilon )$ of (7.3), the decreasing sequence $J_{N+1}(\varepsilon )\subset J_N(\varepsilon )$ converges to

$$ \lim _{N \rightarrow \infty } J_N(\varepsilon ):=\bigcap _{N>m} J_N(\varepsilon ) = I_m(\varepsilon )\times I'_m(\varepsilon )\,. $$

Finally, for $C_{N,m}$, $\Gamma _{{\bar{N}},R}(0)$, $\Sigma _{\xi _{(m)}}$ of (7.23), (7.25) and (6.3), respectively, with

$$\begin{aligned} \widetilde{\varphi }_{N,m}(0)&= (2\pi \xi _{(m)}'(1))^{-\frac{N-m-1}{2}},\quad \varphi _{N,m}(E,R)\\&=\frac{N-m}{2\pi \sqrt{\det (\Sigma _{\xi _{(m)}})}}\exp \Big (-\frac{N-m}{2}{(E,R)\Sigma _{\xi _{(m)}}^{-1}(E,R)^T}\Big ), \end{aligned}$$

we confirm via Stirling’s approximation (for Γ((N-m)/2) in (7.23)), that uniformly on compacts,

$$ \lim _{N\rightarrow \infty } \frac{1}{N} \log \Big ( C_{N,m} \Gamma _{{\bar{N}},R}(0) \varphi _{N,m}(E,R) \Big ) = \Theta _{\xi _{(m)}}(E,R) \,, $$

and thereby conclude the proof of (7.32), and thus the lemma. □

Building on Lemma 7.6, we further control the corresponding conditional first moment of $w_N(\cdot )$.

#### Corollary 7.7

Assume in the setting of Lemma 7.6, that in addition, $w_N(\textbf{x}) \in [0, e^{NM}]$ for finite *M*, any $\textbf{x}$ and *N*. Then,

7.33 $$\begin{aligned}&\varlimsup _{N\rightarrow \infty }\frac{1}{N}\log \Big (\sup _{\bar{V}(\varepsilon )} \mathbb {E}\Big [ \sum _{\vec {\textbf{x}}\in \mathscr {C}_{m,m+1}(\varepsilon ,\vec {\textbf{x}}_m^\textbf{e})} w_N(\textbf{x}_{m+1}) \,\Big |\,\mathrm{\textsf {CP}}(\vec {\textbf{x}}_m^\textbf{e},\vec {E},\vec {R}) \Big ]\Big ) \nonumber \\&\le \sup _{I_m(\varepsilon )\times I'_m(\varepsilon )}\Theta _{\xi _{(m)}}(E,R) + \varlimsup _{N\rightarrow \infty }\frac{1}{N}\log \Big (\sup _{\bar{V}(\varepsilon )}\mathbb {E}\Big [ w_N(\textbf{x}_{m+1}^{\textbf{e}}) \,\Big |\,\mathrm{\textsf {CP}}(\vec {\textbf{x}}_{m+1}^\textbf{e},\vec {E},\vec {R}) \Big ]\Big ). \end{aligned}$$

#### Proof

We fix the values of $(E_i,R_i)_{i\le m}$ and cover $[0,e^{MN}]$ by the union of open intervals $B_1,\ldots ,B_\kappa$ (allowing $B_i$ and κ to depend on *N*), with $b_i:=\sup B_i$, such that

$$\begin{aligned}&w \le \sum _{i=1}^\kappa b_i 1\!\!1_{B_i} (w) \le \sum _{i=1}^\kappa b_i 1\!\!1_{\bar{B}_i}(w) {\mathop {\le }\limits ^{(\star )}} 5 w + e^{-N^2} \quad and \quad \sum _{i=1}^\kappa b_i {\mathop {\le }\limits ^{(\star \star )}} 6 e^{M N} \\&\quad \forall w \in [0,e^{MN}] \,. \end{aligned}$$

Indeed, the only non-trivial inequalities here are (⋆) and $(\star \star )$, both of which are satisfied for example by $B_1=(-e^{-N^2},e^{-N^2})$ and $B_i = (2^{i-2.5} e^{-N^2},2^{i-1} e^{-N^2})$, i≥2. Utilizing the preceding bounds, we get from (7.30) for $I=B_i$, $1 \le i \le \kappa$, that for some $t_N \rightarrow 0$ and $C_N \rightarrow \infty$, under the relation (7.29),

7.34 $$\begin{aligned} \mathbb {E}\Big [ \sum _{\vec {\textbf{x}}\in \mathscr {C}_{m,m+1}(\varepsilon ,\vec {\textbf{x}}_m^\textbf{e})}&\!\!\!\! w_N(\textbf{x}_{m+1}) \,\Big |\,\mathrm{\textsf {CP}}(\vec {\textbf{x}}_m^\textbf{e},\vec {E},\vec {R}) \Big ] \le C_{N,m} \bigg \{ 6 e^{M N} e^{-C_N N} \nonumber \\&+ \int _{I_1\times I_2} \Gamma _{\bar{N},R}(t_N) \Big ( e^{-N^2} \nonumber \\&+ 5 \mathbb {E}\big [ w_N(\textbf{x}_{m+1}^{\textbf{e}}) \, \big | \, \mathrm{\textsf {CP}}(\vec {\textbf{x}}_{m+1}^\textbf{e},\vec {E},\vec {R}) \big ] \Big ) \varphi _{N,m}(E,R)dEdR \bigg \}\,, \end{aligned}$$

and (7.33) follows by the same argument as after (7.30). For later use, we note that the bound (7.34) holds uniformly for any $w_N$ as in the corollary (since $C_N$ depends only on $A(t_N)$ whose definition does not involve $w_N$). □

### Proof of Proposition 7.1

To prove Proposition 7.1, we set for m≤n and $\vec {\textbf{y}}\in \mathbb {A}_m$,

7.35 $$\begin{aligned} \alpha _{m,n}^{\varepsilon }(\vec {\textbf{y}}):=\sum _{\vec {\textbf{x}}\in \mathscr {C}_{m,n}(\varepsilon ,\vec {\textbf{y}})} \alpha _n(\vec {\textbf{x}})\,, \end{aligned}$$

where $\mathscr {C}_{m,n}(\varepsilon ,\vec {\textbf{y}})$ is defined in (7.18). With $\vec {\textbf{x}}_0^{\textbf{e}}=0$, $\mathscr {C}_{0,n}(\varepsilon ,0)=\mathscr {C}_{n}(\varepsilon )$ and $\mathrm{\textsf {CP}}(\vec {\textbf{x}}_{0}^\textbf{e},\vec {E},\vec {R})$ the entire sample space, the lhs of (7.5) is merely

$$\begin{aligned}&\varlimsup _{N\rightarrow \infty }\frac{1}{N} \log \mathbb {E}\Big [ \alpha _{0,n}^{\varepsilon }(\vec {\textbf{x}}_0^{\textbf{e}}) \wedge e^{MN} \,\big |\, \mathrm{\textsf {CP}}(\vec {\textbf{x}}_{0}^\textbf{e},\vec {E},\vec {R}) \Big ] \,. \end{aligned}$$

Further, as $\alpha _{n,n}^{\varepsilon }(\cdot )=\alpha _n(\cdot )$, the proposition follows upon showing that, for any $0\le m\le n-1$,

7.36 $$\begin{aligned} \begin{aligned}&\varlimsup _{N\rightarrow \infty }\frac{1}{N}\log \Big (\sup _{\bar{V}(\varepsilon )} \mathbb {E}\Big [ \alpha _{m,n}^{\varepsilon }(\vec {\textbf{x}}_m^{\textbf{e}}) \wedge e^{MN} \,\big |\,\mathrm{\textsf {CP}}(\vec {\textbf{x}}_{m}^\textbf{e},\vec {E},\vec {R}) \Big ]\Big )\\&\le \sup _{I_m(\varepsilon )\times I'_m(\varepsilon )}\Theta _{\xi _{(m)}}(E,R) + \varlimsup _{N\rightarrow \infty }\frac{1}{N}\log \\&\quad \Big (\sup _{\bar{V}(\varepsilon )}\mathbb {E}\Big [ \alpha _{m+1,n}^{\varepsilon }(\vec {\textbf{x}}_{m+1}^{\textbf{e}}) \wedge e^{MN} \,\big |\,\mathrm{\textsf {CP}}(\vec {\textbf{x}}_{m+1}^\textbf{e},\vec {E},\vec {R}) \Big ]\Big ). \end{aligned} \end{aligned}$$

To this end, we plan to apply the one-level Kac-Rice formula of Corollary 7.7. However, as we move $\vec {\textbf{y}}$, new points may ‘enter’ $\mathscr {C}_{m,n}(\varepsilon ,\vec {\textbf{y}})$ while others may ‘disappear’ from it. With $\alpha _{m,n}^{\varepsilon }(\vec {\textbf{y}})$ discontinuous at those $\vec {\textbf{y}}$ where this happens, some preliminary work is needed to approximate $\alpha _{m,n}^{\varepsilon }(\cdot )$, m≤n by continuous functions of the form

$$ \alpha _{m,n}^{\varepsilon ,\delta }(\vec {\textbf{y}}):=\sum _{\vec {\textbf{x}}\in \mathscr {C}_{m,n}(\varepsilon ,\vec {\textbf{y}})} \Psi ^{\varepsilon ,\delta }_{m,n}(\vec {\textbf{x}}) \alpha _n(\vec {\textbf{x}})\,. $$

Specifically, to define $\bar{\nabla }H_{N}(\textbf{x}_i):=M_{i}\nabla H_{N}(\textbf{x}_i)$ we used an arbitrary matrix $M_i=M_i(\vec {\textbf{x}})\in \mathbb {R}^{N-i\times N}$ whose rows form an orthonormal basis of $\big (\textrm{sp}\{\textbf{x}_1,\ldots ,\textbf{x}_i\}\big )^{\perp }$. Using the same matrix, for any $i\le m\le k$ and $\vec {\textbf{x}}=(\textbf{x}_1,\ldots ,\textbf{x}_m)$, define $\bar{\nabla }^{2}H_{N}(\textbf{x}_i):=M_{i}\nabla ^2 H_{N}(\textbf{x}_i)M_i^{\top }$ and

7.37 $$\begin{aligned} \eta _i(\vec {\textbf{x}}):=\min _j \bigg |\lambda _j(\bar{\nabla }^{2}H_{N}(\textbf{x}_i))-\frac{\partial _{\textbf{x}_i-\textbf{x}_{i-1}}H_{N}(\textbf{x}_i)}{\Vert \textbf{x}_i-\textbf{x}_{i-1}\Vert ^2}\bigg |, \end{aligned}$$

where $\lambda _j(A)$ are the eigenvalues of a matrix *A*. For any $\frac{\varepsilon }{2}>\delta >0$, let $\bar{h}_\delta (t)=1 \wedge (\frac{t}{\delta }-1)_+$ and set

$$\begin{aligned} \Psi ^{\varepsilon ,\delta }_{m,n}(\vec {\textbf{x}})&:=\prod _{i=m+1}^n\bar{h}_\delta \Big (\varepsilon -\big | \frac{1}{N}H_N(\textbf{x}_i)-E_{\star }(q_i)\big |\Big ) \bar{h}_\delta \Big (\varepsilon \\&- \big | \frac{\partial _{\textbf{x}_i-\textbf{x}_{i-1}}H_{N}(\textbf{x}_i)}{\Vert \textbf{x}_i-\textbf{x}_{i-1}\Vert ^2}-R_{\star }(q_i)\big | \Big ) \bar{h}_\delta \big (\eta _i(\vec {\textbf{x}})\big ). \end{aligned}$$

Note that $\bar{h}_\delta (t) \nearrow 1\!\!1_{(0,\infty )}(t)$ as $\delta \downarrow 0$, and therefore $\Psi ^{\varepsilon ,\delta }_{m,n}(\vec {\textbf{x}}) \nearrow 1$ for any $\vec {\textbf{x}} \in \mathscr {C}_{m,n}(\varepsilon ,\vec {\textbf{x}}_m^{\textbf{e}})$ with $\min _{m<j \le n} \{ \eta _j(\vec {\textbf{x}})\} > 0$. Recall that any such $\vec {\textbf{x}}$ is in $\mathbb {A}_{m,n}(\vec {\textbf{x}}_m^{\textbf{e}})$ and must satisfy the condition as in (7.18) at any m<i≤n. In particular, for such $\vec {\textbf{x}}$ we have that $\bar{\nabla }H_{N}(\textbf{x}_i)=0$ for all i∈(m,n]. The same applies if $\vec {\textbf{x}}\in \mathscr {C}_{m+1,n}(\varepsilon ,\vec {\textbf{y}})$ for some $\vec {\textbf{y}}\in \mathscr {C}_{m,m+1}(\varepsilon ,\vec {\textbf{x}}_m^{\textbf{e}})$, so from Lemma 7.8 we deduce that conditionally on $\mathrm{\textsf {CP}}(\vec {\textbf{x}}_m^{\textbf{e}},\vec {E},\vec {R})$, almost surely, as $\delta \downarrow 0$,

$$ \sum _{\begin{array}{c} \vec {\textbf{x}}\in \mathscr {C}_{m+1,n}(\varepsilon ,\vec {\textbf{y}})\\ \vec {\textbf{y}}\in \mathscr {C}_{m,m+1}(\varepsilon ,\vec {\textbf{x}}_m^{\textbf{e}}) \end{array}} \Psi ^{\varepsilon ,\delta }_{m+1,n}(\vec {\textbf{x}}) \alpha _n(\vec {\textbf{x}})\, \nearrow \sum _{\begin{array}{c} \vec {\textbf{x}}\in \mathscr {C}_{m+1,n}(\varepsilon ,\vec {\textbf{y}})\\ \vec {\textbf{y}}\in \mathscr {C}_{m,m+1}(\varepsilon ,\vec {\textbf{x}}_m^{\textbf{e}}) \end{array}} \alpha _n(\vec {\textbf{x}}) \,, $$

or equivalently, that

7.38 $$\begin{aligned} \sum _{\vec {\textbf{y}}\in \mathscr {C}_{m,m+1}(\varepsilon ,\vec {\textbf{x}}_m^{\textbf{e}})} \alpha _{m+1,n}^{\varepsilon ,\delta }(\vec {\textbf{y}}) \nearrow \sum _{\vec {\textbf{y}} \in \mathscr {C}_{m,m+1}(\varepsilon ,\vec {\textbf{x}}_m^{\textbf{e}})} \alpha _{m+1,n}^{\varepsilon }(\vec {\textbf{y}}) \,. \end{aligned}$$

#### Lemma 7.8

For any $0 \le m < n \le N$ and $\vec {E}, \vec {R}$, conditionally on $\mathrm{\textsf {CP}}(\vec {\textbf{x}}_m^{\textbf{e}},\vec {E},\vec {R})$, almost surely,

$$\begin{aligned} \Big \{ \vec {\textbf{x}}\in \mathbb {A}_{m,n}(\vec {\textbf{x}}_m^{\textbf{e}}):\, \forall m< i\le n,\, \bar{\nabla }H_{N}(\textbf{x}_i)=0,\, \min _{m < j\le n}\eta _j(\vec {\textbf{x}})= 0 \Big \} = \varnothing . \end{aligned}$$

#### Proof

Fixing $N \ge n > m \ge 0$ and $\vec {E},\vec {R}$, it suffices to show that conditionally on $\mathrm{\textsf {CP}}(\vec {\textbf{x}}_m^{\textbf{e}},\vec {E},\vec {R})$, a.s.,

$$ \Big \{ \vec {\textbf{x}}\in \mathbb {A}_{m,n}(\vec {\textbf{x}}_m^{\textbf{e}}):\, \forall m < i\le n,\, \bar{\nabla }H_{N}(\textbf{x}_i)=0,\, \eta _{n}(\vec {\textbf{x}})= 0 \Big \} = \varnothing . $$

We start with the unconditional version of m=0, whereby setting

$$ \mathscr {C}_n := \Big \{ \vec {\textbf{x}}\in \mathbb {A}_{n}:\, \forall i\le n,\, \bar{\nabla }H_{N}(\textbf{x}_i)=0 \Big \}, $$

we proceed to show that $\{\vec {\textbf{x}}\in \mathscr {C}_n:\,\eta _n(\vec {\textbf{x}})=0\}=\varnothing$ a.s.

Indeed, note that $\mathbb {A}_n\subset \mathbb {R}^{N\times n}$ is a compact manifold of dimension $d_0:= \sum _{i=1}^n (N-i)$ (which is diffeomorphic to the Stiefel manifold $V_n(\mathbb {R}^N)$ of orthonormal *n*-frames in $\mathbb {R}^N$) and that the Euclidean structure on $\mathbb {R}^{N\times n}$ induces a Riemannian metric on $\mathbb {A}_n$, whose distance function and volume form we denote by $d(\cdot ,\cdot )$ and *dv*. Let $\text{ Vol }(\mathbb {A}_n)$ and $\text{ Vol }(\varepsilon )$ denote the volume wrt *dv* of $\mathbb {A}_n$ and of a ball of radius ε in $\mathbb {A}_n$ around $\vec {\textbf{x}}\in \mathbb {A}_n$, which by symmetry does not depend on $\vec {\textbf{x}}$.

Note that the mappings $\vec {\textbf{x}}\mapsto \Vert \bar{\nabla }H_{N}(\textbf{x}_i)\Vert$, m<i≤n and $\vec {\textbf{x}}\mapsto \eta _n(\vec {\textbf{x}})$ are Lipschitz on $\mathbb {A}_n$ with some random Lipschitz constant $L_n=L_{N,n}$ which depends on the smooth function $H_N(\cdot )$ (but is independent of our choice of $M_i(\vec {\textbf{x}})$). Thus, denoting by $\mathcal {E}(c)$ the event that $L_n <c$ and $\{\vec {\textbf{x}}\in \mathscr {C}_n:\,\eta _n(\vec {\textbf{x}})=0\}\ne \varnothing$, to finish the proof, it suffices to show that $\mathbb {P}\{\mathcal {E}(c)\}=0$ for any fixed, finite *c*.

For any $\vec {\textbf{x}}'\in \mathbb {A}_n$, define the event

$$ \mathscr {D}_{\vec {\textbf{x}}'} := \{ \forall m< i\le n,\, \Vert \bar{\nabla }H_{N}(\textbf{x}'_i)\Vert<c\varepsilon ,\, \eta _n(\vec {\textbf{x}}')<c\varepsilon \} \,. $$

Note that by rotational invariance, $\mathbb {P}(\mathscr {D}_{\vec {\textbf{x}}'})$ is the same for any fixed, non-random $\vec {\textbf{x}}'\in \mathbb {A}_n$. In particular, $\mathbb {P}(\mathscr {D}_{\vec {\textbf{x}}'})=\mathbb {P}(\mathscr {D}_{\vec {\textbf{x}}^{\textbf{e}}_n})$ for such $\vec {\textbf{x}}'\in \mathbb {A}_n$. Now, if $\mathcal {E}(c)$ occurs then: first, there exists some $\vec {\textbf{x}}\in \mathbb {A}_n$ such that $\bar{\nabla }H_{N}(\textbf{x}_i)=0$ for m<i≤n and $\eta _n(\vec {\textbf{x}})=0$; second, for any $\vec {\textbf{x}}'\in \mathbb {A}_n$ within distance ε from $\vec {\textbf{x}}$ the event $\mathscr {D}_{\vec {\textbf{x}}'}$ holds . It follows that the expected total volume of the subset of $\vec {\textbf{x}}' \in \mathbb {A}_n$ for which $\mathscr {D}_{\vec {\textbf{x}}'}$ holds, is at least $\mathbb {P}\{\mathcal {E}(c)\} \text{ Vol }(\varepsilon )$. On the other hand, by Fubini’s theorem, that expected total volume is $\mathbb {P}(\mathscr {D}_{\vec {\textbf{x}}^{\textbf{e}}_n})\text{ Vol }(\mathbb {A}_n)$. Hence,

7.39 $$\begin{aligned} \mathbb {P}\{\mathcal {E}(c)\} \text{ Vol }(\varepsilon ) \le \mathbb {P}(\mathscr {D}_{\vec {\textbf{x}}^{\textbf{e}}_n})\text{ Vol }(\mathbb {A}_n) \,. \end{aligned}$$

Invoking rotational invariance once more, we note that $\mathbb {P}(\mathscr {D}_{\vec {\textbf{x}}^{\textbf{e}}_n})$ does not depend on the specific choice of $M_{i}=M_{i}(\vec {\textbf{x}}^{\textbf{e}}_n)$, as long as its rows form an orthonormal basis of $\big (\textrm{sp}\{\textbf{x}^{\textbf{e}}_1,\ldots ,\textbf{x}^{\textbf{e}}_{i}\}\big )^{\perp }$. Suppose that the *j*-th row of $M_i$ is the standard basis element $\textbf{e}_{j+i}\in \mathbb {R}^N$. Let m≤n-1 and take $\textbf{z}=(T^{(m)})^{-1}(\textbf{x}^{\textbf{e}}_{m+1})=\sqrt{N-m}\,\textbf{e}_1\in \mathbb {R}^{N-m}$, where we use the same notation as in Corollary 7.4 (and by abuse of notation, denote by $\textbf{e}_i$ the basis elements in both $\mathbb {R}^N$ and $\mathbb {R}^{N-m}$). By Corollary 7.4,

$$ \frac{\partial _{\textbf{x}^{\textbf{e}}_{m+1}-\textbf{x}^{\textbf{e}}_{m}}H_{N}(\textbf{x}^{\textbf{e}}_{m+1})}{\Vert \textbf{x}^{\textbf{e}}_{m+1}-\textbf{x}^{\textbf{e}}_{m}\Vert ^2} = \sqrt{\frac{N-m}{N}}\frac{1}{q_{m+1}-q_{m}} \frac{\partial _{z_1} H^{(m)}_{N}(\textbf{z})}{\Vert \textbf{z}\Vert }. $$

By working with the definitions, one can also check that

$$\begin{aligned} (\bar{\nabla }H_N(\textbf{x}^{\textbf{e}}_{m+1}))_{i}&= \frac{1}{\sqrt{q_{m+1}-q_{m}}} \partial _{z_{i+1}} H^{(m)}_{N}(\textbf{z}),\\ (\bar{\nabla }^{2}H_N(\textbf{x}^{\textbf{e}}_{m+1}))_{ij}&= \sqrt{\frac{N-m}{N}}\frac{1}{q_{m+1}-q_{m}} \partial _{z_{i+1}}\partial _{z_{j+1}} H^{(m)}_{N}(\textbf{z}) . \end{aligned}$$

Finally, in the notation of Corollary 7.5, for an appropriate choice of $F_i$, (see e.g. [13, Eq. (7.11)])

$$\begin{aligned} \nabla _{\textrm{sp}}H^{(m)}_{N}(\textbf{z})&= \big (\partial _{z_{i+1}} H^{(m)}_{N}(\textbf{z}) \big )_{i=1}^{N-m-1}, \\ \nabla _{\textrm{sp}}^{2}H^{(m)}_{N}(\textbf{z})&= \big (\partial _{z_{i+1}}\partial _{z_{j+1}} H^{(m)}_{N}(\textbf{z}) \big )_{i,j=1}^{N-m-1}-\frac{\partial _{z_1} H^{(m)}_{N}(\textbf{z})}{\Vert \textbf{z}\Vert }\textbf{I}. \end{aligned}$$

By Corollary 7.5, given $\mathrm{\textsf {CP}}(\vec {\textbf{x}}_m^{\textbf{e}},\vec {E},\vec {R})$ which includes $\bar{\nabla }H_N(\textbf{x}_i^{\textbf{e}})$, i≤m, the vector $\bar{\nabla }H_N(\textbf{x}_{m+1}^{\textbf{e}})$ has positive density on $\mathbb {R}^{N-(m+1)}$. Proceeding inductively over $1 \le m \le n$, we find that unconditionally, the Gaussian $X:=\big (\bar{\nabla }H_N(\textbf{x}_i^{\textbf{e}})\big )_{i\le n}$ has positive density on $\mathbb {R}^{d_0}$. From Corollary 7.5 we further have that conditionally on $\mathrm{\textsf {CP}}(\vec {\textbf{x}}_n^{\textbf{e}},\vec {E},\vec {R})$ which includes *X*, the variable $\eta _n(\vec {\textbf{x}}_n^{\textbf{e}})$ is the minimal eigenvalue (in absolute value), of a scaled (N-n-1)-dimensional goe matrix, shifted by an independent random multiple of the identity. As such, this variable clearly has a positive density. Thus, unconditionally, $(X,\eta _n(\vec {\textbf{x}}_n^{\textbf{e}}))$ has a positive joint density on $\mathbb {R}^{d_0+1}$ and consequently, the probability on the rhs of (7.39) is of order $O(\varepsilon ^{d_0+1})$. Of course, $\text{ Vol }(\varepsilon )=\Theta (\varepsilon ^{d_0})$, so upon dividing both sides by $\text{ Vol }(\varepsilon )$ and taking $\varepsilon \rightarrow 0$, we conclude that $\mathbb {P}\{\mathcal {E}(c)\}=0$.

For m≥1 we run a similar argument, for the conditional law $\mathbb {P}_0$ on the compact manifold $\mathbb {A}_{m,n}(\vec {\textbf{x}}_m^{\textbf{e}})$ of dimension $d_m:=\sum _{i>m}^n (N-i)$, given $\mathrm{\textsf {CP}}(\vec {\textbf{x}}^\textbf{e}_m,\vec {E},\vec {R})$, now considering

$$ \mathscr {C}_{m,n} := \big \{ \vec {\textbf{x}}\in \mathbb {A}_{m,n}(\vec {\textbf{x}}_m^{\textbf{e}}):\, \forall m< i\le n,\, \bar{\nabla }H_{N}(\textbf{x}_i)=0\big \} \,, $$

with $\mathcal {E}(c)$ the event that $L_n <c$ and $\{\vec {\textbf{x}}\in \mathscr {C}_{m,n}:\,\eta _n(\vec {\textbf{x}})=0\}\ne \varnothing$ (where now $L_n$ is measured wrt the distance on $\mathbb {A}_{m,n}(\vec {\textbf{x}}_m^{\textbf{e}})$). Having under $\mathbb {P}_0$ the relevant symmetries for $\mathscr {D}_{\vec {\textbf{x}}'}$ within $\mathbb {A}_{m,n}(\vec {\textbf{x}}_m^{\textbf{e}})$, we get similarly to the derivation of (7.39), that

$$\begin{aligned} \mathbb {P}_0\{\mathcal {E}(c)\} \text{ Vol }(\varepsilon ) \le \mathbb {P}_0(\mathscr {D}_{\vec {\textbf{x}}_n^{\textbf{e}}})\text{ Vol }(\mathbb {A}_{m,n}(\vec {\textbf{x}}_m^{\textbf{e}})) \,. \end{aligned}$$

As $\text{ Vol }(\varepsilon )=\Theta (\varepsilon ^{d_m})$, it thus suffices to show that for m<n, the collection $\big (\big (\bar{\nabla }H_N(\textbf{x}_i^{\textbf{e}})\big )_{m < i\le n},\eta _{n}(\vec {\textbf{x}}_{n}^{\textbf{e}})\big )$ has strictly positive density under $\mathbb {P}_0$. For the latter task we utilize as before Corollary 7.5 conditional on $\mathrm{\textsf {CP}}(\vec {\textbf{x}}_m^{\textbf{e}},\vec {E},\vec {R})$, now using the fact that $\mathrm{\textsf {CP}}(\vec {\textbf{x}}_{m+1}^{\textbf{e}},\vec {E},\vec {R}) \subset \mathrm{\textsf {CP}}(\vec {\textbf{x}}_{m}^{\textbf{e}},\vec {E},\vec {R})$ in order to remain throughout the proof under the conditional law $\mathbb {P}_0$. □

#### Lemma 7.9

For any $\delta \in (0,\varepsilon /2)$, the function $\vec {\textbf{y}}\mapsto \alpha ^{\varepsilon ,\delta }_{m,n}(\vec {\textbf{y}})$ is a.s. continuous on $\mathbb {A}_m$.

#### Proof

Recall that $H_N(\cdot )$ is a.s. continuously differentiable up to order three over the ball of radius $\sqrt{N}$, e.g. by [13, Corollary C.2]. Hence, it will be enough to prove the stated continuity for fixed deterministic *N* and $H_N(\cdot )$ with the same property, as we shall. Denote by $C_N'$ the maximal directional derivative up to order 3 over the ball, which is finite by compactness. Since rotations of $H_N(\cdot )$ preserve the above differentiability property, do not change the value of $C_N'$, and act as rotations also on the function $\vec {\textbf{y}}\mapsto \alpha ^{\varepsilon ,\delta }_{m,n}(\vec {\textbf{y}})$, wlog it is sufficient to prove continuity only at $\vec {\textbf{y}}=\vec {\textbf{y}}^\textbf{e}_m=(\textbf{y}_1^{\textbf{e}},\ldots ,\textbf{y}_m^{\textbf{e}}) \in \mathbb {A}_m$ where $\textbf{y}^{\textbf{e}}_i:= \sum _{j=1}^i \sqrt{N(q_j-q_{j-1})} \textbf{e}_j$.

Recall that $M_{i}=M_{i}(\vec {\textbf{x}})\in \mathbb {R}^{(N-i)\times N}$ is a matrix whose rows form an orthonormal basis of $\big (\textrm{sp}\{\textbf{x}_1,\ldots ,\textbf{x}_{i}\}\big )^{\perp }$ and define, for n>m,

$$\begin{aligned} \mathbb {A}_{m,n}^*:=\Big \{\vec {\textbf{x}}\in \mathbb {A}_{n}:\, M_{i}(\vec {\textbf{x}})\nabla H_N(\textbf{x}_{i}) = 0,\,\forall m+1\le i\le n\Big \}. \end{aligned}$$

The main step of the proof is to show using the implicit function theorem that for any m<n≤k

and $\vec {\textbf{x}}\in \mathbb {A}^*_{m,n}(\vec {\textbf{y}}):=\mathbb {A}_{m,n}(\vec {\textbf{y}})\cap \mathbb {A}^*_{m,n}$ (for $\mathbb {A}_{m,n}(\vec {\textbf{y}})$ of (7.17)), with $\eta _i(\vec {\textbf{x}})>\delta$ for $\eta _i(\vec {\textbf{x}})$ of (7.37) and all m<i≤n, there exists a function $f_{\vec {\textbf{y}},\vec {\textbf{x}}}$ from a small neighborhood of $\vec {\textbf{y}}$ in $\mathbb {A}_m$ (wrt the distance $\Vert \vec {\textbf{y}}-\vec {\textbf{y}}'\Vert ^2:=\sum _{i=1}^m \Vert \textbf{y}_i-\textbf{y}'_i\Vert ^2$) to $\mathbb {R}^{(n-m)\times N}$, such that:

7.40 $$\begin{aligned} \begin{aligned} \vec {\textbf{x}}':= (\vec {\textbf{y}}',f_{\vec {\textbf{y}},\vec {\textbf{x}}}(\vec {\textbf{y}}'))&\in \mathbb {A}^*_{m,n}(\vec {\textbf{y}}')\,, \\ \Vert f_{\vec {\textbf{y}},\vec {\textbf{x}}}(\vec {\textbf{y}}')-(\textbf{x}_{m+1},\ldots ,\textbf{x}_{n})\Vert&\le C \Vert \vec {\textbf{y}}'-\vec {\textbf{y}}\Vert , \end{aligned} \end{aligned}$$

for some *C* that depends only on δ, *N*, $(q_i)_{i=1}^{k}$ and $C_N'$.

Note that, in fact, by induction it suffices to establish (7.40) only for n=m+1, because we can compose such functions that increase the index by 1 to obtain the required function with general n>m (here it is important that the assumed bound $\eta _i(\vec {\textbf{x}})>\delta$ holds for all m<i≤n). Moreover, since rotations of $H_N(\cdot )$ also operate as rotations on $\eta _{m+1}(\vec {\textbf{x}})$, to prove the existence of such a function $f_{\vec {\textbf{y}},\vec {\textbf{x}}}$ for general $\vec {\textbf{x}}\in \mathbb {A}^*_{m,m+1}(\vec {\textbf{y}})$ it is sufficient to prove it under the assumption that $\vec {\textbf{x}}\in \mathbb {A}^*_{m,m+1}(\vec {\textbf{y}})$ and $\vec {\textbf{x}}=\vec {\textbf{x}}^\textbf{e}_{m+1}=(\textbf{x}_1^{\textbf{e}},\ldots ,\textbf{x}_{m+1}^{\textbf{e}})$ where $\textbf{x}^{\textbf{e}}_i:= \sum _{j=1}^i \sqrt{N(q_j-q_{j-1})} \textbf{e}_j$, as we shall. Note that in this case, we may also assume that the rows $M_i(\vec {\textbf{x}})$ are the standard basis vectors $\textbf{e}_{i+1},\ldots ,\textbf{e}_{N}$.

Further, fixing n=m+1, m≥1, we claim that (7.40) then follows from the implicit function theorem if, given $\vec {\textbf{y}}=\vec {\textbf{y}}_m^\textbf{e}$ and $\vec {\textbf{x}}=\vec {\textbf{x}}_n^\textbf{e}\in \mathbb {A}^*_{m,m+1}(\vec {\textbf{y}})$ as above, we have on some neighborhoods *A* and *B* of the origin in $\mathbb {R}^{N-1+\cdots +N-m}$ and $\mathbb {R}^{N-(m+1)}$, respectively, deterministic functions $\psi _m:A\rightarrow \mathbb {A}_m$ and $\Gamma _m:A\times B\rightarrow \mathbb {A}_{m+1}$, with $\Theta _m(\textbf{a},\textbf{b}):= H_N((\Gamma _m(\textbf{a},\textbf{b}))_{m+1})$ (where $(\Gamma _m(\textbf{a},\textbf{b}))_{i}$ is the *i*-th element of $\Gamma _m(\textbf{a},\textbf{b})$), such that:

1. $\psi _m$ is invertible on *A* and $\psi _m(0)=\vec {\textbf{y}}$.
2. $\Gamma _m(\textbf{a},\textbf{b}) \in \mathbb {A}_{m,m+1}(\psi _m(\textbf{a}))$ is twice continuously differentiable on A×B and $\Gamma _m(0,0)=\vec {\textbf{x}}$.
3. The functions $\vec {\textbf{y}}'\mapsto \psi _m^{-1}(\vec {\textbf{y}}')$ and $(\vec {\textbf{y}}',\textbf{b})\mapsto (\Gamma _m( \psi _m^{-1}(\vec {\textbf{y}}'),\textbf{b}))_{m+1}$ are Lipschitz on a small neighborhood of $(\vec {\textbf{y}},0)$ in $\mathbb {A}_m\times B$ and the function $\Gamma _m$ is Lipschitz on a small neighborhood of the origin with constants that depend only on *N* and $(q_i)_{i=1}^{k}$.
4. $$\Gamma _m(\textbf{a},\textbf{b})\in \mathbb {A}^*_{m,m+1}(\psi _m(\textbf{a}))\iff \frac{d}{db_i}\Theta _m(\textbf{a},\textbf{b})=0,\,\forall i\le N-(m+1)$$.
5. The Hessian matrix of $\Theta _m$ in $\textbf{b}$ at the origin, namely, $\Big (\frac{d}{db_i}\frac{d}{db_j}\Theta _m(0,0)\Big )_{i,j=1}^{N-(m+1)}$ is equal to
  7.41 $$\begin{aligned} M_{m+1}(\vec {\textbf{x}})\nabla ^2 H_N(\textbf{x}_{m+1}) M_{m+1}(\vec {\textbf{x}})^{\top } - \frac{\langle \nabla H_N(\textbf{x}_{m+1}), \textbf{x}_{m+1}-\textbf{x}_{m}\rangle }{\Vert \textbf{x}_{m+1}-\textbf{x}_m\Vert ^2}\textbf{I}. \end{aligned}$$

Indeed, by Point (2) the function $\big (\frac{d}{db_i}\Theta _m(\textbf{a},\textbf{b})\big )_{i\le N-(m+1)}$ is continuously differentiable on A×B. Moreover, since $\vec {\textbf{y}}\in \mathbb {A}_m$ and $\vec {\textbf{x}}\in \mathbb {A}_{m,m+1}^*(\vec {\textbf{y}})$ with $\eta _{m+1}(\vec {\textbf{x}})>\delta /2$, by Points (1), (2) and (4), $\big (\frac{d}{db_i}\Theta _m(0,0)\big )_{i\le N-(m+1)}=0$, whereas by point (5), the matrix $\big (\frac{d}{db_i}\frac{d}{db_j}\Theta _m(0,0)\big )_{i,j\le N-(m+1)}$ is invertible. Therefore, the implicit function theorem defines for any $\textbf{a}\in A$ in a small neighborhood of the origin, some $\textbf{b}(\textbf{a})\in B$ such that the condition in Point (4) holds at $(\textbf{a},\textbf{b}(\textbf{a}))$. For any $\vec {\textbf{y}}'$ close to $\vec {\textbf{y}}$ we can thus define $\textbf{a}'= \psi _m^{-1}(\vec {\textbf{y}}')$, $\textbf{b}'=\textbf{b}(\textbf{a}')$ and $\vec {\textbf{x}}' = \Gamma _m(\textbf{a}',\textbf{b}')\in \mathbb {A}_{m,m+1}^*(\vec {\textbf{y}}')$. Precisely, this defines

$$ f_{\vec {\textbf{y}},\vec {\textbf{x}}}(\vec {\textbf{y}}')= \Big (\Gamma _m\big (\psi _m^{-1}(\vec {\textbf{y}}'),\textbf{b}(\psi _m^{-1}(\vec {\textbf{y}}'))\big )\Big )_{m+1}\,. $$

By Point (5) and the assumption that $\eta _{m+1}(\vec {\textbf{x}})>\delta$, from the implicit function theorem, on a small neighborhood of 0∈A, the gradient of $\textbf{a}\mapsto \textbf{b}(\textbf{a})$ is bounded by some constant $C_0$ that depends on δ and $C_N'$. Combining this bound on the gradient of $\textbf{b}(\textbf{a})$ with Point (3) then yields the required bound as in (7.40).

We proceed to complete the proof of (7.40) by explicitly constructing the functions $\psi _m$ and $\Gamma _m$ as above. To this end, note first that we may parameterize $\mathbb {A}_m$ by $\chi _m:=\prod _{i=1}^m \mathbb {S}^{N-i}(1)$ where $\mathbb {S}^{N-i}(1):=\{\textbf{x}\in \mathbb {R}^{N-i+1}: \Vert \textbf{x}\Vert =1\}$. Precisely, map any $\vec {\textbf{z}}\in \chi _m$ to $\Phi _m(\vec {\textbf{z}})=(\Phi _{m,i}(\vec {\textbf{z}}))_{i=1}^m\in \mathbb {A}_m$ defined by^9^

7.42 $$\begin{aligned} \begin{aligned} \Phi _{m,i}(\vec {\textbf{z}})&= \sqrt{Nq_1}\textbf{z}_1 + \sqrt{N(q_2-q_1)}O_1\circ T(\textbf{z}_2)+\cdots \\&+\sqrt{N(q_i-q_{i-1})}O_{1}\circ T\circ O_{2}\circ T\cdots O_{i-1}\circ T(\textbf{z}_i) , \end{aligned} \end{aligned}$$

where, for any ℓ, set $T(x_1,\ldots ,x_\ell ):=(x_1,\ldots ,x_\ell ,0)$ and $O_i$ is the (N-i+1)×(N-i+1) rotation matrix that maps $\textbf{e}_{N-i+1}$ to $\textbf{z}_i$ and acts as the identity on any vector in the orthogonal space to $\textbf{e}_{N-i+1}$ and $\textbf{z}_i$. Having done this, let $A\subset \mathbb {R}^{N-1+\cdots +N-m}$ be some neighborhood of the origin and fixing some smooth chart $\bar{\psi }_m:A\rightarrow \chi _m$, define $\psi _m:A\rightarrow \mathbb {A}_m$ by $\psi _m=\Phi _m\circ \bar{\psi }_m$, choosing the chart $\bar{\psi }_m$ so that $\vec {\textbf{y}}=\vec {\textbf{y}}_m^\textbf{e}=\psi _m(0)$.

With $\vec {\textbf{y}}=\vec {\textbf{y}}_m^\textbf{e}\in \mathbb {A}_m$ and $\vec {\textbf{x}}=\vec {\textbf{x}}_{m+1}^\textbf{e}=(\textbf{x}^\textbf{e}_1,\ldots ,\textbf{x}^\textbf{e}_{m+1})\in \mathbb {A}^*_{m,m+1}(\vec {\textbf{y}})$, note that

7.43 $$\begin{aligned} \begin{aligned} S(\vec {\textbf{y}})&:=\big \{\textbf{x}: (\vec {\textbf{y}},\textbf{x})\in \mathbb {A}_{m,m+1}(\vec {\textbf{y}})\big \}\\&\, =\Big \{\textbf{x}^\textbf{e}_{m}+\textbf{y}:\,\frac{1}{N}\Vert \textbf{y}\Vert ^2=q_{m+1}-q_{m},\,\langle \textbf{y},\textbf{e}_i\rangle =0,\forall i\le m\Big \}\,, \end{aligned} \end{aligned}$$

is an (N-m-1)-dimensional sphere of radius $\sqrt{N(q_{m+1}-q_m)}=\Vert \textbf{x}^\textbf{e}_{m+1}-\textbf{x}^\textbf{e}_m\Vert$. For any $\vec {\textbf{y}}'\in \mathbb {A}_m$ we map $\vec {\textbf{x}}\in \mathbb {A}_{m,m+1}(\vec {\textbf{y}})$ to $\vec {\textbf{x}}'=\Phi _{m+1}(\vec {\textbf{z}}',\textbf{z}_{m+1})\in \mathbb {A}_{m,m+1}(\vec {\textbf{y}}')$ where $\vec {\textbf{z}}=(\textbf{z}_1,\ldots ,\textbf{z}_{m+1})=\Phi ^{-1}_{m+1}(\vec {\textbf{x}})$ and $\vec {\textbf{z}}'= \Phi ^{-1}_{m}(\vec {\textbf{y}}')$. This induces a mapping $\pi _{\vec {\textbf{y}}'}:S(\vec {\textbf{y}})\rightarrow S(\vec {\textbf{y}}')$, which is an isometry. Let $B\subset \mathbb {R}^{N-(m+1)}$ be some small neighborhood of the origin and define $\Gamma _m(\textbf{a},\textbf{b})= (\psi _m(\textbf{a}),\pi _{\psi _m(\textbf{a})}(\psi ^m(\textbf{b})))$, where

7.44 $$\begin{aligned} \psi ^m:\, B \ni \textbf{b}\mapsto M_{m+1}^{\top } (\vec {\textbf{x}}_{m+1}^\textbf{e})\textbf{b}+\alpha (\textbf{x}^\textbf{e}_{m+1}-\textbf{x}^\textbf{e}_{m}) + \textbf{x}^\textbf{e}_{m}\in S(\vec {\textbf{y}}), \end{aligned}$$

for the unique α>0 such that $N(q_{m+1}-q_{m})\alpha ^2+\Vert \textbf{b}\Vert ^2=N(q_{m+1}-q_{m})$. Finally, recall that

7.45 $$\begin{aligned} \Theta _m(\textbf{a},\textbf{b})=H_N((\Gamma _m(\textbf{a},\textbf{b}))_{m+1})= H_N(\pi _{\psi _m(\textbf{a})}(\psi ^m(\textbf{b})))\,. \end{aligned}$$

With these definitions all the properties we required above from the functions $\psi _m$, $\Gamma _m$ and $\Theta _m$ can be checked either directly from the definitions or by simple geometric considerations (with an appropriate choice of the chart $\bar{\psi }_m$). The functions $\vec {\textbf{y}}'\mapsto \psi _m^{-1}(\vec {\textbf{y}}')$ and $(\vec {\textbf{y}}',\textbf{b})\mapsto (\Gamma _m( \psi _m^{-1}(\vec {\textbf{y}}'),\textbf{b}))_{m+1}$ defined after (7.43), which map between manifolds and do not depend on $H_N(\cdot )$, are easily seen to satisfy the Lipschitz bounds of Point (3). Point (4) follows since the push-forward of the standard basis $(\frac{d}{db_i})_{i\le N-(m+1)}$ of the tangent space to $\textbf{b}\in B$ by $\pi _{\psi _m(\textbf{a})}\circ \psi ^m$ is a basis $(\textbf{v}_i)_{i\le N-(m+1)}$ of the tangent space to $\pi _{\psi _m(\textbf{a})}(\psi ^m(\textbf{b}))\in S(\psi _m(\textbf{a}))$.

Therefore, using (7.45), the condition on the rhs of Point (4) holds if and only if the derivative of $H_N(\cdot )$ at $(\Gamma _m(\textbf{a},\textbf{b}))_{m+1}$ in any direction in the tangent space of $(\Gamma _m(\textbf{a},\textbf{b}))_{m+1}$ in $S(\psi _m(\textbf{a}))$ is zero. For Point (5) we note that upon substituting $\textbf{a}=0$ we obtain $\Theta _m(0,\textbf{b})=H_N(\psi ^m(\textbf{b}))$ and the second term in (7.41) comes from the term involving $\alpha =\alpha (\textbf{b})$ in the definition of $\psi ^m$.

We proceed to show that for any $\vec {\textbf{y}}\in \mathbb {A}_m$, the number of points $\vec {\textbf{x}} \in \mathbb {A}^*_{m,m+1}(\vec {\textbf{y}})$ such that $\eta _{m+1}(\vec {\textbf{x}})> \delta$ is bounded from above by a random constant *c* that depends only on *N*, $(q_i)_{i=1}^{k}$, δ and $C_N'$. To this end, let $\vec {\textbf{x}}=(\vec {\textbf{y}},\textbf{x}_{m+1})$ be such a point. Defining $\psi ^m$ as in (7.44) (but now with general $\vec {\textbf{x}}$), we write

$$\begin{aligned}&H_N(\psi ^m(\textbf{b}))=H_N\Big (\textbf{x}_{m+1}+M_{m+1}^{\top } (\vec {\textbf{x}})\textbf{b}+(\textbf{x}_{m+1}-\textbf{x}_{m})\Big (-\frac{1}{2}\frac{\Vert \textbf{b}\Vert ^2}{{N(q_{m+1}-q_{m})}}\\&+O\Big (\frac{\Vert \textbf{b}\Vert ^4}{{N^2(q_{m+1}-q_{m})^2}}\Big )\Big ) \Big )\\&= H_N(\textbf{x}_{m+1})+\frac{1}{2}\Big \langle M_{m+1}(\vec {\textbf{x}})\nabla ^2 H_N(\textbf{x}_{m+1}) M_{m+1}(\vec {\textbf{x}})^{\top } \\&- \frac{\partial _{\textbf{x}_{m+1}-\textbf{x}_{m}}H_{N}(\textbf{x}_{m+1})}{\Vert \textbf{x}_{m+1}-\textbf{x}_{m}\Vert ^2} \textbf{I}, \textbf{b}\textbf{b}^\top \Big \rangle +O(\Vert \textbf{b}\Vert ^3), \end{aligned}$$

where the linear term in $\textbf{b}$ was dropped since $M_{m+1}(\vec {\textbf{x}})\nabla H_N(\textbf{x}_{m+1})=0$ and the constant of the $O(\Vert \textbf{b}\Vert ^3)$ term depends only on *N*, $(q_i)_{i=1}^{k}$ and $C_N'$. Recall (7.37) and note that all the eigenvalues of the matrix have absolute value at least δ. Thus, there is a ball $B_0\subset B$ centered around the origin $0\in \mathbb {R}^{N-(m+1)}$ such that:

- The center point $0\in B_0$ is a critical point of $H_N(\psi ^m(\textbf{b}))$.
- Any $\textbf{b}\in B_0\setminus \{0\}$ is not a critical point of $H_N(\psi ^m(\textbf{b}))$.
- The radius of $B_0$ only depends on *N*, $(q_i)_{i=1}^{k}$, δ and $C_N'$.

The image $\psi ^m(B_0)$ is an open set in $S(\vec {\textbf{y}})$. It contains a ball $B_0'\subset S(\vec {\textbf{y}})$ with $\textbf{x}_{m+1}\in B_0'$ such that:

- The point $\textbf{x}_{m+1}$ is a critical point of $H_N(\cdot )$ as a function on $S(\vec {\textbf{y}})$.
- Any $\textbf{x}\in B_0'\setminus \{\textbf{x}_{m+1}\}$ is not a critical point of $H_N(\cdot )$ (again, as a function on $S(\vec {\textbf{y}})$).
- The radius of $B_0'$ only depends on *N*, $(q_i)_{i=1}^{k}$, δ and $C_N'$.

To make the dependence on $\vec {\textbf{x}}$ explicit we write $B_0'(\vec {\textbf{x}})$ and define $B_0''(\vec {\textbf{x}})$ as the same ball with half the radius. Note that for any $\vec {\textbf{x}} \in \mathbb {A}^*_{m,m+1}(\vec {\textbf{y}})$ such that $\eta _{m+1}(\vec {\textbf{x}})> \delta$ we have associated a ball $B_0''(\vec {\textbf{x}})$. Those balls are disjoint, they all have the same radius and the number of such disjoint balls bounded by a random constant *c* that depends only on *N*, $(q_i)_{i=1}^{k}$, δ and $C_N'$. Hence, the same bound follows for the number of points $\vec {\textbf{x}}$, as we claimed above.

Note that by induction we also have that for any n>m, the number of points $\vec {\textbf{x}}\in \mathbb {A}^*_{m,n}(\vec {\textbf{y}})$ such that $\eta _{i}(\vec {\textbf{x}})> \delta$ for all i=m+1,…,n is at most $c^{n-m}$. Further, $\eta _i(\vec {\textbf{x}})$ of (7.37) is a.s. continuous, so with $\bar{h}_\delta (\cdot )$ continuous, $\Psi ^{\varepsilon ,\delta }_{m,n}(\vec {\textbf{x}})$ is also a.s. continuous. Recall that $\alpha _{n}(\vec {\textbf{x}})$ is assumed to be a.s. continuous, so by compactness $\Psi ^{\varepsilon ,\delta }_{m,n}(\vec {\textbf{x}})\alpha _n(\vec {\textbf{x}})$ is uniformly continuous. Fix ζ>0 and τ>0 small enough so $\Vert \Psi ^{\varepsilon ,\delta }_{m,n}(\vec {\textbf{x}})\alpha _n(\vec {\textbf{x}})-\Psi ^{\varepsilon ,\delta }_{m,n}(\vec {\textbf{x}}')\alpha _n(\vec {\textbf{x}}') \Vert <\zeta$ whenever $\Vert \vec {\textbf{x}}-\vec {\textbf{x}}'\Vert <\tau$. Only at most $c^{n-m}$ points $\vec {\textbf{x}} \in \mathbb {A}^*_{m,n}(\vec {\textbf{y}})$ with $\min _{i>m}^n \{\eta _i(\vec {\textbf{x}})\}>\delta$ can contribute to $\alpha ^{\varepsilon ,\delta }_{m,n}(\vec {\textbf{y}})$, and if $\Vert \vec {\textbf{y}}'-\vec {\textbf{y}}\Vert <\tau /C$, then by utilizing $f_{\vec {\textbf{y}},\vec {\textbf{x}}}$ of (7.40), there exists some $\vec {\textbf{x}}' \in \mathbb {A}^*_{m,n}(\vec {\textbf{y}}')$ such that $\Vert \vec {\textbf{x}}-\vec {\textbf{x}}'\Vert < \tau$. Consequently, for any $\vec {\textbf{y}},\vec {\textbf{y}}'\in \mathbb {A}_m$ such that $\Vert \vec {\textbf{y}}'-\vec {\textbf{y}}\Vert <\tau /C$,

$$ \alpha ^{\varepsilon ,\delta }_{m,n}(\vec {\textbf{y}}') \ge \alpha ^{\varepsilon ,\delta }_{m,n}(\vec {\textbf{y}})-\zeta c^{n-m}. $$

Since the same inequality holds if we interchange $\vec {\textbf{y}}$ and $\vec {\textbf{y}}'$, the lemma follows. □

#### Proof of Proposition 7.1

Equipped with Lemmas 7.8 and 7.9, we fix $0 \le m \le n-1$ and proceed to derive (7.36). To this end, recall first that by definition (see (7.35)),

$$ \alpha _{m,n}^{\varepsilon }(\vec {\textbf{x}}_m^{\textbf{e}})\wedge e^{MN} \le \sum _{\vec {\textbf{y}} \in \mathscr {C}_{m,m+1}(\varepsilon ,\vec {\textbf{x}}_m^{\textbf{e}})}\big ( \alpha _{m+1,n}^{\varepsilon }(\vec {\textbf{y}})\wedge e^{MN}\big ). $$

Denoting hereafter by $\mathbb {E}_0$ the expectation conditional on $\mathrm{\textsf {CP}}(\vec {\textbf{x}}_{m}^\textbf{e},\vec {E},\vec {R})$, we thus have that

$$\begin{aligned} \mathbb {E}_0\Big [ \alpha _{m,n}^{\varepsilon }(\vec {\textbf{x}}_m^{\textbf{e}}) \wedge e^{MN} \,\Big ] \le \mathbb {E}_0 \Big [ \sum _{\vec {\textbf{y}}\in \mathscr {C}_{m,m+1}(\varepsilon ,\vec {\textbf{x}}_m^{\textbf{e}})}\big ( \alpha _{m+1,n}^{\varepsilon }(\vec {\textbf{y}})\wedge e^{MN}\big ) \Big ]. \end{aligned}$$

Hence, by our consequence (7.38) of Lemma 7.8 and the monotone convergence theorem,

7.46 $$\begin{aligned} \mathbb {E}_0\Big [ \alpha _{m,n}^{\varepsilon }(\vec {\textbf{x}}_m^{\textbf{e}}) \wedge e^{MN} \,\Big ] \le \lim _{\delta \rightarrow 0}\mathbb {E}_0\Big [ \sum _{\vec {\textbf{y}}\in \mathscr {C}_{m,m+1}(\varepsilon ,\vec {\textbf{x}}_m^{\textbf{e}})}\big ( \alpha _{m+1,n}^{\varepsilon ,\delta }(\vec {\textbf{y}})\wedge e^{MN}\big ) \Big ]. \end{aligned}$$

Lemma 7.9 about the a.s. continuity of $\vec {\textbf{y}} \mapsto \alpha _{m+1,n}^{\varepsilon ,\delta }(\vec {\textbf{y}})$ is basically deterministic. Precisely, its proof only uses that $\boldsymbol{\sigma }\mapsto H_N(\boldsymbol{\sigma })$ is twice continuously differentable. It therefore also holds conditionally on $\mathrm{\textsf {CP}}(\vec {\textbf{x}}_{m}^\textbf{e},\vec {E},\vec {R})$. Of course, $\Psi ^{\varepsilon ,\delta }_{m+1,n}(\vec {\textbf{x}})=\bar{\Psi }^{\varepsilon ,\delta }_{m+1,n}(\vec {\textbf{x}},H_N(\cdot ))$ for some rotationally invariant deterministic $\bar{\Psi }^{\varepsilon ,\delta }_{m+1,n}$ and one can therefore easily verify that $\alpha _{m+1,n}^{\varepsilon ,\delta }(\vec {\textbf{x}}) =\bar{\alpha }_{m+1,n}^{\varepsilon ,\delta }(\vec {\textbf{x}},H_N(\cdot ))$ for rotationally invariant $\bar{\alpha }_{m+1,n}^{\varepsilon ,\delta }$. Since taking maximum with $e^{MN}$ preserves both continuity and rotational invariance, subject to (7.29), we have from (7.34),^10^ that

7.47 $$\begin{aligned} \begin{aligned}&\mathbb {E}_0\Big [ \sum _{\vec {\textbf{y}} \in \mathscr {C}_{m,m+1}(\varepsilon ,\vec {\textbf{x}}_m^\textbf{e})} \!\! \big (\alpha _{m+1,n}^{\varepsilon ,\delta }(\vec {\textbf{y}})\wedge e^{MN}\big )\, \Big ] \le C_{N,m} \bigg \{ 6 e^{M N} e^{-C_N N} \\&+ \int _{I_1\times I_2} \Gamma _{N,R}(t_N)\Big ( e^{-N^2} + 5 \mathbb {E}\big [ \alpha _{m+1,n}^{\varepsilon ,\delta }(\vec {\textbf{x}}_{m+1}^{\textbf{e}})\wedge e^{MN} \, \big | \, \mathrm{\textsf {CP}}(\vec {\textbf{x}}_{m+1}^\textbf{e},\vec {E},\vec {R}) \big ] \Big )\\&\quad \varphi _{N,m}(E,R)dEdR \bigg \}\,, \end{aligned} \end{aligned}$$

where $C_N\rightarrow \infty$ and $t_N \rightarrow 0$ do not depend on δ or $(\vec {E},\vec {R},E,R)$ (see remark after (7.34)). Since $\alpha _{m+1,n}^{\varepsilon ,\delta }(\cdot )\le \alpha _{m+1,n}^{\varepsilon }(\cdot )$, we can and shall replace $\alpha _{m+1,n}^{\varepsilon ,\delta }(\vec {\textbf{y}})$ by $\alpha _{m+1,n}^{\varepsilon }(\vec {\textbf{y}})$ on the rhs of (7.47). At this point we take $\delta \downarrow 0$, and combining (7.46) with (7.47), arrive at

$$\begin{aligned} \begin{aligned} \mathbb {E}\big [&\alpha _{m,n}^{\varepsilon }(\vec {\textbf{x}}_m^{\textbf{e}} ) \wedge e^{MN} \, \big | \, \mathrm{\textsf {CP}}(\vec {\textbf{x}}_{m}^\textbf{e},\vec {E},\vec {R}) \big ] \le C_{N,m} \bigg \{ 6 e^{M N} e^{-C_N N} \\&+ \int _{I_1\times I_2} \Gamma _{N,R}(t_N) \Big ( e^{-N^2} + 5 \mathbb {E}\big [ \alpha _{m+1,n}^{\varepsilon }(\vec {\textbf{x}}^{\textbf{e}}_{m+1})\wedge e^{MN} \, \big | \, \mathrm{\textsf {CP}}(\vec {\textbf{x}}_{m+1}^\textbf{e},\vec {E},\vec {R}) \big ] \Big )\\&\quad \varphi _{N,m}(E,R)dEdR \bigg \}\,, \end{aligned} \end{aligned}$$

from which we get (7.36) by the same reasoning as we have done after (7.30). □

## Proof of Property (5) of Proposition 3.1

Recall from Section 3 that fixing some $c_N \rightarrow 0$ slowly enough, the vertex sets $\mathbb {V}_N \subset \mathbb {R}^N$ of full regular trees $\mathbb {T}_N$ of degrees $d_N \rightarrow \infty$ and depth *k* can with probability going to 1 as $N \rightarrow \infty$, be chosen so that for some non-random $\delta _N,\varepsilon _N \rightarrow 0$, the collections $\mathbb {V}_N$ and the disjoint subsets $\{B_i, i \le d_N^k\}$ of $\mathbb {S}^{N-1}$ associated with the leaves of $\mathbb {T}_N$, have all the properties stated in Proposition 3.1, except possibly Property (5). Hereafter, we call such $(\mathbb {T}_N,(B_i)_i,c_N,d_N,\delta _{N},\varepsilon _{N})$ a *good pure state decomposition*. For any m≤k, denote by $\mathbb {V}_{N, m}$ the subset of $\mathbb {V}_N$ of depth *m*. In addition, for the root $\mathbb {V}_{N,0}$ we let $\vec {\textbf{v}}=\textbf{v}=\textbf{0}$, the origin in $\mathbb {R}^N$, while for any $\textbf{v}\in \mathbb {V}_{N,m}$ (with $|\textbf{v}|=m\ge 1$), we denote by $\vec {\textbf{v}}=(\textbf{v}_1,\ldots ,\textbf{v}_m)$ the path from the root to $\textbf{v}=\textbf{v}_m$, excluding the root. Recalling the definition (6.24) of $\mathscr {C}^0_m(\varepsilon )$, it suffices to show by induction on m≤k, that

8.1 $$\begin{aligned} \begin{aligned}&\text{ there } \text{ exists } \text{ a } \text{ good } \text{ pure } \text{ states } \text{ decomposition } \text{ with } \vec {\textbf{v}} \in \mathscr {C}^0_m(\varepsilon _N)\text { for all }\textbf{v}\in \mathbb {V}_{N,m}. \end{aligned} \end{aligned}$$

Indeed, from Corollary 6.7 we know that for any fixed $\varepsilon^{\prime }>0$, for some $$\eta _N=\eta _N(\varepsilon ')\rightarrow 0$$,

$$ \mathbb {P}( \mathscr {C}^0_{k}(\varepsilon _{N}) \not \subset \mathscr {C}_{k}(\varepsilon ') ) \le \eta _N\,. $$

Hence, after further enlarging $\varepsilon _N$, Proposition 3.1 follows from (8.1) at m=k.

In case m=0, we obviously have (8.1). Thus, we assume hereafter that (8.1) holds for some m≥0 and show that it then must hold also at m+1, by finding non-random $a_N, b_N \rightarrow 0$ (with $a_N \ge \varepsilon _N$), and a (random) mapping $\textbf{v}_{m+1} \mapsto \textbf{v}'_{m+1}$ of $\mathbb {V}_{N,m+1}$, such that with probability going to 1 as $N \rightarrow \infty$,

8.2 $$\begin{aligned} \sup _{\mathbb {V}_{N,m+1}}&\Vert \textbf{v}'_{m+1} - \textbf{v}_{m+1} \Vert \le b_N \sqrt{N} \quad \text {and} \quad \{ \vec {\textbf{v}}'_{m+1} \} \subset \mathscr {C}^0_{m+1}(a_N) \,. \end{aligned}$$

Indeed, keeping the disjoint sets $\{B_i\}$ of our good pure state decomposition, while increasing both $\delta _N$ and $\varepsilon _N$ by $4 b_N$, induces Properties (1)-(4) of Proposition 3.1 for our modified vertex set. Likewise, (3.4) transfers to the modified vertex set, since whp $\textbf{v}\mapsto H_N(\textbf{v})$ is $C\sqrt{N}$-Lipschitz and enlarging $\varepsilon _N$ to $a_N$, we now have that (8.1) holds at m+1.

Turning to establish (8.2), recall that $\mathscr {C}_m^0(\varepsilon ) \subset \mathbb {A}_m$ of (3.1) and the definition (7.43) of $S(\vec {\textbf{y}})$. Setting $r_{m+1}:= \sqrt{N (q_{m+1}-q_m)}$, for each vertex $\textbf{v}_{m}\in \mathbb {V}_{N,m}$ at level *m* we denote by p(·) the orthogonal projection onto $\text{ span }\{\textbf{v}_1,\ldots ,\textbf{v}_{m}\}^{\perp }$ and consider the decomposition obtained by replacing each of its descendants $\textbf{v}_{m+1}\in \mathbb {V}_{N,m+1}$ by its image $\textbf{u}_{m+1}$ under the mapping

$$\begin{aligned} \textbf{v}_{m+1} \mapsto \textbf{u}_{m+1} := \textbf{v}_{m}+\frac{r_{m+1}}{\Vert p(\textbf{x})\Vert } p(\textbf{x}) \in S(\vec {\textbf{v}}_m) \,, \quad \text {where} \quad \textbf{x}:=\textbf{v}_{m+1}-\textbf{v}_m \,. \end{aligned}$$

Now, with $\{ \textbf{v}_i-\textbf{v}_{i-1}, i \le m\}$, orthogonal of norms $r_i$, we have for some finite $c_m=c_m(\{q_i\})$,

$$ \frac{1}{\sqrt{N}} \Vert \textbf{x}- p(\textbf{x})\Vert = \Big [ \sum _{i=1}^m \frac{1}{N r_i^2} \langle \textbf{x}, \textbf{v}_i-\textbf{v}_{i-1} \rangle ^2 \Big ]^{1/2} \le \frac{c_m}{N} \max _{i=1}^m |\langle \textbf{x},\textbf{v}_i\rangle | \le 2c_m \varepsilon _N \,, $$

since by Property (3) of Proposition 3.1, $|\langle \textbf{x},\textbf{v}_i \rangle | = |\langle \textbf{v}_{m+1},\textbf{v}_i\rangle - \langle \textbf{v}_{m},\textbf{v}_i\rangle | \le 2\varepsilon _N N$ for all i≤m (where for i=1, we define above $\textbf{v}_{i-1}=\textbf{v}_{0}=0$). Similarly, by our induction hypothesis, (8.1) holds. Hence, $\Vert \textbf{v}_m\Vert ^2 = N q_m$ with

$$ \big | \Vert \textbf{x}\Vert ^2 - r_{m+1}^2 \big | \le \big | \Vert \textbf{v}_{m+1}\Vert ^2 - N q_{m+1} \big | + 2 |\langle \textbf{v}_{m+1}, \textbf{v}_m \rangle - N q_m | \le 3 \varepsilon _N N $$

and since by the triangle inequality

$$\begin{aligned} \Vert \textbf{v}_{m+1}-\textbf{u}_{m+1}\Vert&\le \Vert \textbf{x}-p(\textbf{x})\Vert +\Vert p(\textbf{x})-r_{m+1} \frac{p(\textbf{x})}{\Vert p(\textbf{x})\Vert } \Vert \\&\!=\! \Vert \textbf{x}\!-\!p(\textbf{x})\Vert \!+\!\big |\Vert p(\textbf{x})\Vert \!-\!r_{m+1} \big | \!\le \! 2 \Vert \textbf{x}\!-\!p(\textbf{x})\Vert \!+\! \big |\Vert \textbf{x}\Vert \!-\!r_{m+1}\big | \,, \end{aligned}$$

we deduce that $\Vert \textbf{v}_{m+1}-\textbf{u}_{m+1}\Vert \le b'_N \sqrt{N}$ for some $b'_N=b'_N(m,\varepsilon _N) \rightarrow 0$. As explained above, whp $H_N(\cdot )$ is $C\sqrt{N}$-Lipschitz and $\big |\frac{1}{N}H_N(\textbf{v}_{m+1})-E_{\star }(q_{m+1})\big | \le a_N$ for some non-random $a_N \rightarrow 0$. Hence, after suitably increasing $a_N$, we have that whp

8.3 $$\begin{aligned} \sup _{\mathbb {V}_{N,m+1}} \Big |\frac{1}{N}H_N(\textbf{u}_{m+1})-E_{\star }(q_{m+1})\Big | \le a_N. \end{aligned}$$

Now, having $\vec {\textbf{v}}_m \in \mathscr {C}_m^0(\varepsilon _N)$ and $\textbf{u}_{m+1} \in S(\vec {\textbf{v}}_m)$ satisfying (8.3), we apply our next lemma at $\varepsilon = a_N$ (and some $\tau =\tau _N \rightarrow 0$), to get with probability going to 1 as $N \rightarrow \infty$, a perturbation $\textbf{v}'_{m+1} \in S(\vec {\textbf{v}}_m)$ of $\textbf{u}_{m+1}$ for which (8.2) holds (say, with $b_N=b'_N+\tau _N$).

### Lemma 8.1

For any m≤k-1 and τ>0, if ε>0 is sufficiently small,

$$\begin{aligned}&\varlimsup _{N\rightarrow \infty } \frac{1}{N}\log \bigg (1- \mathbb {P}\Big [ \forall \vec {\textbf{x}}_m\in \mathscr {C}^0_m(\varepsilon ),\,\textbf{x}\in S(\vec {\textbf{x}}_m):\,\frac{1}{N}H_N(\textbf{x})\ge E_{\star }(q_{m+1})-\varepsilon \implies \\&\exists \textbf{x}'\in S(\vec {\textbf{x}}_m),\,\Vert \textbf{x}-\textbf{x}'\Vert \le \tau \sqrt{N}, (\vec {\textbf{x}}_m,\textbf{x}')\in \mathscr {C}^0_{m+1}(\varepsilon ) \Big ] \bigg )<0. \end{aligned}$$

### Proof

By the Borell-TIS inequality, with probability at least $1-e^{-cN}$ for some $c=c(\xi ,\varepsilon )$,

8.4 $$\begin{aligned} \sup _{\textbf{x}\in \sqrt{q_{m+1}}\mathbb {S}^{N-1}} \Big \{ \frac{1}{N} H_N(\textbf{x}) \Big \} \le E_{\star }(q_{m+1})+\varepsilon . \end{aligned}$$

Suppose $\vec {\textbf{x}}_m=(\textbf{x}_1,\ldots ,\textbf{x}_m)\in \mathscr {C}^0_m(\varepsilon )$ and recall that $S(\vec {\textbf{x}}_m)$ is a sphere of dimension N-m-1 and radius $\sqrt{N(q_{m+1}-q_m)}$. Consider the subsets

$$\begin{aligned} S^+_\varepsilon (\vec {\textbf{x}}_m)&:=\Big \{ \textbf{x}\in S(\vec {\textbf{x}}_m):\,\frac{1}{N}H_N(\textbf{x})\ge E_{\star }(q_{m+1})-\varepsilon \Big \},\\ S_\varepsilon (\vec {\textbf{x}}_m)&:=\Big \{ \textbf{x}\in S(\vec {\textbf{x}}_m):\,\Big |\frac{1}{N}H_N(\textbf{x})-E_{\star }(q_{m+1})\Big |\le \varepsilon \Big \}. \end{aligned}$$

Assume that the event in (8.4) occurs, so that $S_\varepsilon (\vec {\textbf{x}}_m)=S^+_\varepsilon (\vec {\textbf{x}}_m)$, since $S(\vec {\textbf{x}}_m)\subset \sqrt{q_{m+1}}\mathbb {S}^{N-1}$.

Each of the connected components of $S_\varepsilon (\vec {\textbf{x}}_m)=S^+_\varepsilon (\vec {\textbf{x}}_m)$ contains at least one local maximum and, in particular, a critical point $\textbf{x}_{m+1}$ of $H_N(\textbf{x})$. Namely, a point $\vec {\textbf{x}}_{m+1}=(\textbf{x}_1,\ldots ,\textbf{x}_{m+1})$ such that $\bar{\nabla }H_N(\textbf{x}_{m+1})=0$, where the latter gradient is defined above (3.3). To prove the implication as in the lemma, assuming (8.4), it is therefore sufficient to show that for any critical point $\textbf{x}_{m+1}$ in $S_\varepsilon (\vec {\textbf{x}}_m)$,

the connected component of $S_\varepsilon (\vec {\textbf{x}}_m)$ containing $\textbf{x}_{m+1}$ has diameter less than $\sqrt{N}\delta$ for some $\delta =\delta (\varepsilon ) \rightarrow 0$ as $\varepsilon \rightarrow 0$. The latter claim about the diameter follows if for some $\eta =\eta (\varepsilon ) \rightarrow 0$ and any such

$$\textbf{x}_{m+1}$$

8.5 $$\begin{aligned} \Upsilon _{N}(\vec {\textbf{x}}_{m+1}):=\frac{1}{N}\sup _{\begin{array}{c} \textbf{x}\in S(\vec {\textbf{x}}_m):\\ \frac{1}{N}\langle \textbf{x},\textbf{x}_{m+1}\rangle =q_{m+1}-\eta \end{array}} \Big \{ H_N(\textbf{x}) \Big \} < E_{\star }(q_{m+1})-\varepsilon \,, \end{aligned}$$

since this implies that any $\textbf{x}\in S(\vec {\textbf{x}}_m)$ with $\frac{1}{N}\langle \textbf{x},\textbf{x}_{m+1}\rangle =q_{m+1}-\eta$, equivalently with $\frac{1}{N}\Vert \textbf{x}-\textbf{x}_{m+1}\Vert ^2=2\eta$, does not belong to $S^+_\varepsilon (\vec {\textbf{x}}_m)$. We conclude that in order to prove the lemma, it suffices to show that for any fixed η>0 and $\varepsilon <\varepsilon _0(\eta )$,

8.6 $$\begin{aligned} \varlimsup _{N\rightarrow \infty } \frac{1}{N}\log \mathbb {E}\Big [1 \wedge \sum _{\vec {\textbf{x}} \in \mathscr {C}^0_{m+1}(\varepsilon )} 1\!\!1\big \{ \Upsilon _{N}(\vec {\textbf{x}})\ge E_{\star }(q_{m+1})- \varepsilon \big \} \Big ]&<0. \end{aligned}$$

By Corollary 6.7, with overwhelming probability, $\mathscr {C}^0_{m+1}(\varepsilon )\subset \mathscr {C}_{m+1}(\varepsilon ')$ whenever $\varepsilon <\varepsilon _1(\varepsilon ')$. Hence, (8.6) holds if for any fixed η, small enough $\varepsilon '=\varepsilon '(\eta )$ and any $\varepsilon <\varepsilon _1(\varepsilon ')$,

8.7 $$\begin{aligned} \varlimsup _{N\rightarrow \infty } \frac{1}{N}\log \mathbb {E}\Big [1 \wedge \sum _{\vec {\textbf{x}} \in \mathscr {C}_{m+1}(\varepsilon ')} 1\!\!1\big \{ \Upsilon _{N}(\vec {\textbf{x}}) \ge E_{\star }(q_{m+1})- \varepsilon \big \} \Big ]&<0. \end{aligned}$$

The bounded, continuous function $\tilde{h}_\varepsilon (t):= 1 \wedge (\frac{t}{\varepsilon } + 1)_+$ exceeds the indicator on $\mathbb {R}_+$, hence the lhs of (8.7) is bounded from above by

8.8 $$\begin{aligned} \varlimsup _{N\rightarrow \infty } \frac{1}{N}\log \mathbb {E}\Big [\Big (\sum _{\vec {\textbf{x}}\in \mathscr {C}_{m+1}(\varepsilon ') } \alpha _{m+1,\varepsilon }(\vec {\textbf{x}})\Big )\wedge 1 \Big ], \end{aligned}$$

where we have set

$$ \alpha _{m+1,\varepsilon }(\vec {\textbf{x}}) := \tilde{h}_\varepsilon (\Upsilon _{N}(\vec {\textbf{x}}) - E_{\star }(q_{m+1}) + \varepsilon )\,. $$

The [0, 1]-valued $\alpha _{m+1,\varepsilon }(\vec {\textbf{x}})=\bar{\alpha }_{N,m+1} (\vec {\textbf{x}},H_N(\cdot ))$ is a.s. continuous on $\mathbb {A}_{m+1}$, where the non-random, rotationally invariant $\bar{\alpha }_{N,m+1}(\vec {\textbf{x}},\varphi )$ that corresponds to replacing $H_N(\textbf{x})$ by $\varphi (\textbf{x})$ in (8.5), satisfies (7.2). Consequently, by Proposition 7.1, the limit in (8.8) is bounded from above by

8.9 $$\begin{aligned}&\sum _{n=0}^{m}\sup _{I_n(\varepsilon ')\times I'_n(\varepsilon ')}\Theta _{\xi _{(n)}}(E,R) +\varlimsup _{N\rightarrow \infty }\frac{1}{N}\log \nonumber \\&\quad \times \Big (\sup _{\bar{V}(\varepsilon ')}\mathbb {E}\big [ \alpha _{m+1,\varepsilon }(\vec {\textbf{x}}_{m+1}^{\textbf{e}}) \,\big |\,\mathrm{\textsf {CP}}(\vec {\textbf{x}}_{m+1}^\textbf{e},\vec {E},\vec {R}) \big ]\Big ), \end{aligned}$$

and we recall that

$$\begin{aligned} \begin{aligned} I_m(\varepsilon ')&=[E_{\star }(q_{m+1})-E_{\star }(q_m)-2\varepsilon ',E_{\star }(q_{m+1})-E_{\star }(q_m)+2\varepsilon '],\\ I'_m(\varepsilon ')&=(q_{m+1}-q_m)[R_{\star }(q_{m+1})-\varepsilon ',R_{\star }(q_{m+1})+\varepsilon ']. \end{aligned} \end{aligned}$$

Since $$(E,R)\mapsto \Theta _{\xi }(E,R)$$ is continuous, by Corollary 6.9, the sum over *n* in (8.9) can be made as small as we wish by taking $\varepsilon^{\prime }$ small enough.

By our choice of $\tilde{h}_\varepsilon$, the expectation in (8.9) is bounded from above by the conditional probability that $\Upsilon _{N}(\vec {\textbf{x}}_{m+1}^{\textbf{e}})\ge E_{\star }(q_{m+1})-2\varepsilon$. By Corollary 7.4, the latter conditional probability is equal to

$$\begin{aligned} \mathbb {P}\Big ( \frac{1}{N-m} \sup _{\begin{array}{c} \textbf{z}\in \mathbb {S}^{N-m-1}:\\ \frac{1}{N-m}\langle \textbf{z},\boldsymbol{\sigma }\rangle =1-\eta ' \end{array}} H_N^{(m)}(\textbf{z})\ge E'\,\Big |\,\boldsymbol{\sigma }\in \textrm{Crt}_{N-m,\xi _{(m)}}(E,R) \Big )\,, \end{aligned}$$

where $\boldsymbol{\sigma }\in \mathbb {S}^{N-m-1}$ is arbitrary, $\eta '=\frac{\eta }{q_{m+1}-q_m}$,

$$\begin{aligned} E'&= \sqrt{\frac{N}{N-m}}\Big ( E_{\star }(q_{m+1})-E_m-2\varepsilon \Big ),\\ E&= \sqrt{\frac{N}{N-m}}\big ( E_{m+1}-E_m\big ),\\ R&=\sqrt{\frac{N}{N-m}}\big (q_{m+1}-q_m\big ) R_{m+1}, \end{aligned}$$

and $\textrm{Crt}_{N-m,\xi _{(m)}}$ is defined as in Section 6, albeit now for the spherical model $H_N^{(m)}(\textbf{z})$ of mixture $\xi _{(m)}$.

For *N* sufficiently large, by choosing $\varepsilon^{\prime }$ sufficiently small we can make sure that uniformly over $$\bar{V}(\varepsilon ')$$, $E^{\prime }$, *E* and *R* are as close as we wish to $$E_{\star }(q_{m+1})-E_{\star }(q_m)-2\varepsilon $$, $$E_{\star }(q_{m+1})-E_{\star }(q_m)$$ and $$(q_{m+1}-q_m)R_{\star }(q_{m+1})$$, respectively. Hence, the proof is completed by Lemma 6.8 and Proposition 6.4, noting that the bound in Proposition 6.4 only improves as we decrease *a* and *b*. □

## Low temperature: proof of Theorem 1.4

Recall the measure

$$\begin{aligned} \Pi ^\varepsilon _{N,\beta }(\,\cdot \,):= \sum _{\vec {\textbf{x}}\in \mathscr {C}_{k}(\varepsilon )} G_{N,\beta }\big (\,\cdot \,\big |\, B(\vec {\textbf{x}},\varepsilon )\big ) =\sum _{\vec {\textbf{x}}\in \mathscr {C}_{k}(\varepsilon )} \frac{G_{N,\beta }\big (\,\cdot \,\cap \, B(\vec {\textbf{x}},\varepsilon )\big )}{G_{N,\beta }\big (B(\vec {\textbf{x}},\varepsilon )\big )}, \end{aligned}$$

and subset

$$\begin{aligned} B(\vec {\textbf{x}},\varepsilon )&:=\left\{ \boldsymbol{\sigma }\in \mathbb {S}^{N-1}:\,|\langle \boldsymbol{\sigma },\textbf{x}_i\rangle -\langle \textbf{x}_i,\textbf{x}_i\rangle |\le N\varepsilon ,\,\forall i\le k \right\} . \end{aligned}$$

which we defined in (5.1) and (1.24). We denote by $\mathcal {B}(\mathbb {S}^{N-1})$ the set of Borel measurable subsets of $\mathbb {S}^{N-1}$. From the discussion of Section 5.1, for $\varepsilon _N \rightarrow 0$ of Proposition 3.1 and any fixed ε>0,

$$\begin{aligned} \lim _{N\rightarrow \infty } \mathbb {P}\Big (\forall M\in \mathcal {B}(\mathbb {S}^{N-1}):\, \Pi _{N,\beta }^\varepsilon (M)\wedge 1\ge G_{N,\beta }(M)-\varepsilon _{N}\Big )=1 \end{aligned}$$

and thus to prove (1.27), it is enough to show that

9.1 $$\begin{aligned} \varlimsup _{\varepsilon \rightarrow 0}\varlimsup _{N\rightarrow \infty }\frac{1}{N}\log \Big (\mathbb {E}\Big [\Pi ^{\varepsilon }_{N,\beta }\big (f_{N}(\boldsymbol{\sigma })\in A \big )\wedge 1\Big ]\Big )<0. \end{aligned}$$

In contrast to Section 5.1, here we take the minimum with 1 which is justified since $G_{N,\beta }(\mathbb {S}^{N-1})= 1$.

For a notation consistent with Proposition 7.1, we define, for $\vec {\textbf{x}}\in \mathbb {A}_k$,

9.2 $$\begin{aligned} \alpha _k(\vec {\textbf{x}}) =\alpha _{k,\varepsilon } (\vec {\textbf{x}}) = G_{N,\beta }\Big (\,\big \{ \boldsymbol{\sigma }\in B(\vec {\textbf{x}},\varepsilon ):\,f_{N}(\boldsymbol{\sigma })\in A \big \} \Big )/ G_{N,\beta }(B(\vec {\textbf{x}},\varepsilon )), \end{aligned}$$

so that, in the notation of Section 7.3,

$$ \Pi ^{\varepsilon }_{N,\beta }\big (f_{N}(\boldsymbol{\sigma })\in A \big )\wedge 1 = \alpha ^{\varepsilon }_{0,k}(0) \wedge 1. $$

Written more explicitly in terms of $H_N(\boldsymbol{\sigma })$ and $\bar{f}_N(\boldsymbol{\sigma },H_N)$, the function $\alpha _{k,\varepsilon } (\vec {\textbf{x}})$ is given by

9.3 $$\begin{aligned} \alpha _{k,\varepsilon } (\vec {\textbf{x}}) =\frac{\int _{B(\vec {\textbf{x}},\varepsilon )}1\!\!1\{ \bar{f}_{N}(\boldsymbol{\sigma },H_N)\in A \}e^{\beta H_N(\boldsymbol{\sigma })}d\boldsymbol{\sigma }}{\int _{B(\vec {\textbf{x}},\varepsilon )}e^{\beta H_N(\boldsymbol{\sigma })}d\boldsymbol{\sigma }}. \end{aligned}$$

Note that $\alpha _{k,\varepsilon }$ is a.s. continuous on $\mathbb {A}_k$ by bounded convergence, since the volume of the symmetric difference $B(\vec {\textbf{x}},\varepsilon )\vartriangle B(\vec {\textbf{x}}',\varepsilon )$ goes to zero as $\vec {\textbf{x}}\rightarrow \vec {\textbf{x}}'$. Bounded convergence, together with (1.23), also implies the continuity property of (7.2). In (9.3) we have $\alpha _{k,\varepsilon }(\vec {\textbf{x}})$ in the form $\bar{\alpha }_{k,\varepsilon }(\vec {\textbf{x}},H_N(\cdot ))$ for deterministic $\bar{\alpha }_{k,\varepsilon }$. By a change of variables, its rotational invariance is inherited from $\bar{f}_N$. Further, $\alpha _{k,\varepsilon }(\vec {\textbf{x}}_{k}^{\textbf{e}})\le 1$ a.s. and hence, by Proposition 7.1, we have that the lhs of (9.1) is bounded by the sum of

9.4 $$\begin{aligned} \varlimsup _{\varepsilon \rightarrow 0} \sum _{m=0}^{k-1}\sup _{I_m(\varepsilon )\times I'_m(\varepsilon )}\Theta _{\xi _{(m)}}(E,R) \end{aligned}$$

and

9.5 $$\begin{aligned} \varlimsup _{\varepsilon \rightarrow 0} \varlimsup _{N\rightarrow \infty }\frac{1}{N}\log \Big (\sup _{\bar{V}(\varepsilon )}\mathbb {E}\big [ \alpha _{k,\varepsilon }(\vec {\textbf{x}}_{k}^{\textbf{e}}) \,\big |\,\mathrm{\textsf {CP}}(\vec {\textbf{x}}_{k}^\textbf{e},\vec {E},\vec {R}) \big ]\Big ). \end{aligned}$$

Recall that, since $(E,R)\mapsto \Theta _{\xi }(E,R)$ is continuous and (1.21) holds, by Corollary 6.9, (9.4) is zero, so we finish the proof upon showing that (9.5) is negative. By standard diagonalization argument, it suffices to consider in (9.5) only the limit superior in *N*, for a fixed, arbitrary sequence $\varepsilon _{N}\rightarrow 0$. That is, it suffices to show that

9.6 $$\begin{aligned} \varlimsup _{N\rightarrow \infty }\frac{1}{N}\log \Big (\sup _{\bar{V}(\varepsilon _N)} \mathbb {E}\big [ \alpha _{k,\varepsilon _N}(\vec {\textbf{x}}_{k}^{\textbf{e}}) \,\big |\,\mathrm{\textsf {CP}}(\vec {\textbf{x}}_{k}^\textbf{e},\vec {E},\vec {R}) \big ] \Big )<0. \end{aligned}$$

To this end, suppose that $F_{N,\beta }^\gamma$ is the free energy of the Gibbs measure $G^{\gamma }_{N,\beta }$ associated to $H_N^\gamma$, where $\big (H^\gamma _N(\boldsymbol{\sigma })\big )_{\gamma \in \Gamma }$ is, for each *N*, a family of Gaussian fields on the same probability reference space $(\Sigma _N,\mathcal {F}_N,\mu _N)$, such that for some fixed v>0,

9.7 $$\begin{aligned} \lim _{N\rightarrow \infty }\sup _{\gamma \in \Gamma ,\boldsymbol{\sigma }\in \bar{\Sigma }_N}\Big |\frac{1}{N}\mathbb {E}\big [H^\gamma _N(\boldsymbol{\sigma })^2\big ]-v\Big |&= \lim _{N\rightarrow \infty }\sup _{\gamma \in \Gamma ,\boldsymbol{\sigma }\in \bar{\Sigma }_N}\Big |\frac{1}{N}\mathbb {E}\big [H^\gamma _N(\boldsymbol{\sigma })\big ]\Big | \nonumber \\&= \lim _{N\rightarrow \infty } \sup _{\gamma \in \Gamma } \, \big |\mathbb {E}F_{N,\beta }^{\gamma }-\frac{1}{2}\beta ^2v \big |=0\,, \end{aligned}$$

for some measurable subset $\bar{\Sigma }_N\subset \Sigma _N$ such that $\mu _N(\bar{\Sigma }_N)=1$. Let $\bar{f}_N(\boldsymbol{\sigma },\varphi )$ be a deterministic function from $\bar{\Sigma }_{N}\times \mathbb {R}^{\Sigma _{N}}$ to $\mathbb {R}$ such that $\bar{f}_N(\boldsymbol{\sigma },H_N^\gamma )$ is a.s. measurable. By essentially the same proof as for Theorem 1.2, if, for some measurable $A\subset \mathbb {R}$,

9.8 $$\begin{aligned} \varlimsup _{\varepsilon \rightarrow 0}\varlimsup _{N\rightarrow \infty }\frac{1}{N}\log \Big \{\sup _{\begin{array}{c} \gamma \in \Gamma \\ \boldsymbol{\sigma }\in \bar{\Sigma }_{N} \end{array}} \sup _{|E-\beta v|<\varepsilon }\mathbb {P}\big (\bar{f}_{N}(\boldsymbol{\sigma },H_N^\gamma )\in A\,\big |\,\frac{1}{N}H^{\gamma }_{N}(\boldsymbol{\sigma })=E\big )\Big \}<0, \end{aligned}$$

then

9.9 $$\begin{aligned} \varlimsup _{N\rightarrow \infty }\frac{1}{N}\log \Big ( \sup _{\gamma \in \Gamma }\mathbb {E}\big [ G^{\gamma }_{N,\beta }\left( \bar{f}_{N}(\boldsymbol{\sigma },H_N^{\gamma })\in A\right) \big ] \Big ) <0\,. \end{aligned}$$

Indeed, with v=|Γ|=1 and $\mathbb {E}H^\gamma _N(\boldsymbol{\sigma })=0$, the bound (9.9) follows from Theorem 1.2 (see (1.12)) applied with $\bar{\Sigma }_N$. Generalizing to general v>0 is just a matter of scaling, see (1.7). By going over the proof, one can check that allowing for vanishing expectation and uniform bound over γ for |Γ|>1 only requires straightforward modifications.

Our next lemma, whose proof is deferred to the end of the section, verifies (9.7) and thereby allows us to employ the above, for $v=\bar{\xi }_{(k)}(1)$ of (6.6), $\Gamma =\bar{V}(\varepsilon _{N})$, $\bar{\Sigma }_N=B(\vec {\textbf{x}}_{k}^\textbf{e},\varepsilon _N)$, $\Sigma _{N}=\mathbb {S}^{N-1}$ where $H_N^\gamma (\cdot )$ at $\gamma =(\vec {E},\vec {R})$ follows the conditional law of $H_N(\cdot )-NE_{\star }(q_k)$ given $\mathrm{\textsf {CP}}(\vec {\textbf{x}}_{k}^\textbf{e},\vec {E},\vec {R})$.

### Lemma 9.1

Suppose that $\hat{H}_N(\boldsymbol{\sigma };\vec {E},\vec {R})$ is a Gaussian field having the law of $H_N(\boldsymbol{\sigma })-NE_{\star }(q_k)$ conditional on $\mathrm{\textsf {CP}}(\vec {\textbf{x}}_{k}^\textbf{e},\vec {E},\vec {R})$. Then,

9.10 $$\begin{aligned} \lim _{N\rightarrow \infty }\sup _{\bar{V}(\varepsilon _{N})}\sup _{\boldsymbol{\sigma }\in B(\vec {\textbf{x}}_{k}^\textbf{e},\varepsilon _N)}&\Big |\frac{1}{N} \mathbb {E}\big [ \hat{H}_N(\boldsymbol{\sigma };\vec {E},\vec {R}) \big ]\Big | =0, \end{aligned}$$

9.11 $$\begin{aligned} \lim _{N\rightarrow \infty }\sup _{\bar{V}(\varepsilon _{N})}\sup _{\boldsymbol{\sigma }\in B(\vec {\textbf{x}}_{k}^\textbf{e},\varepsilon _N)}&\Big |\frac{1}{N} \mathbb {E}\big [\hat{H}_N(\boldsymbol{\sigma };\vec {E},\vec {R})^2\big ]- \bar{\xi }_{k}(1)\,\Big |=0, \end{aligned}$$

9.12 $$\begin{aligned} \lim _{N\rightarrow \infty }\sup _{\bar{V}(\varepsilon _{N})}&\bigg |\, \mathbb {E}\bigg [\frac{1}{N}\log \Big ( \int _{B(\vec {\textbf{x}}_{k}^\textbf{e},\varepsilon _N)} e^{\beta \hat{H}_N(\boldsymbol{\sigma };\vec {E},\vec {R})}d\boldsymbol{\sigma }\Big ) \bigg ]-\frac{1}{2}\beta ^2\bar{\xi }_k(1)\, \bigg |=0, \end{aligned}$$

where here $d\boldsymbol{\sigma }$ denotes integration wrt the normalized to 1 measure on $B(\vec {\textbf{x}}_k^{\textbf{e}},\varepsilon _N)$.

From (9.3), we may write the expectation in (9.6) as

$$ \mathbb {E}\Bigg [ \frac{\int _{B(\vec {\textbf{x}}_{k}^\textbf{e},\varepsilon _N)}1\!\!1\Big \{ \bar{f}_{N}(\boldsymbol{\sigma }, \hat{H}_N(\,\cdot \,;\vec {E},\vec {R}) + NE_{\star }(q_k))\in A \Big \}e^{\beta \hat{H}_N(\boldsymbol{\sigma };\vec {E},\vec {R})}d\boldsymbol{\sigma }}{\int _{B(\vec {\textbf{x}}_{k}^\textbf{e},\varepsilon _N)}e^{\beta \hat{H}_N(\boldsymbol{\sigma };\vec {E},\vec {R})}d\boldsymbol{\sigma }} \Bigg ] \,. $$

Thus, (9.6), which is of the form of (9.9), holds, once we verify the relevant version of (9.8). That is, the proof will be complete upon showing that

9.13 $$\begin{aligned} \varlimsup _{\varepsilon \rightarrow 0}\varlimsup _{N\rightarrow \infty }\frac{1}{N}\log \Big \{ \sup _{\bar{V}(\varepsilon _N)}\sup _{\boldsymbol{\sigma }\in B(\vec {\textbf{x}}_{k}^\textbf{e},\varepsilon _N)}\sup _{|E-\beta \bar{\xi }_k(1)|<\varepsilon } P(\boldsymbol{\sigma },A;E,\vec {E},\vec {R}) \Big \} <0, \end{aligned}$$

where, written in terms of $\tilde{H}_N(\cdot ):= \hat{H}_N(\cdot )+N E_{\star }(q_k)$, we have that

$$\begin{aligned} P(\boldsymbol{\sigma },A;E,\vec {E},\vec {R})&:\!=\! \mathbb {P}\big (\bar{f}_{N}(\boldsymbol{\sigma }, \tilde{H}_N(\,\cdot \,;\vec {E},\vec {R}) ) \in A\,\big |\,\frac{1}{N} \tilde{H}_N(\boldsymbol{\sigma };\vec {E},\vec {R})\!=\!E\!+\!E_{\star }(q_k)\big ) \\&= \mathbb {P}\big (\bar{f}_{N}(\boldsymbol{\sigma }, H_N) \in A\,\big |\, \mathrm{\textsf {CP}}(\boldsymbol{\sigma }, \vec {\textbf{x}}_{k}^\textbf{e},E+E_{\star }(q_k),\vec {E},\vec {R}) \big ). \end{aligned}$$

Note that (9.13) follows directly from our assumption (1.26) (see also (1.25)), provided that

9.14 $$\begin{aligned} F'(\beta ,\xi )=E_{\star }(q_k)+\beta \bar{\xi }_{(k)}(1) \end{aligned}$$

(where we denote by $F(\beta ,\xi )$ the limiting free energy of the spherical model with mixture ξ). To verify (9.14), recall (1.21), (6.5), and that by the tap representation of [42, Theorem 5], for any q∈[0,1)

$$ F(\beta ,\xi ) \ge \beta E_{\star }(q) +\frac{1}{2}\log (1-q)+F(\beta ,\bar{\xi }_{q}) \,, $$

with equality for $q\in \mathcal {S}_P$. Consequently, $F'(\beta ,\xi )$ equals the partial derivative in β of the rhs at $q_k\in \mathcal {S}_P$. That is,

9.15 $$\begin{aligned} F'(\beta ,\xi ) = E_{\star }(q_k) +\frac{d}{d\beta }F(\beta ,\bar{\xi }_{(k)}) \end{aligned}$$

(as $$\bar{\xi }_{(k)}=\bar{\xi }_{q_k}$$, see (6.6)). From [42, Corollary 6] we have that $$F(\beta ,\bar{\xi }_{(k)})=\frac{1}{2}\beta ^2\bar{\xi }_{(k)}(1)$$, so β is bounded by the critical inverse-temperature of the mixture $$\bar{\xi }_{(k)}(t)$$. If β is strictly less than the critical inverse-temperature then $$F(\beta ',\bar{\xi }_{(k)})=\frac{1}{2}\beta '^2\bar{\xi }_{(k)}(1)$$ for all $\beta^{\prime }$ in a neighborhood of β and obviously $$\frac{d}{d\beta }F(\beta ,\bar{\xi }_{(k)})=\beta \bar{\xi }_{(k)}(1)$$. In fact, the last identity holds even if β is the critical inverse-temperature, see the beginning of Section 4. Upon comparing (9.14) with (9.15), it remains to prove Lemma 9.1. □

### Proof of Lemma 9.1

Recall that $B(\vec {\textbf{x}}_k^{\textbf{e}},0) = \mathbb {S}^{N-1}\cap S_0(\vec {\textbf{x}}_k^{\textbf{e}})$ for $S_0(\cdot )$ of (7.6). Thus, by Lemma 7.3 we have that conditional on $\mathrm{\textsf {CP}}(\vec {\textbf{x}}_{k}^\textbf{e},\vec {E},\vec {R})$, the Gaussian field $H_N(\boldsymbol{\sigma })-NE_k$ has on $B(\vec {\textbf{x}}_k^{\textbf{e}},0)$ zero mean and covariance $N \xi _{q_k}(\langle \boldsymbol{\sigma },\boldsymbol{\sigma }'\rangle /N - q_k)$. Mapping $B(\vec {\textbf{x}}_k^{\textbf{e}},0)$ to $\mathbb {S}^{N-k-1}$ by $\boldsymbol{\sigma }\mapsto \frac{1}{\sqrt{1-q_k}} (\boldsymbol{\sigma }- \textbf{x}_k^{\textbf{e}})$, yields a conditional centered field which is a spherical model of the mixture $\bar{\xi }_{(k)}$ of (6.6). This spherical model has normalized variance $\bar{\xi }_{(k)}(1)$ and the limiting free energy $\frac{1}{2}\beta ^2\bar{\xi }_{(k)}(1)$ (by [42, Corollary 6]), resulting with (9.10)-(9.12) when $\boldsymbol{\sigma }$ is restricted from $B(\vec {\textbf{x}}_k^{\textbf{e}},\varepsilon _N)$ to $B(\vec {\textbf{x}}_k^{\textbf{e}},0)$.

To deal with the larger bands $B(\vec {\textbf{x}}_k^{\textbf{e}},\varepsilon _N)$, note that conditionally on $\mathrm{\textsf {CP}}(\vec {\textbf{x}}_{k}^\textbf{e},\vec {E},\vec {R})$, the field $H_N(\boldsymbol{\sigma })$ can be written as the sum of two parts

$$\begin{aligned} N \varPhi _N(\boldsymbol{\sigma }; \vec {E},\vec {R}) + H_N^0(\boldsymbol{\sigma }) := \mathbb {E}\Big [ H_N(\boldsymbol{\sigma }) \,\Big |\,\mathrm{\textsf {CP}}(\vec {\textbf{x}}_{k}^\textbf{e},\vec {E},\vec {R}) \Big ] + H_N^0(\boldsymbol{\sigma }), \end{aligned}$$

where the centered field $H_N^0(\boldsymbol{\sigma })$ has the law as $H_N(\boldsymbol{\sigma })$ conditional on $\mathrm{\textsf {CP}}(\vec {\textbf{x}}_{k}^\textbf{e},\vec {0},\vec {0})$. By Anderson’s inequality, see Remark 2.4, the Lipschitz constant of $H_N^0(\boldsymbol{\sigma })$ over the sphere is stochastically dominated by that of $H_N(\boldsymbol{\sigma })$. The latter is bounded by $L\sqrt{N}$ with probability at least $1-e^{-CN}$ for some constants L=L(ξ) and C=C(ξ), see e.g. [17, Lemma 6.1] or [34, Lemma A.3]. Thus, for some $\delta _N\rightarrow 0$, on $B(\vec {\textbf{x}}_k^{\textbf{e}},\varepsilon _N)$, the normalized conditional variance $\frac{1}{N}\mathbb {E}\big [ H^0_N(\boldsymbol{\sigma })^2]$ is uniformly within $\delta _N$ from its value $\bar{\xi }_{(k)}(1)$ on $B(\vec {\textbf{x}}_k^{\textbf{e}},0)$. Further, by the same Lipschitz property, one easily sees that in the $N\rightarrow \infty$ limit, the free energy of $H^0_N(\boldsymbol{\sigma })$ on the band $B(\vec {\textbf{x}}_k^{\textbf{e}},\varepsilon _N)$ is the same as the one computed over $B(\vec {\textbf{x}}_k^{\textbf{e}},0)$ (treated as a sphere with the uniform measure on it). The proof is thus completed by showing that for some $\delta _N \rightarrow 0$,

9.16 $$\begin{aligned} \sup _{(\vec {E},\vec {R})\in \bar{V}(\varepsilon _{N})} \sup _{\boldsymbol{\sigma }\in B(\vec {\textbf{x}}_k^{\textbf{e}},\varepsilon _N)} \big |\varPhi _N(\boldsymbol{\sigma }; \vec {E},\vec {R})-E_k \big | \le \delta _N \,, \end{aligned}$$

where $E_k$ denotes the *k*-th element of $\vec {E}$. Proceeding to prove (9.16), we set $U_{\boldsymbol{\sigma }}=\text{ span }\{U,\boldsymbol{\sigma }\}$ for $U=\text{ span }\{\textbf{x}_i^{\textbf{e}}\}_{i\le k}=\text{ span }\{\textbf{e}_i\}_{i\le k}$, letting $\bar{W}_N=\{\partial _\textbf{v}H_N(\textbf{x}_a^{\textbf{e}})\}_{a\le k,\textbf{v}\in U_{\boldsymbol{\sigma }}^{\perp }}$ and

$$\begin{aligned} W_N&=\bigg \{\frac{1}{N} H_N(\textbf{x}_i^{\textbf{e}}), \frac{\partial _{\textbf{x}_i^{\textbf{e}}-\textbf{x}_{i-1}^{\textbf{e}}} H_N(\textbf{x}_i^{\textbf{e}})}{\Vert \textbf{x}_i^{\textbf{e}}-\textbf{x}_{i-1}^{\textbf{e}}\Vert ^2}, \frac{\partial _{\boldsymbol{\sigma }-\textbf{x}_i^{\textbf{e}}}H_N(\textbf{x}_i^{\textbf{e}})}{\Vert \boldsymbol{\sigma }-\textbf{x}_i^{\textbf{e}}\Vert ^2} \bigg \}_{1 \le i\le k}\\&\quad \bigcup \bigg \{\frac{1}{\sqrt{N}} \partial _{\textbf{e}_j} H_N(\textbf{x}_i^{\textbf{e}}) \bigg \}_{1 \le i< j\le k} \,. \end{aligned}$$

We claim that the Gaussian vectors $(W_N,H_N(\boldsymbol{\sigma })/N)$ and $\bar{W}_N$ are independent. Indeed, similarly to (7.10) and (7.13), we have that

9.17 $$\begin{aligned} \begin{aligned} \mathbb {E}\left\{ \partial _\textbf{v}H_N(\textbf{x}_a^{\textbf{e}}) H_N(\textbf{x}) \right\}&= \xi '([ \textbf{x}_a^{\textbf{e}},\textbf{x}]) \langle \textbf{v},\textbf{x}\rangle ,\\ \mathbb {E}\left\{ \partial _\textbf{v}H_N(\textbf{x}_a^{\textbf{e}}) \partial _{\textbf{u}} H_N(\textbf{x}_i^{\textbf{e}}) \right\}&= \xi '([ \textbf{x}_a^{\textbf{e}},\textbf{x}_i^{\textbf{e}} ]) \langle \textbf{v},\textbf{u}\rangle + N^{-1}\xi ''([ \textbf{x}_a^{\textbf{e}},\textbf{x}_i^{\textbf{e}} ]) \langle \textbf{v},\textbf{x}_i^{\textbf{e}} \rangle \langle \textbf{x}_a^{\textbf{e}},\textbf{u}\rangle , \end{aligned} \end{aligned}$$

and the inner products involving $\textbf{v}$ are zero for $\textbf{x}\in \{\boldsymbol{\sigma },\textbf{x}_i^{\textbf{e}}\}_{i\le k}$ and $\textbf{u}\in \{\boldsymbol{\sigma },\textbf{e}_j,\textbf{x}_i^{\textbf{e}}\}_{i,j\le k}$.

This means that although the event $\mathrm{\textsf {CP}}(\vec {\textbf{x}}_{k}^\textbf{e},\vec {E},\vec {R})$ specifies all variables in $W_N\cup \bar{W}_N$, it is only those in $W_N$ that affect the value of $\varPhi _N(\boldsymbol{\sigma }; \vec {E},\vec {R})$.

Using the well-known formula for the conditional expectation of Gaussian variables,

9.18 $$\begin{aligned} \varPhi _N(\boldsymbol{\sigma }; \vec {E},\vec {R}) = \Big [\mathbb {E}\big (W_N H_N(\boldsymbol{\sigma }) \big )\Big ]^{\top } \Big [ N \mathbb {E}\big ( W_N W_N^{\top }\big )\Big ]^{-1} \varPsi (\vec {E},\vec {R}), \end{aligned}$$

where here we treat $W_N$ as a vector of length $\frac{k(k+5)}{2}$ and $\varPsi (\vec {E},\vec {R})$ is a vector whose values are $E_i$ and $R_i$ in the entries corresponding to $\frac{1}{N} H_N(\textbf{x}_i^{\textbf{e}})$ and $\frac{\partial _{\textbf{x}_i^{\textbf{e}}-\textbf{x}_{i-1}^{\textbf{e}}} H_N(\textbf{x}_i^{\textbf{e}})}{\Vert \textbf{x}_i^{\textbf{e}}-\textbf{x}_{i-1}^{\textbf{e}}\Vert ^2}$ and 0 elsewhere.

From the proof of

Lemma 7.3 or directly from (9.17),

one sees that for $\boldsymbol{\sigma }\in B(\vec {\textbf{x}}_k^{\textbf{e}},0)$ the vector $\mathbb {E}\big (W_N H_N(\boldsymbol{\sigma }) \big )$ and the invertible^11^ matrix $N \mathbb {E}(W_N W_N^{\top })$ depend neither on $\boldsymbol{\sigma }$ nor on *N*. For $\boldsymbol{\sigma }\in B(\vec {\textbf{x}}_k^{\textbf{e}},\varepsilon _N)$, the vector $\mathbb {E}\big (W_N H_N(\boldsymbol{\sigma }) \big )$ and the matrix $N \mathbb {E}(W_N W_N^{\top })$ match their values on $B(\vec {\textbf{x}}_k^{\textbf{e}},0)$, up to a small perturbation which depends on the vector $\big (\langle \boldsymbol{\sigma },\textbf{x}_i^{\textbf{e}}\rangle /N-q_i\big )_{i\le k}$ whose elements are bounded by $\varepsilon _N$ in absolute value. With $\varPsi (\vec {E},\vec {R})$ independent of $\boldsymbol{\sigma }$ and *N*, this extends to the lhs of (9.18), thereby yielding (9.16) (where we have also utilized the compactness of $\bar{V}(\varepsilon _{N})$). □

## Data Availability

No datasets were generated or analysed during the current study.

## Statistics relative to pure states

To extend the ckchs-equations (which as derived apply only for iid initial conditions), for Langevin dynamics (1.5) initialized according to the Gibbs measure, one must deal with more general statistics that are measured relative to the path $\vec {\textbf{x}}$ with which a pure state is defined (see Proposition 3.1). We thus derive here the corresponding generalization of Theorem 1.4 (which is indeed used in [24], see Section 2.1).

Assume the strict *k*-rsb condition (1.21) and suppose that $\bar{f}_N(\boldsymbol{\sigma },\vec {\textbf{x}},\varphi )$ is a deterministic function from $\mathbb {S}^{N-1}\times \mathbb {A}_k\times C^\infty (\mathbb {B}^{N})$ to $\mathbb {R}$, which is rotationally invariant in the sense that $\bar{f}_N(\boldsymbol{\sigma },\vec {\textbf{x}}, \varphi (\cdot ))= \bar{f}_N(O\boldsymbol{\sigma },O\vec {\textbf{x}},\varphi (O^T\cdot ))$ for any orthogonal $O\in \mathbb {R}^{N\times N}$. The continuity property of (1.23) is replaced here by having for smooth $\varphi ,\psi _i\in C^{\infty }(\mathbb {B}^{N})$,

A.1 $$\begin{aligned} \lim _{t_{i}\rightarrow 0}\bar{f}_{N}\Big (\boldsymbol{\sigma },\vec {\textbf{x}},\varphi +\sum _{i\le k}t_{i}\psi _{i}\Big )=\bar{f}_{N}(\boldsymbol{\sigma },\vec {\textbf{x}},\varphi )\quad \text{ and }\quad \lim _{\vec {\textbf{x}}'\rightarrow \vec {\textbf{x}}}\bar{f}_{N}(\boldsymbol{\sigma },\vec {\textbf{x}}',\varphi )=\bar{f}_{N}(\boldsymbol{\sigma },\vec {\textbf{x}},\varphi ). \end{aligned}$$

Define the subset $\mathscr {B}(\varepsilon )=\cup _{\vec {\textbf{x}}\in \mathscr {C}_k(\varepsilon )}B(\vec {\textbf{x}},\varepsilon )$. By Proposition 3.1, there exists a sequence $\varepsilon _{N}\rightarrow 0$ such that $\lim _{N\rightarrow \infty }G_{N,\beta }(\mathscr {B}(\varepsilon _N))=1$, in probability. Suppose $\vec {\textbf{x}}(\cdot )$ is a random function from $\mathscr {B}(\varepsilon _N)$ to $\mathscr {C}_k(\varepsilon _N)$ such that $\boldsymbol{\sigma }\in B(\vec {\textbf{x}}(\boldsymbol{\sigma }),\varepsilon _{N})$.

### Corollary A.1

Consider the spherical mixed *p*-spin model with a generic mixture ξ(t) and $\beta >\beta _{c}$ at which ξ(t) is strictly *k*-rsb.

Suppose that $f_N(\boldsymbol{\sigma },\vec {\textbf{x}})=\bar{f}_N(\boldsymbol{\sigma },\vec {\textbf{x}},H_N(\cdot ))$ for some deterministic rotationally invariant $\bar{f}_N(\boldsymbol{\sigma },\vec {\textbf{x}},\varphi )$ which satisfies the continuity property of (A.1) and that $f_N(\boldsymbol{\sigma },\vec {\textbf{x}}(\boldsymbol{\sigma }))$ is $\mathbb {P}$-a.s. a measurable function on $\mathbb {S}^{N-1}$. If for some measurable $A\subset \mathbb {R}$,

A.2 $$\begin{aligned} \varlimsup _{\varepsilon \rightarrow 0}\varlimsup _{N\rightarrow \infty }\sup _{B(\vec {\textbf{x}}^{\textbf{e}}_k,\varepsilon )\times V(\varepsilon )}\frac{1}{N}\log \mathbb {P}\left( \, f_{N}(\boldsymbol{\sigma },\vec {\textbf{x}}^{\textbf{e}}_k)\in A\,\Big |\,\mathrm{\textsf {CP}}(\boldsymbol{\sigma },\vec {\textbf{x}}^{\textbf{e}}_k,E,\vec {E},\vec {R})\,\right) <0, \end{aligned}$$

then

$$\begin{aligned} \lim _{N\rightarrow \infty }\mathbb {E}G_{N,\beta }\Big (\big \{ \boldsymbol{\sigma }\in \mathscr {B}(\varepsilon _{N}):\,f_{N}(\boldsymbol{\sigma },\vec {\textbf{x}}(\boldsymbol{\sigma }))\in A\big \}\Big )=0. \end{aligned}$$

### Proof

On $$\mathscr {B}(\varepsilon _{N})$$,

$$ 1\!\!1\{f(\boldsymbol{\sigma },\vec {\textbf{x}}(\boldsymbol{\sigma }))\in A\}\le \sum _{\vec {\textbf{x}}\in \mathscr {C}_k(\varepsilon _{N})} 1\!\!1\{f(\boldsymbol{\sigma },\vec {\textbf{x}})\in A,\,\boldsymbol{\sigma }\in B(\vec {\textbf{x}},\varepsilon _{N}) \} $$

and therefore

$$\begin{aligned}&G_{N,\beta }\Big (\big \{ \boldsymbol{\sigma }\in \mathscr {B}(\varepsilon _{N}):\,f_{N}(\boldsymbol{\sigma },\vec {\textbf{x}}(\boldsymbol{\sigma }))\in A\big \}\Big )\\&\le \sum _{\vec {\textbf{x}}\in \mathscr {C}_k(\varepsilon _{N})} G_{N,\beta }\Big (\big \{ \boldsymbol{\sigma }\in B(\vec {\textbf{x}},\varepsilon _{N}):\,f_{N}(\boldsymbol{\sigma },\vec {\textbf{x}})\in A\big \}\Big ) \\&\le \sum _{\vec {\textbf{x}}\in \mathscr {C}_k(\varepsilon _{N})} G_{N,\beta }\Big ( f_{N}(\boldsymbol{\sigma },\vec {\textbf{x}})\in A\,\Big |\,B(\vec {\textbf{x}},\varepsilon _{N})\Big ). \end{aligned}$$

Defining, analogously to (9.2),

$$ \alpha _{k}(\vec {\textbf{x}}) = \alpha _{k,\varepsilon }(\vec {\textbf{x}})= G_{N,\beta }\Big ( f_{N}(\boldsymbol{\sigma },\vec {\textbf{x}})\in A\,\Big |\,B(\vec {\textbf{x}},\varepsilon )\Big ), $$

and using the notation of Section 7.3, we thus complete the proof by showing that for any $\varepsilon _N \rightarrow 0$,

$$\begin{aligned} \varlimsup _{N\rightarrow \infty }\frac{1}{N}\log \mathbb {E}\Big [ \alpha ^{\varepsilon _N}_{0,k}(0)\wedge 1\Big ] <0. \end{aligned}$$

Similarly to the proof of Theorem 1.4, the preceding can be shown from (A.2) using Proposition 7.1. We note that the second assumption in (A.1) is required here for the continuity of $\vec {\textbf{x}}\mapsto \alpha _{k,\varepsilon }(\vec {\textbf{x}})$. □

## Footnotes

1 Let $\beta '<\beta$ and $\beta ''=\sqrt{\beta ^{2}-\beta '^{2}}$. Note that $\beta H_{N}(\boldsymbol{\sigma })$ has the same law as $\beta 'H^{(1)}_{N}(\boldsymbol{\sigma })+\beta ''H^{(2)}_{N}(\boldsymbol{\sigma })$ for two independent copies $H_N^{(i)}(\cdot )$, i=1,2 of $H_N(\cdot )$. Hence, from Jensen’s inequality and (1.7),

$$ \mathbb {E}F_{N,\beta }\le \mathbb {E}F_{N,\beta '}+\frac{1}{N}\log \mathbb {E}Z_{N,\beta ''} +o(1). $$

It follows that if β satisfies the equality in (1.8), then so does $\beta^{\prime }$. By Hölder’s inequality, $$F_{N,\beta }$$ is convex and therefore equality as in (1.8) holds also at the critical $$\beta =\beta _c$$.

2 Applying with $f(q')=E_{\star }(q')$, $g(q')=\mathcal {P}_{\hat{\xi }_{q'},\infty }(\alpha _q,c_q)$ and $x_0=q$ the fact that if f(x)≤g(x) are real functions with *f*(*x*) convex, *g*(*x*) differentiable at $x_0$ and $f(x_0)=g(x_0)$ then $f'(x_0)$ exists and is equal to $g'(x_0)$.

3 For such $\vec {\textbf{x}}$ to exist, we must have that N≥k. Henceforth, we always implicitly assume that indeed N≥k.

4 Since the variables (2.12) are jointly Gaussian, $C(q_1)$ is invertible if no linear combination of them is deterministically zero. Recall that $0<q_1<1$, so working directly with the formula (1.14) for the Hamiltonian, one can easily verify that this is indeed the case for our choice of $\boldsymbol{\sigma }_1$ and $\textbf{x}_1$.

5 For this, in [42] it is assumed that $|\text{ supp }(\nu _P)|>1$. This must hold in our setting, since for $\beta >\beta _c$ we have from the Parisi formula that $\nu _P\ne \delta _0$, and from (1.21) also that $0\in \text{ supp }(\nu _P)$.

6 The expectation in the Kac-Rice formula also includes the determinant of the Hessian. However, this determinant concentrates so well that at the exponential scale in which we work here, it is not difficult to deal with.

7 Note that in the pure case, $\langle \nabla H_N(\boldsymbol{\sigma }),\boldsymbol{\sigma }\rangle = pH_N(\boldsymbol{\sigma })$.

8 In fact, as can be verified by going through the proof, it is enough to work only on the set $\{\textbf{x}_{m+1}: (\vec {\textbf{x}}_m^{\textbf{e}},\textbf{x}_{m+1})\in \mathbb {A}_{m,m+1}\}$ and require continuity and finiteness of the second moment of $w_N(\textbf{x})$ only there.

9 Let i<j and note that $T\circ O_{i+1}\circ T\cdots O_{j-1}\circ T(\textbf{z}_j)$ is orthogonal to $\textbf{e}_{N-i+1}$ (simply by the operation of the left-most *T*). Hence, $O_{i}\circ T\circ O_{i+1}\circ T\cdots O_{j-1}\circ T(\textbf{z}_j)$ is orthogonal to $O_{i}(\textbf{e}_{N-i+1})=\textbf{z}_i$ and $O_{1}\circ T\circ O_{2}\circ T\cdots O_{j-1}\circ T(\textbf{z}_j)$ is orthogonal to $O_{1}\circ T\circ O_{2}\circ T\cdots O_{i-1}\circ T(\textbf{z}_i)$. From this we have that indeed $\Phi _m(\vec {\textbf{z}})\in \mathbb {A}_m$. Of course it is a bijection since if $\vec {\textbf{z}}$ and $\vec {\textbf{z}}'$ are such that $\textbf{z}_i=\textbf{z}_i'$ for all i≤j-1 and $\textbf{z}_j\ne \textbf{z}_j'$ then $\Phi _{m,j}(\vec {\textbf{z}})\ne \Phi _{m,j}(\vec {\textbf{z}}')$.

10 We note that to use (7.34), here we view $\alpha _{m+1,n}^{\varepsilon ,\delta }(\vec {\textbf{x}})$ with $\vec {\textbf{x}}=(\vec {\textbf{x}}_m^{\textbf{e}},\textbf{x}_{m+1})$ as a function of $\textbf{x}_{m+1}$. Indeed, in the proofs of Lemma 7.6 and Corollary 7.7, we always work with $\textbf{x}_{m+1}$ such that $(\vec {\textbf{x}}_m^{\textbf{e}},\textbf{x}_{m+1})\in \mathscr {C}_{m,m+1}(\varepsilon ,\vec {\textbf{x}}_m^\textbf{e})$. See Footnote 8.

11 Here we use the fact that ξ is not a pure mixture, since we assume it is generic. If it is pure, then k=1 (see Remark 1.7), and $\partial _{\textbf{x}_1^{\textbf{e}}}H_N(\textbf{x}_1^{\textbf{e}})$ is measurable wrt $H_N(\textbf{x}_1^{\textbf{e}})$.

## References

[1] Achlioptas, D., Coja-Oghlan, A.: Algorithmic barriers from phase transitions. In: 2008 49th Annual IEEE Symposium on Foundations of Computer Science, pp. 793–802 (2008)

[2] Anderson, T.W.: The integral of a symmetric unimodal function over a symmetric convex set and some probability inequalities. Proc. Amer. Math. Soc. **6**, 170–176 (1955)

[3] Auffinger, A., Ben Arous, G.: Complexity of random smooth functions on the high-dimensional sphere. Ann. Probab. **41**(6), 4214–4247 (2013)

[4] Auffinger, A., Ben Arous, G., Černý, J.: Random matrices and complexity of spin glasses. Commun. Pure Appl. Math. **66**(2), 165–201 (2013)

[5] Auffinger, A., Chen, W.-K.: On concentration properties of disordered Hamiltonians. Proc. Amer. Math. Soc. **146**(4), 1807–1815 (2018)

[6] Auffinger, A., El Alaoui, E., Sellke, M.: On the discontinuous breaking of replica symmetry and shattering in mean-field spin glasses, (2025). arXiv preprint arXiv:2506.02238

[7] Auffinger, A., Zhou, Y.: The spherical spin glass at zero temperature. Commun. Math. Phys. **406**(12), 314 (2025)

[8] Barrat, A., Burioni, R., Mézard, M.: Dynamics within metastable states in a mean-field spin glass. J. Phys. A: Math. Gen. **29**, L81–L87 (1996)

[9] Barrat, A., Franz, S.: Basins of attraction of metastable states of the spherical -spin model. J. Phys. A: Math. Gen. **31**, L119–L127 (1998)

[10] Ben Arous, G., Dembo, A., Guionnet, A.: Aging of spherical spin glasses. Probab. Theory Relat. Fields **120**(1), 1–67 (2001)

[11] Ben Arous, G., Dembo, A., Guionnet, A.: Cugliandolo–Kurchan equations for dynamics of spin-glasses. Probab. Theory Relat. Fields **136**(4), 619–660 (2006)

[12] Ben Arous, G., Guionnet, A.: Large deviations for Wigner’s law and Voiculescu’s non-commutative entropy. Probab. Theory Relat. Fields **108**(4), 517–542 (1997)

[13] Ben Arous, G., Subag, E., Zeitouni, O.: Geometry and temperature chaos in mixed spherical spin glasses at low temperature: the perturbative regime. Commun. Pure Appl. Math. **73**(8), 1732–1828 (2020)

[14] Bouchaud, J.P., Cugliandolo, L.F., Kurchan, J., Mézard, M.: Out of equilibrium dynamics in spin-glasses and other glassy systems. In: Young, A.P. (ed.) Spin Glass Dynamics and Random Fields. World Scientific, Singapore (1997)

[15] Chen, W.-K.: The Aizenman–Sims–Starr scheme and Parisi formula for mixed -spin spherical models. Electron. J. Probab. **18**(94), 14 (2013)

[16] Chen, W.-K., Panchenko, D., Subag, E.: The generalized TAP free energy II. Commun. Math. Phys. **381**(1), 257–291 (2021)

[17] Chen, W.-K., Panchenko, D., Subag, E.: The generalized TAP free energy. Commun. Pure Appl. Math. **76**(7), 1329–1415 (2023)

[18] Chen, W.-K., Sen, A.: Parisi formula, disorder chaos and fluctuation for the ground state energy in the spherical mixed -spin models. Commun. Math. Phys. **350**(1), 129–173 (2017)

[19] Crisanti, A., Horner, H., Sommers, H.-J.: The spherical -spin interaction spin-glass model. Z. Physik B Condens. Matter **92**, 257–271 (1993)

[20] Crisanti, A., Sommers, H.-J.: The spherical p-spin interaction spin glass model: the statics. Z. Physik B Condens. Matter **87**(3), 341–354 (1992)

[21] Cugliandolo, L.F., Kurchan, J.: Analytical solution of the off-equilibrium dynamics of a long-range spin-glass model. Phys. Rev. Lett. **71**, 173 (1993) 10.1103/PhysRevLett.71.173 10054401

[22] Cugliandolo, L.F.: Course 7: Dynamics of glassy systems. In: Slow Relaxations and nonequilibrium dynamics in condensed matter, pp. 367–521. Springer (2003)

[23] Dembo, A., Subag, E.: Dynamics for spherical spin glasses: disorder dependent initial conditions. J. Stat. Phys. **181**(2), 465–514 (2020)

[24] Dembo, A., Subag, E.: Dynamics for spherical spin glasses: Gibbs distributed initial conditions. Preprint arXiv:2503.23342 (2025)

[25] El Alaoui, E., Montanari, A., Sellke, M.: Shattering in pure spherical spin glasses. Commun. Math. Phys. **406**, 111 (2025)

[26] Faraut, J.: Logarithmic potential theory, orthogonal polynomials, and random matrices. In: Modern methods in multivariate statistics, Lecture Notes of CIMPA-FECYT-UNESCO-ANR. Hermann (2014)

[27] Franz, S., Parisi, G.: Recipes for metastable states in spin glasses. J. Phys. I France **5**(11), 1401–1415 (1995)

[28] Ghirlanda, S., Guerra, F.: General properties of overlap probability distributions in disordered spin systems. Towards Parisi ultrametricity. J. Phys. A **31**(46), 9149–9155 (1998)

[29] Guerra, F., Toninelli, F.L.: The thermodynamic limit in mean field spin glass models. Commun. Math. Phys. **230**(1), 71–79 (2002)

[30] Huang, B., Sellke, M.: A constructive proof of the spherical Parisi formula. Preprint arXiv:2311.15495 (2023)

[31] Jagannath, A.: Approximate ultrametricity for random measures and applications to spin glasses. Commun. Pure Appl. Math. **70**(4), 611–664 (2017)

[32] Jagannath, A., Tobasco, I.: Low temperature asymptotics of spherical mean field spin glasses. Commun. Math. Phys. **352**(3), 979–1017 (2017)

[33] Jagannath, A., Tobasco, I.: Bounds on the complexity of Replica Symmetry Breaking for spherical spin glasses. Proc. Amer. Math. Soc. **146**(7), 3127–3142 (2018)

[34] Montanari, A., Subag, E.:Solving systems of Random Equations via First and Second-Order Optimization Algorithms, (2023). arXiv preprint arXiv:2306.13326

[35] Panchenko, D.: The Parisi ultrametricity conjecture. Ann. Math. (2) **177**(1), 383–393 (2013)

[36] Panchenko, D.: The Sherrington–Kirkpatrick Model. Springer Monographs in Mathematics, New York (2013)

[37] Parisi, G.: Infinite number of order parameters for spin-glasses. Phys. Rev. Lett. **43**, 1754–1756 (1979)

[38] Parisi, G.: A sequence of approximated solutions to the S-K model for spin glasses. J. Phys. A **13**(4), L115 (1980)

[39] Subag, E.: The complexity of spherical -spin models–A second moment approach. Ann. Probab. **45**(5), 3385–3450 (2017)

[40] Subag, E.: The geometry of the Gibbs measure of pure spherical spin glasses. Invent. Math. **210**(1), 135–209 (2017)

[41] Subag, E.: Convergence of the free energy for spherical spin glasses. J. Stat. Phys. **189**(2), 29 (2022)

[42] Subag, E.: Free energy landscapes in spherical spin glasses. Duke Math. J. **173**(7), 1291–1357 (2024)

[43] Subag, E., Zeitouni, O.: The extremal process of critical points of the pure -spin spherical spin glass model. Probab. Theory Relat. Fields **168**(3–4), 773–820 (2017)

[44] Subag, E., Zeitouni, O.: Concentration of the complexity of spherical pure -spin models at arbitrary energies. J. Math. Phys. **62**(12), 123301 (2021)

[45] Talagrand, M.: Free energy of the spherical mean field model. Probab. Theory Relat. Fields **134**(3), 339–382 (2006)

[46] Talagrand, M.: Construction of pure states in mean field models for spin glasses. Probab. Theory Relat. Fields **148**(3–4), 601–643 (2010)

[47] Thouless, D.J., Anderson, P.W., Palmer, R.G.: Solution of ‘solvable model of a spin glass’. Philos. Mag. **35**(3), 593–601 (1977)

[48] Zhou, Y.: On the Parisi measures of the spherical mixed -spin glass at zero temperature. Electron. J. Probab. **30**, 78 (2025)

